# New Tools in Heavy Metal Detection: Synthesis, Spectroscopic, and Quantum Chemical Characterization of Selected Water-Soluble Styryl Derivatives of Quinoline and 1,10-Phenanthroline

**DOI:** 10.3390/molecules30122659

**Published:** 2025-06-19

**Authors:** Jacek E. Nycz, Jolanta Kolińska, Nataliya Karaush-Karmazin, Tieqiao Chen, Maria Książek, Joachim Kusz

**Affiliations:** 1 Institute of Chemistry, Faculty of Science and Technology, University of Silesia in Katowice, ul. Szkolna 9, 40-006 Katowice, Poland; jolanta.kolinska@p.lodz.pl; 2 Institute of Polymer and Dye Technology, Faculty of Chemistry, Lodz University of Technology, Stefanowskiego 12/16, 90-924 Lodz, Poland; 3 Department of Chemistry and Nanomaterials Science, Bohdan Khmelnytsky National University, 18031 Cherkasy, Ukraine; 4 Ministry of Education Key Laboratory of Advanced Materials for Tropical Island Resources, Hainan Provincial Key Laboratory of Fine Chem, Hainan Provincial Fine Chemical Engineering Research Center, Hainan University, Haikou 570228, China; 5 Institute of Physics, Faculty of Science and Technology, University of Silesia in Katowice, 75 PułkuPiechoty 1a, 41-500 Chorzów, Poland; maria.ksiazek@us.edu.pl (M.K.); joachim.kusz@us.edu.pl (J.K.)

**Keywords:** phenanthroline, quinoline, water-soluble ligand, vinyl, styryl, DFT studies

## Abstract

A series of water-soluble molecules based on 8-isopropyl-2-methyl-5-nitroquinoline and 1,10-phenanthroline core were designed by introducing a π-conjugated bridge, vinyl unit –CH=CH–. We present the selective conversion of methyl groups located on the C2 and C9 positions in the constitution of selected quinoline or 1,10-phenanthroline derivatives, respectively, into vinyl (or styryl) products by applying Perkin condensation. The two groups of ligands differ in the presence of one or two arms. The structure of the molecule ((1*E*,1′*E*)-(1,10-phenanthroline-2,9-diyl)bis(ethene-2,1-diyl))bis(benzene-4,1,3-triyl) tetraacetate was determined by single-crystal X-ray diffraction measurements. The X-ray, NMR, and DFT computational studies indicate the influence of rotation (rotamers) on the physical properties of studied styryl molecules. The results show that the styryl molecules with the vinyl unit –CH=CH– exhibit significant static and dynamic hyperpolarizabilities. Quantum chemical calculations using density functional theory and B3LYP/6-311++G(d,p) with Grimme’s dispersion correction approach predict the existence and relative stability of different spatial *cis*(Z)- and *trans*(E)-conformers of styryl derivatives of quinoline and 1,10-phenanthroline, which exhibit different electronic distribution and conjugation within the molecular skeleton, dipole moments, and steric interactions, leading to variations in their photophysical behavior and various applications. Our studies indicate that the rotation and isomerization of aryl groups can significantly influence the electronic and optical properties of π-conjugated systems, such as vinyl units (–CH=CH–). The rotation of aryl groups around the single bond that connects them to the vinyl unit can lead to changes in the effective π-conjugation between the aryl group and the rest of the π-conjugated system. The rotation and isomerization of aryl groups in π-conjugated systems significantly impact their electronic and optical properties. These changes can modify the efficiency of π-conjugation, affecting charge transfer processes, absorption properties, light emission, and electrical conductivity. In designing optoelectronic materials, such as organic dyes, organic semiconductors, or electrochromic materials, controlling the rotation and isomerization of aryl groups can be crucial for optimizing their functionality.

## 1. Introduction

Quinoline and 1,10-phenanthroline derivatives represent highly versatile photoactive compounds that demonstrate distinct properties in coordination chemistry [1,2,3,4,5], medicinal chemistry [6,7,8,9,10,11,12,13,14,15], chemical engineering [16,17], electrochemistry [18,19], industrial chemistry [20,21], etc., [22,23]. Despite their importance, water-soluble ligands based on quinoline or 1,10-phenanthroline core are relatively poorly studied compounds. Developing efficient and convenient syntheses for such ligands could open new avenues for functional material and complex design [24,25].

Our studies describe novel and practical ways to introduce a polyphenol group under mild reaction conditions. In particular, we apply Perkin condensation to synthesize a vinyl (or styryl) analog of 1,10-phenanthroline and quinoline derivatives with a phenol function. This reaction also demonstrates a new, simple, and efficient strategy for converting methyl derivatives of 1,10-phenanthroline or quinoline into more reactive styryl derivatives [24]. We anticipate that the new way of converting methyl will find wide application in chemical synthesis.

The synthesized styryl derivatives of quinoline and 1,10-phenanthroline possess a specific π-conjugated structure, containing a vinyl that functions as a bridge between an azoquinoline or phenanthroline moiety and an additional phenyl ring. Such extended π-conjugation increases the stability of the molecule and ensures efficient π-delocalization throughout the whole structure.

Quantum chemical calculations using density functional theory (DFT) support the existence of multiple spatial *cis*(*Z*)- and *trans*(*E*)-conformers of these styryl derivatives of quinoline and 1,10-phenanthroline, which exhibit different electronic distribution and conjugation within the molecular skeleton, dipole moments, and steric interactions, leading to variations in their photophysical behavior. The presence of nitrogen atoms within the molecular system enables it to bind to specific ions selectively. As a result, styryl derivatives of quinolines and 1,10-phenanthrolines are utilized in chemical sensors for detecting heavy metal ions, such as copper and mercury, which is significant in environmental protection and chemical analysis [26,27,28,29,30].

For example, Aatif and Kumar designed a novel phenanthroline-benzothiazole fluorescent sensor (**L**) for detecting Hg^2+^ in both biological and environmental samples [27]. Fernandes et al. developed a self-contained, portable iron measurement system based on spectroscopic absorption using the phenanthroline complex for the detection of Fe^2+^ in water [31]. The device demonstrated high sensitivity (2.5 µg Fe^2+^/L) and a linear response in the 25–1000 µg Fe^2+^/L range [31]. Mula et al. reported a BODIPY-phenanthroline conjugate that allows highly selective, fluorescence turn-off detection of Cu^2+^ ions [32]. The sensor demonstrated 1:1 complex formation with Cu^2+^ and was successfully applied in live-cell imaging and serum sample analysis [32]. Riela et al. developed a hectorite/phenanthroline nanohybrid material as a selective and stable fluorescent sensor for Zn^2+^ detection in aqueous solution [33]. This material exhibited strong interaction with Zn^2+^ ions and was characterized by combined spectroscopic, microscopic, and theoretical techniques to confirm its sensing potential and structural stability [33]. Zhou et al. presented a dual-function fluorescence sensor based on the inner filter effect between NaYF_4_:Yb,Er@NaYF_4_@PAA and the Fe(II)-1,10-phenanthroline complex for the sensitive detection of Sn(II) and ascorbic acid (AA) [34]. The system exhibited excellent selectivity and linear response with detection limits of 1.08 μM for Sn(II) and 0.97 μM for AA, and was successfully applied to tap and spring water samples [34]. Soleymani et al. reported a dual-emission ratiometric fluorescent sensor based on terbium-1,10-phenanthroline–nitrogen-doped-graphene quantum dots for the sensitive detection of metformin in biological samples [35].

In this paper, we demonstrate how our newly synthesized water-soluble ligands align with these design principles and explore their photophysical and coordination properties in depth. In this context, a series of spectroscopic investigations were carried out to evaluate the response of the ligands toward various metal ions, focusing on changes in absorbance behavior and fluorescence intensity (quenching). Among the tested ions, Cu^2+^ induced the most significant spectral changes, highlighting the high specificity of the phenanthroline derivative.

## 2. Results and Discussion

### 2.1. Synthesis and Structural Characterization

**Scheme 1:**
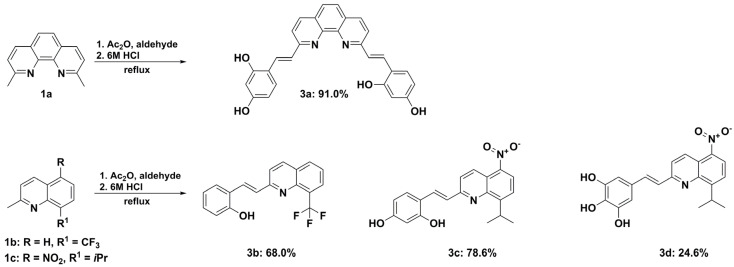
The synthesis of water-soluble 1,10-phenanthroline **3a** and quinolines **3b**, **3c**and **3d**. Aldehyde for **3b** 2-hydroxybenzaldehyde; **3c** 2,4-dihydroxybenzaldehyde; **3d** 3,4,5-trihydroxybenzaldehyde.

Recently, we reported the synthesis of a vinyl analog of 1,10-phenanthroline based on Perkin condensation [24]. In the present study, we successfully obtained a similar molecule 2,2′-((1*E*,1′*E*)-(1,10-phenanthroline-2,9-diyl)bis(ethene-2,1-diyl))diphenol, a vinyl analog of 1,10-phenanthroline, which, like our previously published molecule, has a phenolic group. This prompted us to obtain an analog with a polyphenol group, providing better solubility in water. For this purpose, we used a previously developed procedure in which we replaced 2-hydroxybenzaldehyde with 2,4-dihydroxybenzaldehyde. We received the product with 91% efficiency (Scheme 1). Since quinolines, like 1,10-phenanthrolines, are commonly used as ligands, we have expanded our research work to include this group of molecules. We chose the less commonly used, i.e., molecules 2-methyl-8-(trifluoromethyl)quinoline (**1b**) and 8-isopropyl-2-methyl-5-nitroquinoline (**1c**) as models. An analog with a trifluoromethyl group can be used as an NMR probe. The second model, on the other hand, refers to the isopropyl group of the first one, and the nitro group was chosen because, after reduction, the amino analogs will allow for further functionalization of the obtained compounds.

### 2.2. Single-Crystal X-Ray Data and Hirshfeld Surface Analysis

**Figure 1:**
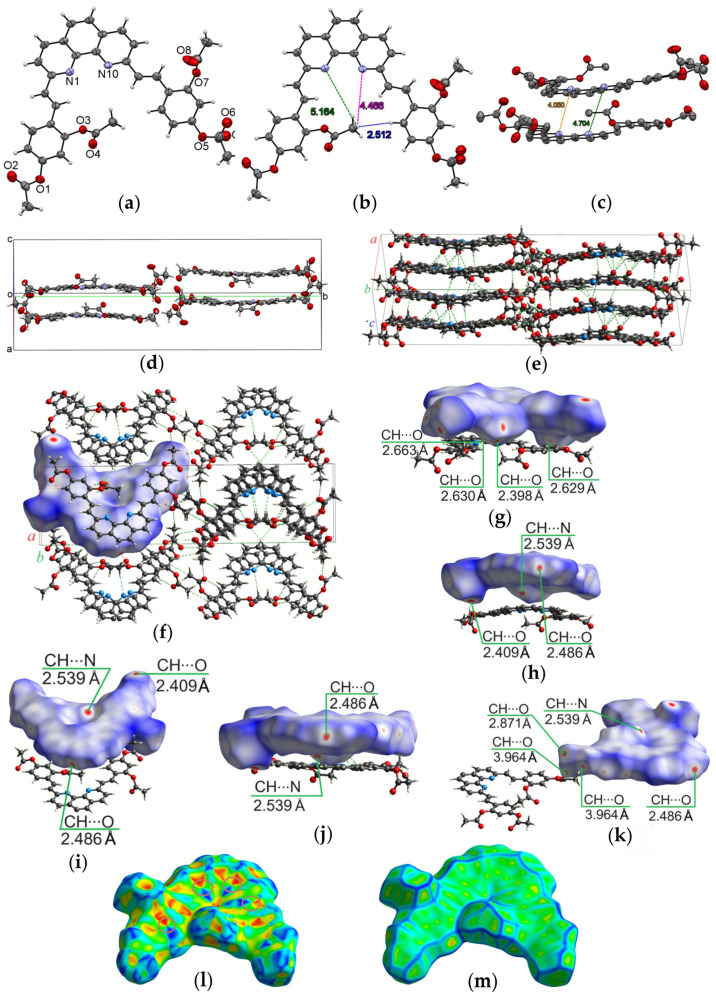
ORTEP (Oak Ridge thermal ellipsoid plot) drawings of ((1*E*,1′*E*)-(1,10-phenanthroline-2,9-diyl)bis(ethene-2,1-diyl))bis(benzene-4,1,3-triyl) tetraacetate (**2a**) with 50% probability (**a**), the selected intra- (**b**) and intermolecular distances (**c**), packing (**d**,**e**) and Hirschfeld *d_norm_* surface (**f**) of selected dimer configurations with indicated intermolecular distances in Å (**g**–**k**); shape index map (**l**) and curvedness map (**m**).

((1*E*,1′*E*)-(1,10-Phenanthroline-2,9-diyl)bis(ethene-2,1-diyl))bis(benzene-4,1,3-triyl) tetraacetate (**2a**)(Figure 1a) crystallizes in the monoclinic system, *P*2_1_/c, with unit cell dimensions of a = 9.5864(6) Å, b = 36.0174(19) Å, c = 8.6135(5) Å, cell volume V = 2973.01Å^3^, and Z = 4; the monoclinic angle (β) is 91.514(6), and the unit cell contains four molecules (Figure 1b–d). There is one molecule in the asymmetric part of the unit cell. The atom numbering of the selected atoms is shown in Figure 1a. The selected intra- and intermolecular interactions in **2a** presented in Figure 1b,c affect molecular conformation and crystal packing. The shortest intramolecular distance, 2.512 Å, belongs to the H···H contact, indicating very close proximity between the two hydrogen atoms in the **2a** molecule. The 5.164 Å and 4.466 Å distances correspond to long-range CH···N intramolecular contacts in **2a**. These contacts stabilize the molecular conformation and crystal packing, albeit at relatively weak interaction distances. The intermolecular N···N distances are 4.050 Å and 4.704 Å (Figure 1c).

Crystals of **2a** consist of closely spaced continuous columns of molecules (Figure 1e) linked together by numerous CH···O intermolecular interactions, which can be classified as weak hydrogen contacts according to the characteristics proposed by Desiraju and Steiner [36]. Each column consists of mutually parallel molecules with plane-to-plane distances between the centroids in parallel phenanthroline fragments of 3.954 Å and 4.007 Å. Although these distances are slightly larger than the optimal range for strong π···π interactions (3.3–3.8 Å), they still suggest weak π···π interactions due to the overlap of p-orbitals between the closely aligned phenanthroline cores in a face-to-face arrangement (Figure 1d). The detailed analysis of intermolecular interactions was performed with Hirshfeld d_norm_ surfaces of selected dimer configurations within the crystal of **2a** (Figure 1f). The red spots on d_norm_ indicate numerous CH···O and CH···N intermolecular interactions, which involve acetate groups and phenanthroline core (Figure 1g–k); these contacts contribute mainly to stability and well-ordered crystal packing motif of **2a**

The shape index map (Figure 1l) uses concave (red) and convex (blue) regions to represent areas where molecules fit into each other, similar to a lock-and-key principle. The presence of the red and blue triangles displays the areas where the π-system of one molecule intercalates or overlaps with another through van der Waals forces. It confirms the weak π···π stacking interactions between phenanthroline cores placed one above the other in neighboring molecules of **2a**.

The curvedness map in Figure 1m displays areas with different packing densities, where green regions represent flat molecular surfaces with low curvature and correspond to the π–π stacking regions in **2a**.

### 2.3. Structural Characterization of the Quinoline Derivatives **3b**, **3c**, and **3d**

**Figure 2:**
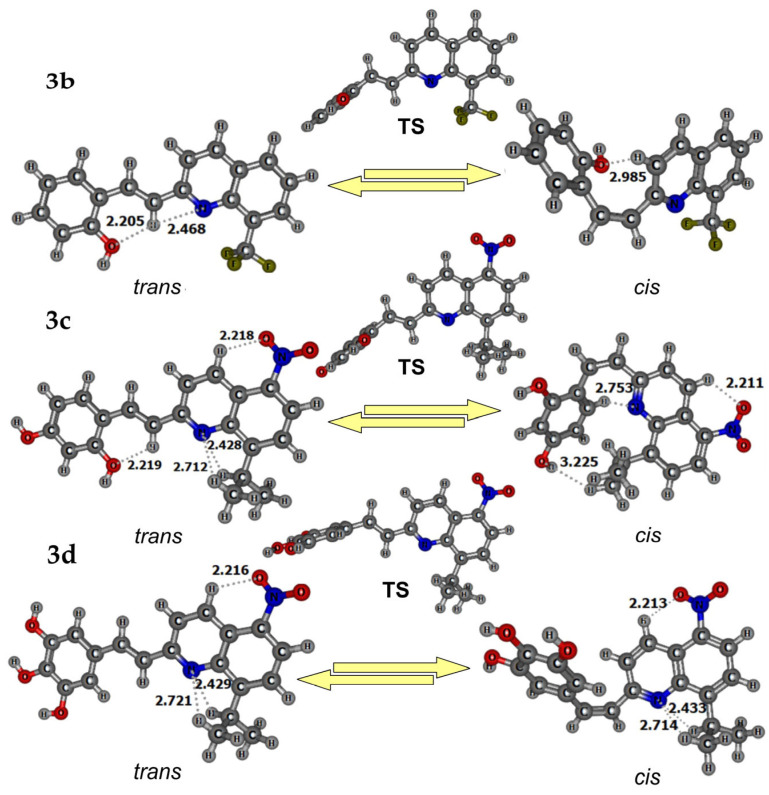
The DFT optimized structures using B3LYP/6-311++G(d,p) with Grimme’s dispersion correction approach of *trans*-, *cis*-isomers and TS structures of **3b**, **3c**, and **3d** with the intramolecular O···H, and N···H interactions (in Å) indicated.

**Figure 3:**
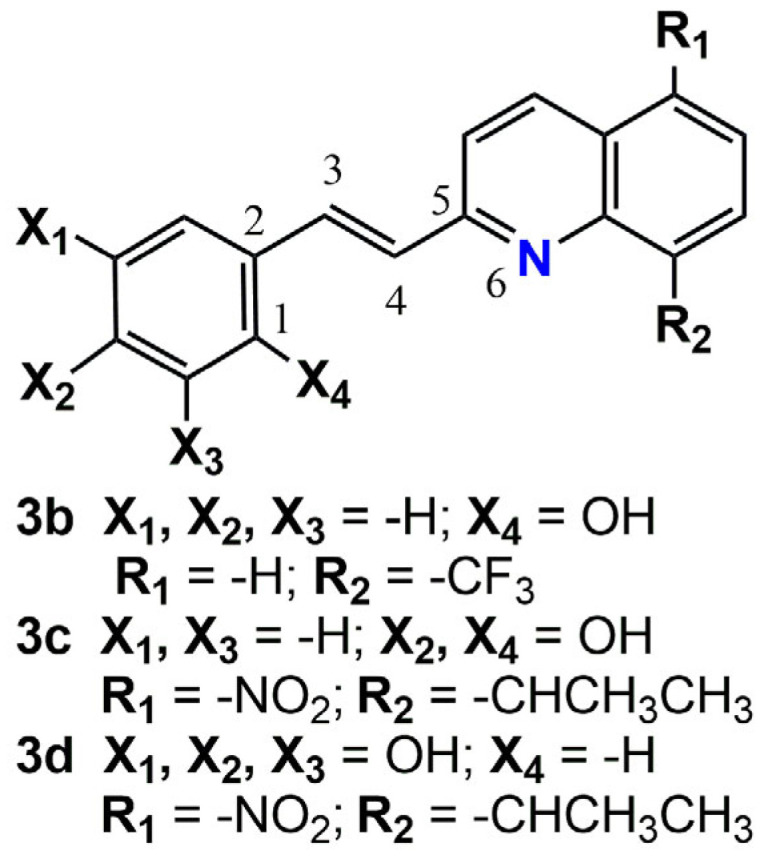
Atom numbering in **3b**, **3c**, and **3d**.

The B3LYP/6-311G++G(d,p), including Grimme’s dispersion correction optimized ground state structures of the *trans*-, *cis*-isomers, and the transition states (TS) with indicated intramolecular O···H, and N···H interactions for **3b**, **3c**, and **3d** are presented in Figure 2. The selected angles and energy barriers are summarized in Table 1 and Table 2. The structure of **3b**, **3c**, and **3d** *trans*-isomers is planar with dihedral angles D(C1C2C3C4) and D(C2C3C4C5) close to 0° and 180°, respectively (Figure 3, Table 1). Planarity facilitates π-conjugation of **3b**, **3c**, and **3d** structures, increasing their stability. The **3b**, **3c**, and **3d** *cis*-isomers exhibit significant geometrical distortions with non-planar D(C1C2C3C4) and D(C2C3C4C5) dihedral angle values (Table 1). The bond angles (A(C2C3C4), A(C3C4C5) vary slightly between the *trans*- and *cis*-isomers, but remain close to 120°, reflecting the sp^2^ hybridization of these carbon atoms.

The structure of the **3b** *trans*-isomer is stabilized by weak intramolecular O···H and N···H interactions with distances 2.205 Å and 2.468 Å (Figure 2). In the *cis*-isomer of the **3b**, the O···H distance increases to 2.985 Å, indicating a weaker interaction compared to the *trans* form. The **3c** and **3d** *trans-* and *cis*-isomers are also stabilized by intramolecular O···H and N···H interactions (Figure 2). All the compounds **3b**, **3c**, and **3d** possess structural trend that *trans* form exhibits relatively shorter and stronger O···H and N···H interactions compared to the corresponding *cis*-isomer. The energy differences (Δ*E*) between the *cis*- and *trans*-isomers indicate that the *trans*-isomers are energetically more stable, consistent with their planar and less strained structure. The energy differences relative to the *trans*-isomer are 3.81 kcal mol^−1^ for **3b**, 1.61 kcal mol^−1^ for **3c**, and 3.61 kcal mol^−1^ for **3d** (Table 1).

The TS structures of **3b**, **3c**, and **3d** exhibit distorted geometries with intermediate O···H and N···H distances, consistent with partial bond breaking/forming during the isomerization process. The *cis–trans* isomerization reaction proceeds primarily via an inversion pathway, as indicated by the significant increase in the dihedral angle D(C1C2C3C4) in the TS. As one can see from Table 1 and Table 2, the D(C1C2C3C4) changes from nearly 0° in the *trans-*isomers to around ±140° in the *cis*-isomers, and to ~83–86° in the TS. This structural feature supports the predominance of inversion over a pure rotation mechanism around the C=C bond. However, the rotation process plays a role in the changes in other torsional and bond angles (Table 1 and Table 2), which suggests that the process also involves a rotational component, consistent with a combined inversion-rotation mechanism.

The TS energy barriers for **3b**, **3c**, and **3d** vary significantly among the compounds; **3b** shows the highest energy barrier (13.56 kcal mol^−1^, Table 2), which reflects the higher stability of the *trans*-isomer. Energy barriers for **3c** and **3d** are much lower (4.72 kcal mol^−1^ and 5.43 kcal mol^−1^, respectively, Table 2) due to weaker intramolecular interactions and increased torsion in the TS. The lower energy barriers for **3c** and **3d** suggest a higher probability of *cis–trans* interconversion, leading to nonradiative decay pathways that quench photoluminescence.

### 2.4. Conformational Analysisof 3a

**Figure 4:**
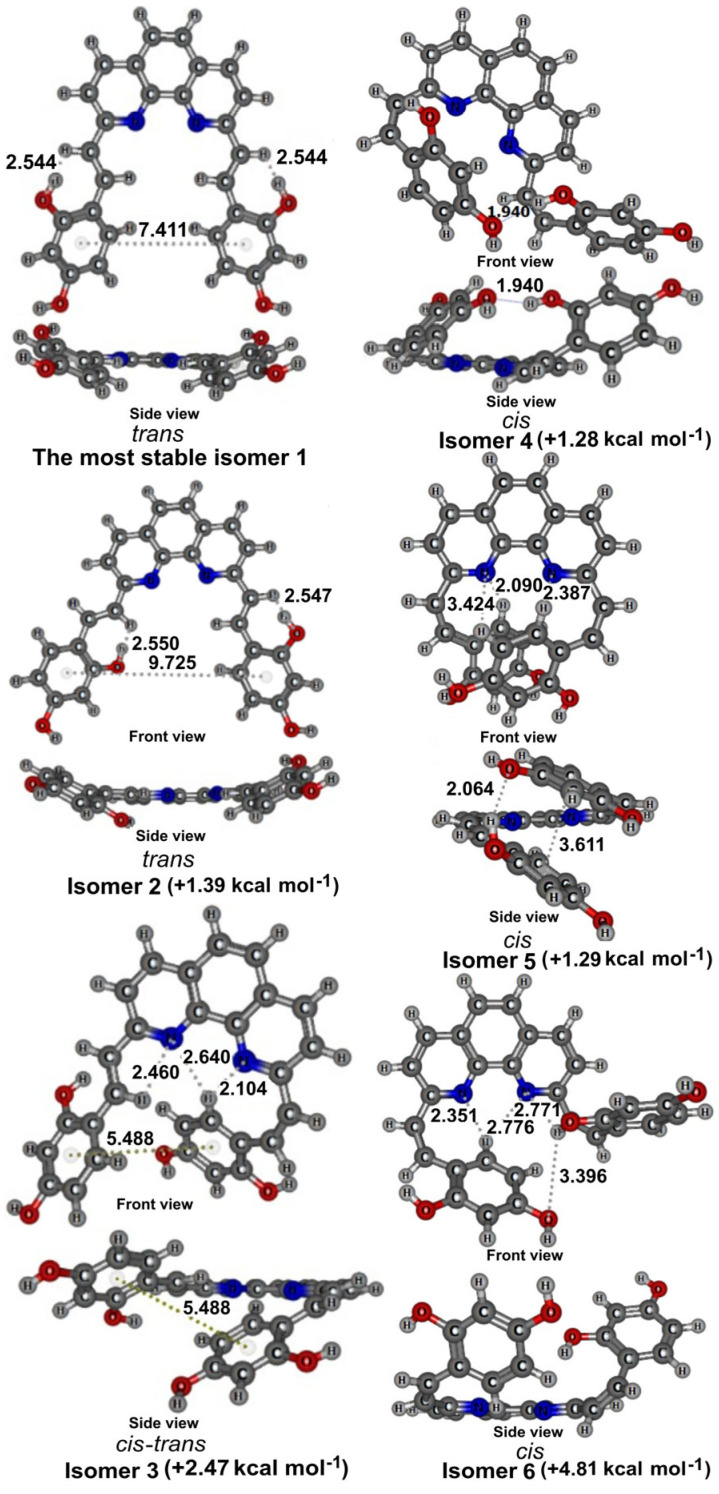
The DFT optimized structures of **3a** *trans*, *cis*, and *cis–trans* rotamers and their corresponding energy differences using B3LYP/6-311++G(d,p) with Grimme’s dispersion correction approach. The intramolecular O···H, and N···H interactions (in Å) are indicated.

**Figure 5:**
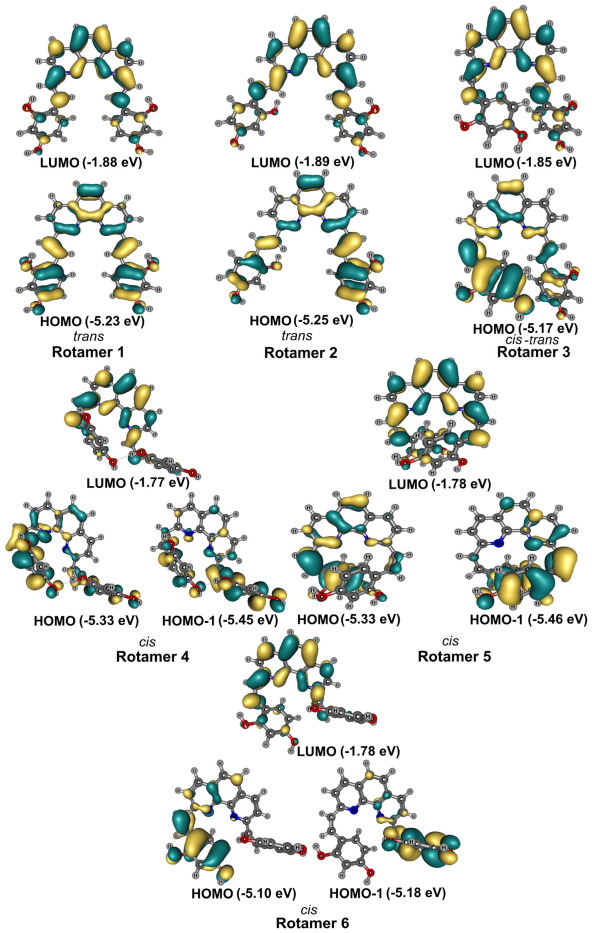
Molecular orbital nature of the first S_0_°→S₁ transition for **3a***trans*, *cis*, and *cis–trans*rotamers **1**–**6**. The S_0_°→S₁ transition has HOMO→LUMO nature for rotamers **1**–**3**, and is due to a combination of HOMO→LUMO and HOMO−1→LUMO for rotamers **4**–**6**. The orbital nature and the electronic transitions are presented in detail in Tables S1 and S2.

We simulated six structural *trans-*, *cis-*, and *cis–trans* rotamers **1**–**6** for **3a** (Figure 4 and Figure S2), and their highest occupied molecular orbital (HOMO) and lowest unoccupied molecular orbital (LUMO) are shown in Figure 5, and Table S1. The *trans* rotamer **1** of **3a** is energetically the most stable, due to minimized steric hindrance, and a centroid-to-centroid distance of 7.411 Å between the two terminal benzene rings with OH groups placed in opposite directions. The HOMO–LUMO gap for this rotamer **1** is 3.35eV.

The other two *cis* rotamers, **4** and **5** of **3a**, are only 1.28 and 1.29 kcal·mol^−1^ higher in energy than *trans* rotamer **1**. Rotamer **4**, with the unidirectional orientation of the terminal substituents, is stabilized by an intramolecular O···H interaction 1.940 Å, which appears between the OH groups on the terminal benzene rings. It exhibits a HOMO–LUMO gap of 3.56 eV which suggests a more localized electronic distribution and slightly reduced π-conjugation compared to *trans* rotamer **1** of **3a**. Rotamer **5**, with bidirectional branches, adopts a helical shape, with a centroid-to-centroid distance of 3.611 Å between the terminal benzene rings. This rotamer is stabilized by a network of intramolecular O···H and N···H interactions 2.064 Å, 2.090 Å, 2.387 Å, and 3.424 Å (Figure 4). Rotamer **5** has a HOMO–LUMO gap of 3.55 eV, similar to that of *cis* rotamer **4**, indicating the same π-delocalization principle.

*Trans* rotamer **2**, with unilaterally placed OH groups on the terminal benzene rings, lies higher by 1.39 kcal·mol^−1^ compared to *trans* rotamer **1**. While the HOMO–LUMO gap of rotamer **2** is only slightly larger (3.36 eV) than that of rotamer **1** (3.35 eV), this minor energy difference between the two rotamers originates primarily from subtle steric and conformational effects related to the orientation of the OH groups, rather than from differences in electronic delocalization, as evidenced by the similar shapes of the HOMO and LUMO shown in Figure 5.

*Cis-trans* rotamer **3** lies 2.47 kcal·mol^−1^ higher in energy than *trans* rotamer **1**; the structure adopts an intermediate conformation in which one terminal OH-substituted benzene ring is oriented in a *cis* fashion (toward the phenanthroline core), while the other is *trans*-oriented (away from the phenanthroline core). This mixed conformation leads to a more compact geometry than fully *trans* rotamers **1** and **2**. The HOMO–LUMO gap (3.32 eV) is narrower than that of rotamers **1** (3.35 eV) and **2** (3.36 eV), indicating slightly better π–π overlap in this intermediate geometry (Figure 5). The slightly higher HOMO energy (–5.17 eV), compared to rotamers **1** (−5.23 eV) and **2** (−5.25 eV), suggests reduced stabilization due to increased intramolecular steric strain, although its electronic properties are relatively similar.

*Cis* rotamer **6** is higher in energy than *trans* rotamer **1**, with a relative energy difference of 4.81 kcal·mol^−1^ (Figure 4). This rotamer adopts a fully *cis* conformation, where both OH-substituted terminal benzene rings are oriented toward the phenanthroline core. This geometry leads to distortion of the π-conjugated system, which reduces planarity and increases molecular strain. The HOMO–LUMO gap of rotamer **6** is 3.32 eV, the same as for *cis–trans* rotamer **3** (Figure 5). This suggests that despite the steric strain and non-planarity in rotamer **6**, the π–π* interactions remain moderately preserved, due to local orbital overlap between adjacent aromatic fragments. The HOMO energy level of rotamer **6** is higher (–5.10 eV) compared to the other rotamers **1**–**5** in Figure 5, which suggests reduced stabilization of the HOMO. This supports that intramolecular repulsion and conformational strain in rotamer **6** dominate electronic delocalization.

### 2.5. Spectroscopic Characterizationof the Phenanthroline Derivative 3a and of the Quinoline Derivatives 3c and 3d

**Figure 6:**
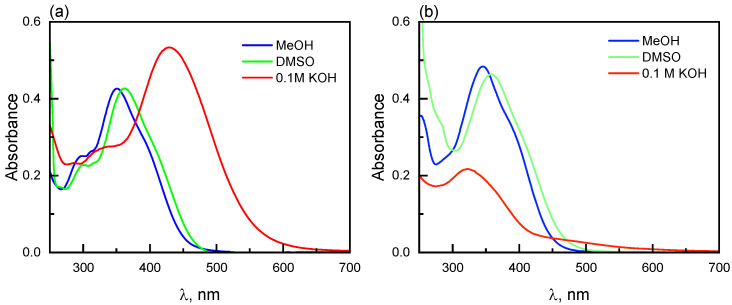
Absorption spectra of the compounds (**a**) **3c** and (**b**) **3d** at a concentration of 20 µM in different solvents.

**Figure 7:**
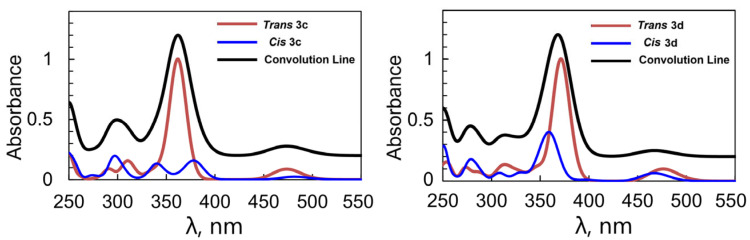
TheTDDFT/B3LYP/6-31G(d,p) simulated absorption spectra of the **3c** and **3d**, taking into account the DMSO solvent effects. The black line in absorption spectra corresponds to the convolution profile as a superposition of both *trans* and *cis* rotamer absorptions.

**Figure 8:**
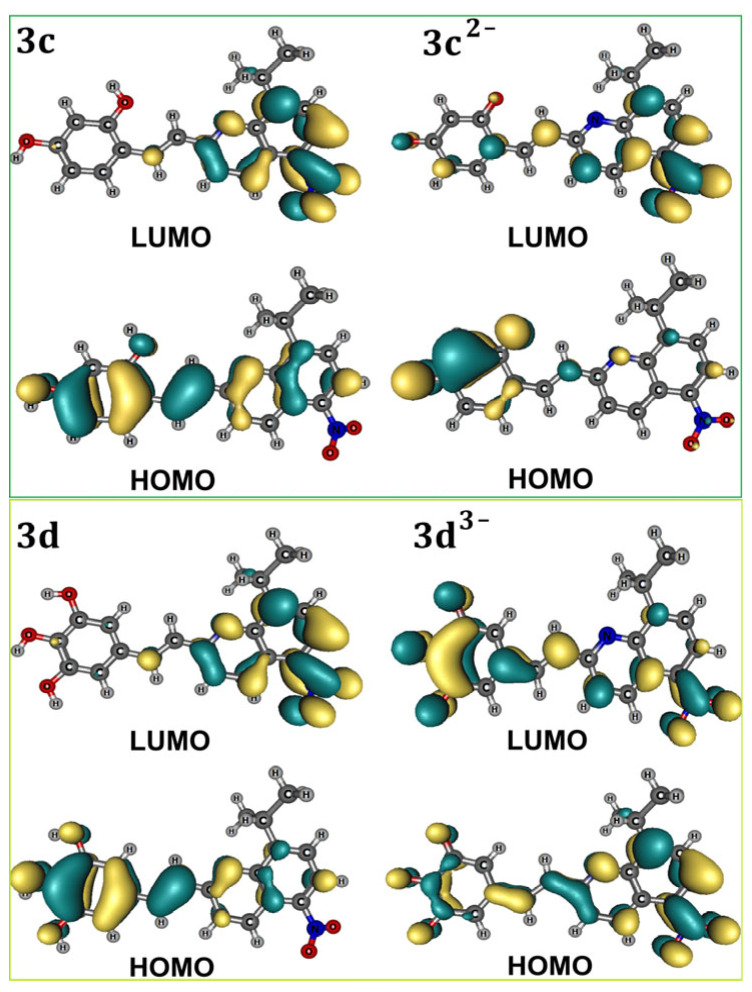
Frontier molecular orbitals of the *trans*-isomers of **3c** and **3d** in neutral form (**left**) and with the two and three ionized hydroxyl groups (**right**).

**Figure 9:**
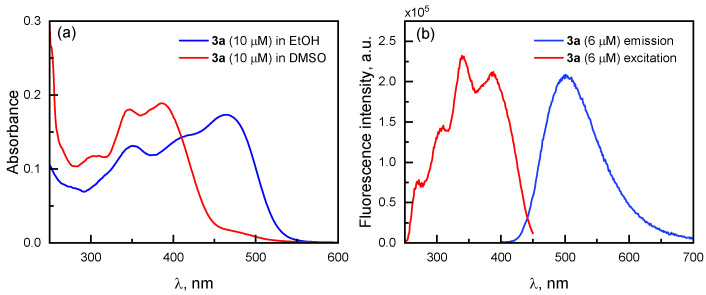
Optical characteristics of 1,10-phenanthroline (**3a**). (**a**) Absorption spectra and (**b**) excitation and emission spectra in DMSO under 387 nm excitation.

**Figure 10:**
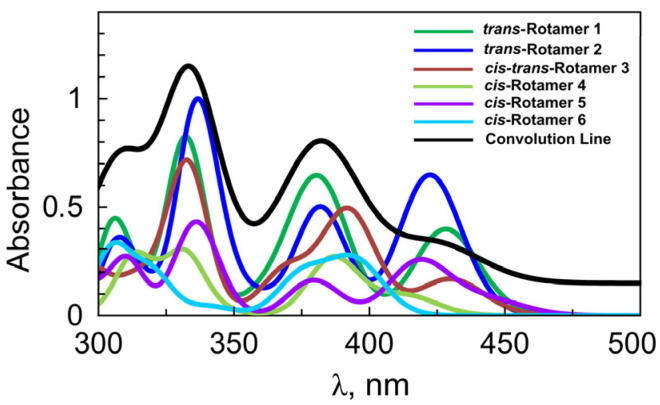
TDDFT simulated absorption spectra of the **3a,** taking into account the DMSO solvent effects. The black line in absorption spectra corresponds to the convolution profile as a superposition of all rotamer absorptions.

The derivatives 1,10-phenanthroline (**3a**) and quinolines (**3c** and **3d**) were spectroscopically characterized, and their photoluminescence properties, such as absorption maximum, molar extinction coefficient, excitation, emission, and luminescence decay time, were investigated. The data obtained are summarized in Table 3 and Tables S1–S3. In addition, the optimized geometries using different functionals, absorption, excitation, and emission spectra of the studied compounds **3a**, **3c**,and **3d** are presented in Figure 6, Figure 7, Figure 8, Figure 9 and Figure 10 and Figures S3–S5. The 1,10-phenanthroline derivative (**3a**) shows two absorption bands in ethanol (463 nm and 351 nm) and dimethylsulfoxide (DMSO) (387 nm and 346 nm) and exhibits fluorescence with an emission maximum in the 500–550 nm range (Table 3).

The solvent’s polarity significantly affects the spectroscopic properties of compound **3a**, with a bathochromic shift in the absorption and emission maxima observed as the solvent polarity decreases, indicating that the excited state of **3a** is stabilized more effectively in less polar solvents. In ethanol, **3a** shows the emission peak at 550 nm, resulting in a moderate Stokes shift of 87 nm. This indicates minimal structural reorganization between the ground singlet state (S₀) and the first excited singlet state (S₁), which is confirmed by quantum-chemical calculations (Figure S2); **3a** efficiently relaxes from the S₁ state to the S₀ state before emission. In DMSO, the emission peak shifts to 500 nm, reducing the emission wavelength and increasing the Stokes shift to 113 nm. This larger shift reflects solvent polarity effects, as DMSO stabilizes the excited state differently than ethanol. Depending on the solvent used, the fluorescence quantum yield (Φ_f_) of compound **3a** ranges from less than 1% to 8.17%. The obtained fluorescence intensity decays were fitted by biexponential and triexponential models for DMSO and EtOH, respectively (Figure S7). In ethanol, the excited-state lifetime of **3a** is 2.75 ns, consistent with weak fluorescence. In DMSO, the lifetime decreases to 0.99 ns despite the higher Φ_f_. This indicates faster decay pathways in DMSO due to a more stabilized S₁ state and enhanced radiative decay rates. The noticeable shifts in absorption and emission maxima for **3a** with changes in solvent polarity demonstrate its solvatochromic behavior, making it potentially useful for sensing applications.

The quinoline derivative (**3c**) has an absorption band with a near-ultraviolet maximum at 350 nm in methanol and 362 nm in DMSO, with slightly higher ε values than **3a**. The presence of a third hydroxyl group in the benzene ring of the quinoline derivative (**3d**) did not cause any significant changes in the absorption spectra in the presence of MeOH and DMSO (Figure 6).

The quantum-chemical time-dependent (TD) DFT/B3LYP/6-31G(d,p) calculations of the absorption spectra for the *trans* and *cis* rotamers of **3c** and **3d** in DMSO confirm the experimental observations, with absorption maxima in the near-UV region calculated at 364 nm for **3c** and 366 nm for **3d** in the convolution profiles (Figure 7). This band corresponds to the HOMO→LUMO+1 transition for both *trans* and *cis* forms of **3c** and **3d** (Tables S1 and S2). The first low-intensity S_0_→S_1_ transition is due to HOMO→LUMO configuration and was calculated at 474 nm (*f* =0.1051) for *trans***3c**, 482 nm (*f* = 0.0283) for *cis***3c**, 476 nm (*f* =0.1078) for *trans***3d**, 467 nm (*f* =0.0699) for *cis***3d** (Table S2).

The alkaline environment strongly affects the electronic charge distribution of **3c** and **3d**, as can be seen from the UV-Vis spectra (Figure 6), as the hydroxyl groups deprotonate to form phenolate anions. For the two hydroxyl groups in the structure of compound **3c**, a shift in the absorption maximum towards longer wavelengths is observed (Figure 6), since the two phenolate anions increase the charge delocalization in the aromatic system, thus reducing the energy difference between HOMO and LUMO. In contrast, in the case of three ionized hydroxyl groups, there is an excessive accumulation of negative charge, leading to a destabilization of the excited state and an associated increase in the difference between HOMO and LUMO. The effect is a shift in the absorption maximum towards shorter wavelengths (Figure 6).

The frontier molecular orbitals (FMO) of **3c** and **3d** and their ionized anionic forms **3c^2−^** and **3d^3−^** are presented in Figure 8. For the neutral **3c** and **3d**, the HOMO is mainly delocalized over the conjugated aromatic system with additional electron density extending to the hydroxyl groups. This reflects the π-conjugation across the whole system of **3c** and **3d** involving electron-donating hydroxyl groups. The LUMO is localized on the quinoline fragment and extends to the electron-withdrawing nitro group, which reflects its ability to accept electron density in electron-deficient (electrophilic) regions of the molecule (Figure 8). Such spatial separation between the HOMO (on electron-rich region) and LUMO (on electron-deficient region) suggests a pronounced intramolecular charge transfer (ICT) character in the excited state for neutral molecules of **3c** and **3d**.

After deprotonation of both hydroxyl groups in **3c**, the HOMO becomes significantly more localized, with electron density strongly concentrated on the negatively charged oxygen atoms. In contrast, the LUMO exhibits a broadened distribution across the molecular system of **3c^2^**, primarily extending over the electron-deficient nitro group, as well as the quinoline core and phenolate ring. This spatial extension of the LUMO reflects enhanced charge delocalization in the **3c^2−^** excited state. The increased separation of charge between the localized HOMO and delocalized LUMO reduces the HOMO-LUMO energy gap and improves the ICT. This decrease in excitation energy correlates directly with the experimentally observed red shift in the UV-visible absorption spectrum of **3c^2–^** compared to the neutral **3c**.

For the three ionized hydroxyl derivative of **3d**, the HOMO becomes more localized, and its electron density is asymmetric due to the excess negative charge from the deprotonated phenolate groups (Figure 8); this leads to destabilization of the HOMO energy level. The LUMO also undergoes a significant dual localization effect, being concentrated on the ionized hydroxyl group and the electron-withdrawing nitro group (Figure 8). Such spatial distribution, which is affected by the Coulomb repulsion and the altered electrostatic potential throughout the molecular system, leads to a more destabilized LUMO than the HOMO. This localization effect affects the orbital overlap and the nature of electronic transitions, contributing to the observed blue shift in the experimental absorption spectrum of **3d** in the alkaline environment.

Quinoline derivatives **3c** and **3d** with the nitro group do not exhibit photoluminescent properties that are typical for nitroaromatic compounds due to the nitro group effect, which introduces non-radiative decay mechanisms and distorts the electronic structure [37,38,39,40,41].

For a detailed analysis of the observed spectrum of **3a** in Figure 9, we performed TD DFT calculations of vertical electronic transitions simulated in DMSO for six rotamers **1**–**6**, which can coexist and affect the absorption and emission spectra of **3a**. The weak absorption observed experimentally in the 520–430 nm region in Figure 9a corresponds to the S_0_→S_1_ transitions calculated at 428 nm for *trans* rotamer **1** (oscillator strength, *f* = 0.3689), 422 nm for *trans* rotamer **2** (*f* = 0.5993), 433 nm for *cis–trans*rotamer **3** (*f* = 0.1561), 414 nm for *cis* rotamer **4** (*f* = 0.0838), 444 nm for *cis* rotamer **5** (*f* = 0.0637), and 395 nm for *cis* rotamer **6** (*f* = 0.2308). Among these, the *trans* rotamer **2** makes the largest contribution to this absorption. For rotamers **1**–**3**, the S_0_→S_1_ transition predominantly involves the HOMO→LUMO excitation, whereas for rotamers **4**–**6**, it involves a mixture of HOMO→LUMO and HOMO−1→LUMO transitions (Figure 5).

The observed absorption band at 387 nm in DMSO (Table 3, Figure 9a) is well reproduced by the TDDFT-calculated convolution line (black line in Figure 10) with a maximum at 383 nm. This band (383 nm) has a complex nature and corresponds to the absorption mainly from the *trans* rotamers **1** and **2**, with a minor contribution from the *cis–trans* rotamer 3, and the *cis* rotamers **4**–**6** (Figure 10). For the *trans* rotamers of **3a,** this absorption arises from the S_0_→S_2_ transition calculated at 385 nm for rotamer **1** and 382 nm rotamer **2** (Figure 10). The observed absorption band at 346 nm (Figure 9a, Table 3) corresponds to the calculated maximum at 335 nm in the convolution line (Figure 10). This band arises mainly from transition S_0_→S_4_ at 346 nm of *trans* rotamer **2** and transition S_0_→S_5_ at 332 nm of *trans* rotamer **1**, with *trans* rotamer **2** being the major contributor.

The fluorescence of **3a** corresponds to the S₁→S₀ transition from the LUMO to the HOMO and was calculated for rotamers **1**–**6**. The emission maxima were found at 527 nm (*f* = 0.5685) for *trans* rotamer **1**, 521 nm (*f* = 0.8771) for *trans* rotamer **2**, 568nm (*f* = 0.1271) for *cis–trans* rotamer **3**, 539nm (*f* = 0.1578) for *cis* rotamer **4**, 536nm (*f* = 0.2937) for *cis* rotamer **5**, 525nm (*f* = 0.5939) and for *cis* rotamer **6** (Table S3). These results confirm that all six rotamers **1**–**6** contribute to the emission profile, with *trans* rotamer **2** showing the most intense fluorescence due to the highest oscillator strength.

### 2.6. Spectroscopic Evaluation of Phenanthroline Derivative **3a** Towards Metal Ions

**Figure 11:**
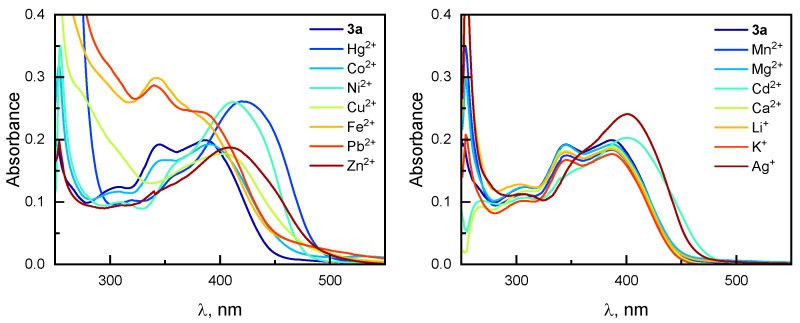
Absorption spectra of the compound **3a** (10 µM) in the presence of various ions Hg^2+^, Co^2+^, Ni^2+^, Ca^2+^, Cd^2+^, Mn^2+^, Mg^2+^, Zn^2+^, Li^+^, K^+^, Ag^+^(1 mM) and Cu^2+^, Fe^2+^, Pb^2+^ (0.1 mM).

The chemical reactivity of chemical probe **3a** towards different ions was measured in a DMSO: H_2_O (9:1, *v*/*v*) solution using colorimetric and fluorimetric methods. A number of changes were observed in the absorption spectra in the presence of test ions, as shown in Figure 11. Chemical probe **3a** shows characteristic absorption peaks at 345 and 386 nm, which are shifted towards longer wavelengths (bathochromic effect) and enhanced absorption intensity (hyperchromic effect) in the presence of Hg^2+^, Ni^2+^, and Ag^+^ ions. Only a bathochromic shift in the absorption maximum is observed for Cd^2+^ and Zn^2+^ ions. However, a shift in the maximum towards shorter wavelengths, combined with a hyperchromic effect, is observed for Fe^2+^ and Pb^2+^ ions. In contrast, Cu^2+^ ions show a bathochromic shift with a decrease in absorption intensity (hypochromic effect).

**Figure 12:**
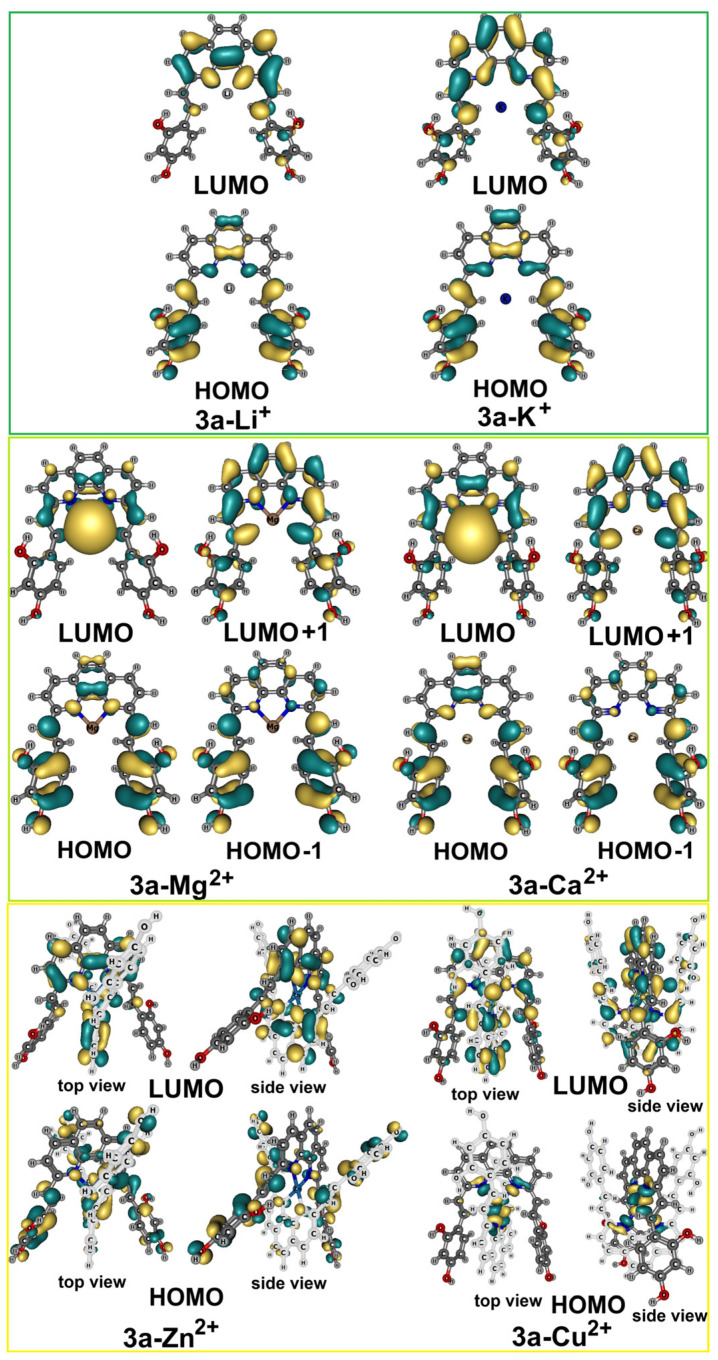
Frontier molecular orbitals for **3a-Li^+^**, **3a-K^+^**, **3a-Mg^2+^**, **3a-Ca^2+^**, **(3a)_2_-Zn^2+^**, and **(3a)_2_-Cu^2+^**. In the **(3a)_2_-Zn^2+^** and **(3a)_2_-Cu^2+^**complexes, one of the **3a** fragments is depicted in light gray for visual clarity.

The bathochromic shift in spectra of complexes of **3a** with Hg^2+^, Ni^2+^, Ag^+^, Cd^2+^, Zn^2+^, and Cu^2+^ is due to the stabilization of the LUMO energy level, which reduces the HOMO-LUMO energy gap and thus lowers the energy of electronic transitions. This shift is influenced by the electronic properties of the metal ions, including their charge density, coordination behavior, and ability to participate in π-bonds or accept electron density from a ligand. For example, as shown in Figure 11, the absorption spectra of **3a** and its complexes with Li^+^, K^+^, Mg^2+^, and Ca^2+^ ions are nearly identical, i.e., there are no significant shifts in band positions or changes in spectral shape. These experimental data are supported by TDDFT calculations using the B3LYP/6-31G(d,p) method performed for the most stable *trans*-isomer of **3a** with Li^+^, K^+^, Mg^2+^, and Ca^2+^ ions (Figure S6). For the complexes **3a-Li^+^** and **3a-K^+^**, the first absorption band, calculated in the 450–420 nm range, corresponds to the S_0_→S_1_ transition and arises from a single HOMO→LUMO excitation, similar to the free *trans*-isomer of **3a**. In contrast, for the divalent metal complexes **3a-Mg^2+^** and **3a-Ca^2+^**, the S_0_→S_1_ transition exhibits mixed character, arising predominantly from the HOMO→LUMO+1 with an additional HOMO−1→LUMO contribution (Figure 12). This orbital mixing reflects the influence of Mg^2+^ and Ca^2+^ coordination on the electronic structure of **3a**, through electrostatic interaction and polarization effects rather than direct involvement of the metal orbitals in the π-π* system.

Similar spectral profiles suggest that the coordination of these metal ions does not substantially change the electronic structure of **3a**, which indicates the absence of effective electronic interaction between the Li^+^ or K^+^ metal cations and the π-system of **3a** orweak complexation in the case of Mg^2+^ and Ca^2+^ cations. Such behavior is typical of electronically inert ions that form complexes without significant orbital overlap with the ligand (Figure 12). For the **3a-Li^+^**, **3a-K^+^**, **3a-Mg^2+^** and **3a-Ca^2+^** complexes, the HOMO is localized on the OH-substituted benzene fragments, vinyl groups, and particularly involves phenanthroline moiety. The LUMO for **3a-Li^+^** and **3a-K^+^** complexes and LUMO+1 for **3a-Mg^2+^** and **3a-Ca^2+^** complexes is concentrated on a phenanthroline core (Figure 12). The molecular orbitals (MOs) involved in the electronic transitions of **3a-Li^+^**, **3a-K^+^, 3a-Mg^2+^** and **3a-Ca^2+^** show a similar spatial distribution to those of the free *trans*-isomer of **3a** (Figure 5), which explains the similarity and minimal spectral shift observed in their absorption spectra (Figure 11).

**Scheme 2:**
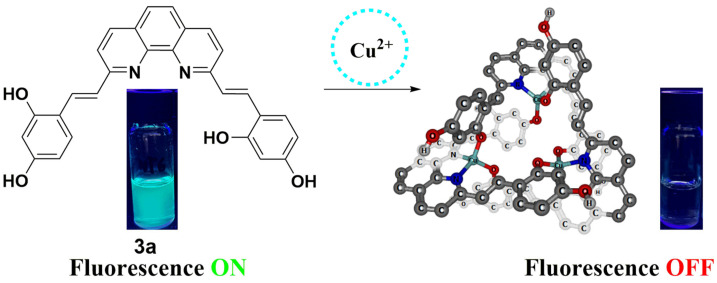
Proposed binding mode of **3a** with Cu^2+^. Hydrogen atoms near carbon in system of **3a** with **Cu^2+^** are omitted for clarity.

Transition metals are well known for their ability to form polynuclear complexes with chelating ligands such as phenanthroline. As suggested in Scheme 2, such coordination can lead to cage-like or extended structures. Although TDDFT calculations of absorption spectra of large polynuclear complexes are resource-intensive, we have simulated representative bidentate **(3a)_2_-Zn^2+^** and **(3a)_2_-Cu^2+^** complexes, where coordination involves the two nitrogen atoms from two phenanthroline units (Figure 12). These simplified systems serve to illustrate the influence of the transition metal center on the charge transport and electronic structure of the complex. For the **(3a)_2_-Zn^2+^** complex, the HOMO is localized on the OH-substituted benzene rings and vinyl groups, while the LUMO is concentrated on phenanthroline core, especially around the nitrogen atoms (Figure 12). In the case of the **(3a)_2_-Cu^2+^**, the chelation mode facilitates metal-to-ligand charge transfer (MLCT) and/or ligand-to-metal charge transfer (LMCT) interactions. These interactions lower the energy of π→π^*^ transitions, which contributes to the bathochromic shift in the spectrum of the **(3a)_2_-Cu^2+^** complex (Figure 11). Cu^2+^, being a paramagnetic d⁹ metal center, introduces significant spin–orbit coupling (SOC). This SOC perturbs the electronic structure and promotes non-radiative decay pathways, like intersystem crossing and vibrational relaxation. This results in both a reduction in absorption intensity (hypochromic effect) and effective fluorescence quenching, consistent with the role of Cu^2+^ as a strong fluorescence quencher [42].

**Figure 13:**
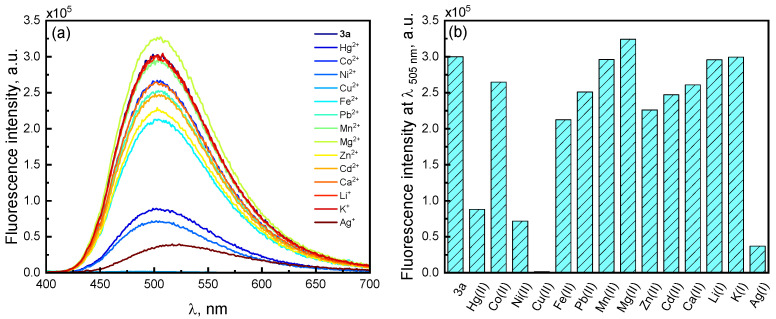
(**a**) Fluorescence spectra of the compound **3a** (10 µM) in the presence of various ions Hg^2+^, Co^2+^, Ni^2+^, Ca^2+^, Cd^2+^, Mn^2+^, Mg^2+^, Zn^2+^, Li^+^, K^+^, Ag^+^, Cu^2+^, Fe^2+^, Pb^2+^ (0.1 mM). (**b**) Bar graph of fluorescence intensity at 505 nm for derivative **3a** in the presence of various metal ions.

The fluorescence response of the derivative **3a** toward various metal ions was investigated in solution to evaluate its potential as a selective chemosensor. Figure 13a presents the emission spectra of **3a** in the absence and presence of different metal ions (100 μM), whereas the corresponding fluorescence intensities at the emission maximum are summarized in the bar chart (Figure 13b). Of all the tested cations, Cu^2+^ caused the most significant fluorescence quenching. This effect is attributed to strong coordination between Cu^2+^ and ligand **3a**, which may involve the nitrogen atoms of the phenanthroline core and the phenolic oxygen atoms. Notably, the Ag^+^ ion also induced significant quenching of fluorescence, although to a lesser extent than Cu^2+^. This may be attributed to Ag^+^ forming coordination interactions with the ligand via nitrogen and/or oxygen donor atoms. Therefore, the response to Ag^+^ should be considered in applications where silver contamination may be relevant. Other transition metal ions, such as Fe^2+^, Hg^2+^, and Ni^2+^, also reduced the emission intensity to a lesser extent, indicating a moderate binding affinity. In contrast, alkali and alkaline earth metals (e.g., K^+^, Ca^2+^, Mg^2+^, and Li^+^) had a negligible influence on fluorescence. These results confirm that compound **3a** exhibits high selectivity and sensitivity towards Cu^2+^, consistent with the Irving–Williams series [43], where Cu^2+^ typically forms the most stable complexes with first-row transition metal ions.

**Figure 14:**
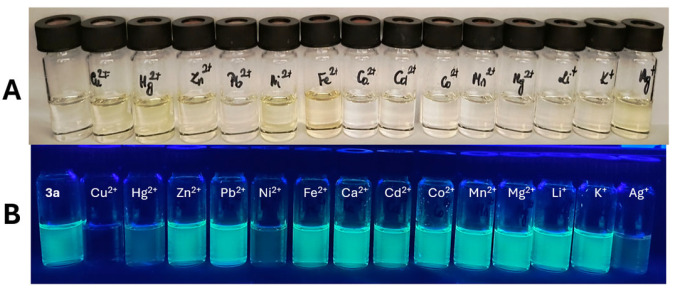
The photographic images under daylight mode (**A**) and UV light mode (**B**) of the compound **3a** with different metals.

In addition, the changes observed in the absorption and emission spectra of compound **3a** in the presence of various metal ions are visible to the naked eye (Figure 14A) and under UV illumination at 365 nm (Figure 14B). The results obtained suggest that compound **3a** can be used as a new tool for detecting copper ions.

In the presence of the analytes tested, no changes were observed in the absorption spectra of the quinoline **3c** and **3d** derivatives.

The limits of detection (LOD) and quantification (LOQ) for the selected metal ions (Cu^2+^, Ag^+^, Hg^2+^, and Ni^2+^) were determined based on changes observed in the emission spectra. LOD and LOQ were calculated using the following equations, with the standard deviation of the blank signal (σ) and the slope of the calibration curve (s) substituted in

LOD = 3.3 σ/s → → LOQ = 10 σ/s

The results are summarized in Table 4, and the corresponding calibration plots of fluorescence intensity versus metal ion concentration are presented in Figure S10. The LOD and LOQ values indicate that derivative **3a** exhibits the highest sensitivity toward Cu^2+^ and Ag^+^ ions, as evidenced by their significantly lower detection and quantification limits compared to Hg^2+^ and Ni^2+^. In particular, the low LOD values for Cu^2+^ (0.97 µM) and Ag^+^ (2.68 µM) suggest that derivative **3a** is well-suited for the detection of these metal ions at trace levels in solution. In contrast, the markedly higher LOD and LOQ values observed for Hg^2+^ (28.8 µM and 87.3 µM, respectively) and Ni^2+^ (29.8 µM and 90.4 µM) indicate a substantially lower sensitivity toward these ions.

Further studies focused exclusively on Cu^2+^ ions because they caused complete fluorescence quenching of derivative **3a** at the lowest concentration of all the tested metal analytes. Furthermore, Cu^2+^ ions yielded the lowest limits of detection (LOD) and quantification (LOQ), establishing them as the most promising analyte.

**Figure 15:**
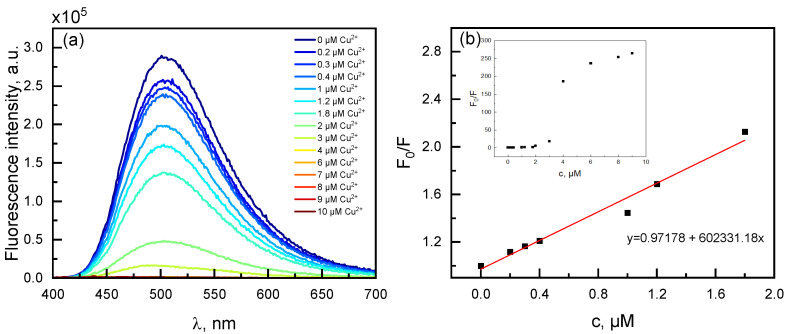
(**a**) Fluorescence spectra of the compound **3a** (10 µM) in the presence of various concentrations of Cu^2+^. (**b**) Stern–Volmer plots for **3a** in the presence of various concentrations of Cu^2+^.

To determine whether the mechanism of fluorescence quenching of derivative **3a** by Cu^2+^ ions is dynamic (collision) or static (complex formation), studies of the changes in fluorescence intensity of derivative **3a** towards Cu^2+^ ions at different concentrations were carried out (Figure 15a). A Stern–Volmer plot was made from the data obtained, which was linear in the concentration range 0.2 to 1.8 µM and was described by this equation:

F_0_/F = 1 + K_SV_ [Cu^2+^]

where F_0_ and F are the fluorescence intensities in the absence and presence of Cu^2+^, respectively, and [Cu^2+^] is the quencher concentration. From the slope of the linear region, the Stern–Volmer constant was determined to be K_SV_ = 6.0 × 10^11^ M^−1^ (Figure 15b).

At higher concentrations of Cu^2+^, a deviation from linearity was observed, with the plot exhibiting an exponential increase (see inset in Figure 15b), suggesting a change in the quenching mechanism. Such a deviation is characteristic of static quenching, likely caused by the formation of a ground-state non-fluorescent complex between **3a** and Cu^2+^. This hypothesis is further supported by changes in the absorption spectrum (Figure 11), which are commonly associated with complex formation in static quenching [44].

To further evaluate the nature of the quenching, the fluorescence lifetime (τ) of the **3a** derivative was considered. Using the Stern–Volmer constant and the known fluorescence lifetime of **3a** (τ = 0.99 ns), the bimolecular quenching rate constant k_q_ was calculated as follows:

k_q_ = K_SV_/τ = 6.06 × 10^20^ M^−1^s^−1^

This value greatly exceeds the diffusion controlled quenching rate in aqueous media (typically k_diff_ ≈ 10^9^–10^10^ M^−1^s^−1^), clearly ruling out a purely dynamic (collisional) quenching mechanism. Therefore, the quenching observed for the **3a**-Cu^2+^ system is best explained by a static quenching mechanism involving the formation of a non-emissive ground-state complex between **3a** and Cu^2+^. The formation of this complex is facilitated by photoinduced electron transfer (PET) or ligand-to-metal charge transfer (LMCT) processes, wherein electron density from the donor-rich ligand system of **3a** is transferred to the redox-active Cu^2+^ ion, effectively quenching fluorescence.

The modified logarithmic Stern–Volmer plot was used to calculate the complex formation constant between derivative **3a** and Cu^2+^ ions (Figure S7). The obtained value of the constant was 9.73 × 10^8^ M^−1^, indicating the high affinity of the chemosensor for copper ions and the stability of the formed complex.

The saturation method was used to determine the stoichiometry of the complex by analyzing the dependence of the fluorescence intensity on the Cu^2+^ concentration (Figure S8). The maximum fluorescence quenching was observed at a Cu^2+^ mole fraction of 0.4, which corresponds to a molar ratio of 1:2 (**3a**:Cu^2+^). This result suggests that one ligand **3a** binds two copper ions, leading to the formation of a complex with a stoichiometry of 1:2.

Based on the literature reports for polynuclear phenanthroline-metal complexes [45], we propose a cage-like coordination structure (Scheme 2), in which both the nitrogen atoms of the phenanthroline core and the hydroxyl oxygen atoms act as donor sites. The two Cu^2+^ ions are stabilized through bridging interactions, resulting in a rigid architecture that efficiently quenches fluorescence via static quenching.

## 3. Materials and Methods

### 3.1. Materials

All experiments were carried out in an atmosphere of dry argon, and flasks were flame-dried. Solvents were dried by usual methods (diphenyl ether, diethyl ether, and THF over benzophenone ketyl, CHCl_3_, and CH_2_Cl_2_ over P_4_O_10_, hexane, and pyridine over sodium-potassium alloy) and distilled. Chromatographic purification was carried out on silica gel 60 (0.15–0.3 mm, Macherey-Nagel GmbH & Co. KG, Dueren, Germany). 2,9-Dimethyl-1,10-phenanthroline (neocuproine), 2-hydroxybenzaldehyde, 2,4-dihydroxybenzaldehyde, and 3,4,5-trihydroxybenzaldehyde were purchased from Sigma–Aldrich (Poznań, Poland), and were used without further purification.

### 3.2. Instrumentation

NMR spectra were obtained with Avance 400 and 500 spectrometers (Bruker, Billerica, MA, USA) operating at 500.2 or 400.2 MHz (^1^H), 125.8 or 100.6 MHz (^13^C), and 470.5 MHz (^19^F) at 21 °C. Chemical shifts referenced to ext. TMS (tetramethylsilane) (^1^H, ^13^C), CFCl_3_ (^19^F) or using the residual CHCl_3_ signal (δ_H_ 7.26 ppm), and CDCl_3_ (δ_C_ 77.1 ppm) as internal references, and ext. DSS for ^1^H and ^13^C-NMR, respectively. Coupling constants are given in Hz. The LCMS-IT-TOF analysis was performed on an Agilent 1200 Series binary LC system coupled to a micrOTOF-Q system mass spectrometer (BrukerDaltonics, Bremen, Germany). High-resolution mass spectrometry (HRMS) measurements were performed using a Synapt G2-Si mass spectrometer (Waters, New Castle, DE, USA) equipped with an ESI source and quadrupole-time-of-flight mass analyzer. To ensure accurate mass measurements, data were collected in centroid mode, and mass was corrected during acquisition using leucine enkephalin solution as an external reference (Lock-Spray) (Waters, New Castle, DE, USA). The measurement results were processed using the MassLynx4.1 software (Waters, Milford, MA, USA) incorporated within the instrument. A Nicolet iS50 FTIR spectrometer was used to record spectra in the IR range of 4000–400 cm^−1^. FTIR spectra were recorded on a Perkin Elmer (Schwerzenbach, Switzerland) spectrophotometer in the spectral range of 4000–450 cm^−1^ with the samples in the form of KBr pellets. Elementary analysis was performed using a Vario EL III apparatus (Elementar, Langenselbold, Germany). Differential scanning calorimetry (DSC) measurements were performed using a Q2000 calorimeter (TA Instruments, New Castle, DE, USA) in a nitrogen stream at a scanning rate of 10 °C/min. Samples were analyzed in aluminum pans in the temperature range of 50 to 350 °C. Melting points were determined on an MPA100 OptiMelt melting point apparatus (Stanford Research Systems, Sunnyvale, CA, USA) and were uncorrected. Absorption spectra were recorded using a JASCO V-670 spectrophotometer (Jasco, Japan). The determination of the molar extinction coefficient was verified by linear least squares fitting of the values obtained from five independent solutions at different concentrations ranging from 2 µmol/L to 20 µmol/L. Fluorescence spectra were measured on an FLS-920 spectrofluorometer (Edinburgh Instruments, Livingston, UK) in a 10 mm quartz cuvette. The slit width was 1.0 nm. All samples for steady-state measurements were excited in optical dilution. Absorbance values at the selected excitation wavelengths were less than or equal to 0.1.

### 3.3. Photophysical Measurements

The stock solutionof chemosensor**3a** was prepared in DMSO at a concentration of 1 mM. All cation solutions were prepared in distilled water at a concentration of 10 mM. All spectroscopic experiments were carried out in DMSO:H_2_O (9:1, *v*/*v*). The concentration of probe **3a** was 10 µM in all spectroscopic studies. An excitation wavelength of 388 nm was used to record the fluorescence emission spectra. UV-Vis absorbance and fluorimetric experiments were performed on chemosensor**3a** in the presence of different ions. The concentrations were 1 mM for Hg^2+^, Co^2+^, Ni^2+^, Ca^2+^, Cd^2+^, Mn^2+^, Mg^2+^, Zn^2+^, Li^+^, K^+^, Ag^+^ and 0.1 mM for Cu^2+^, Fe^2+^, Pb^2+^. In addition, the optical properties of **3a** were investigated in the presence of 0 to 10 µM Cu^2+^ to evaluate the performance of the chemical sensor.

### 3.4. Photochemical Experiments

Fluorescence quantum yields of **3a** were calculated using quinine sulfate (in 0.1 M H_2_SO_4_ solution) as a standard (*Φ_ref_* = 0.54) [46] quantum yield was calculated accordingly to Equation (1), in which *Φ_ref_* is the absolute quantum yield of the reference, *η* is the refractive index of the solvent, and *Grad* is the gradient obtained from the plot of the integrated fluorescence intensity as a function of the absorbance. The subscripts s and ref refer to the sample and reference, respectively.

(1) $$\Phi_{f}=\Phi_{ref}\left(\frac{{Grad}_{s}}{Grad_{ref}}\right)\times \left(\frac{\eta_{s}^{2}}{\eta_{ref}^{2}}\right)$$

Fluorescence lifetime measurements were made using a time-correlated single photon counting system (TCSPC) with a pulsed picosecond LED (EPLED-380) as the excitation source. Collecting scattered light from a Ludox silica suspension measured the instrument response function. Fluorescence lifetimes were calculated from intensity decay analyses. Fluorescence decay was acquired at a level of 1 × 10^4^ counts at the peak and was fitted to a sum of exponentials:

(2) $$I(t)=\sum_{i=1}^{n}\alpha_{i}\exp(-t/\tau_{i})$$

with amplitudes, *α_i_*, and decay lifetimes *τ_i_*. Average lifetimes (*τ*) for multiexponential fluorescence decay were calculated from decay times and pre-exponential factors using the following:

(3) $$(\tau )=\frac{\sum \alpha_{i}\tau_{i}^{2}}{\sum \alpha_{i}\tau_{i}}$$

### 3.5. X-Ray Diffraction Experiments

The colorless crystals of **2a** were mounted on a SuperNova 4-circle X-ray diffractometer equipped with an Atlas CCD detector and used for data collection. X-ray intensity data were collected with mirror monochromated CuKα radiation (λ = 1.54184 Å) at a temperature of 150(1) K with ω scan mode.

Controlling the measurement procedure and data reduction were performed using the CrysAlis^Pro^ software Version 1.171.38.41q [47]. The same program was used to determine and refine the lattice parameters.

The structure was solved by direct methods and subsequently completed by the difference Fourier methods. All the non-hydrogen atoms were refined using a full-matrix, least-squares technique with anisotropic displacement parameters. The hydrogen atoms were treated as riding on their parent carbon atoms with isotropic displacement parameters (equal to 1.2 or 1.5 times the value of carbon displacement parameters). The SHELXS-2013 and SHELXL-2019/2 [48] programs were used for all the calculations.

One of the carbonyl oxygens from the carboxylic group is disordered over two positions in 0.796(6):0.204(6) ratio.

Details concerning crystal data and refinement are gathered in Table 1.

CCDC 2355007 contains the supplementary crystallographic data for this paper. These data can be obtained free of charge via www.ccdc.cam.ac.uk/structures (or from the CCDC, 12 Union Road, Cambridge CB2 1EZ, UK; Fax: +44 1223 336033; E-mail: deposit@ccdc.cam.ac.uk).

### 3.6. Quantum Chemical Computations

The molecular structures of the **3a**, **3b**, **3c**, and **3d** in the ground singlet state (S_0_) were optimized using density functional theory (DFT), and the B3LYP/6-311++G(d,p) [49,50,51] with Grimme’s D3 dispersion correction approach [52,53]. We have also tested the range-separated hybrid cam-B3LYP-GD3 functional with Grimme’s D3 dispersion correction, long-range corrected hybrid density functional wB97XD [54], which includes empirical dispersion corrections (D2), as well as the highly parametrized hybrid meta-GGA functional M06-2X [55], which incorporates 54% Hartree–Fock (HF) exchange with the 6-311++G(d,p) basis set (Figure S3). The minimal structural changes (0.010–0.015Å) found during testing of these functionals confirm that the B3LYP/6-311++G(d,p) level of theory is adequate to describe the key structural features of our systems.

Transition states (TS) for **3b**, **3c**, and **3d** were found using the QST3 [56] and Berny [57] optimization methods realized in Gaussian 16 [58]. The presence of one imaginary frequency in the TS indicates that the structure corresponds to a saddle point on the potential energy surface. This imaginary frequency represents the bond rotation of the C2-C3 and C3-C4 bonds responsible for the *cis–trans* isomerization process. For the TSs, intrinsic reaction coordinate (IRC) analysis was further performed to ensure that the obtained TS connects the minima. The TSs were calculated using the same B3LYP/6-311++G(d,p) [49,50,51] with Grimme’s D3 dispersion correction approach [52,53].

The first excited singlet (S_1_) state and the UV-visible absorption spectra of **3a**, **3c**, and **3d** were calculated using the time-dependent (TD) DFT formalism with the polarizable continuum model (PCM), employing TDDFT/B3LYP/6-31G(d,p) level of theory [59,60]. For the UV-visible absorption spectra of **3a**, **3c**, and **3d**, the lowest 20 vertical electronic transitions were computed. We tested the effect of diffuse functions on absorption spectra using the *trans*-isomer **3a** as an example. The absorption spectrum for the *trans* rotamer of **3a** was calculated with B3LYP functional using both the 6-31G(d,p) and 6- 31+G(d,p) basis sets (Figure S4). Both spectra have the same overall shape. Small shifts (~2–7 nm, red shift, Figure S4) were found, which are within the typical range of basis set effects when adding diffuse functions. This demonstrates that the diffuse functions only introduce minor corrections to the calculated electronic excitation energies. We have also calculated the optimized geometry of the *trans* rotamer of **3a**, both in the gas phase and with DMSO solvent effects included (Figure S5). The comparison shows only negligible changes in bond lengths (0.001Å, Figure S5), which supports the decision to perform geometry optimizations in the gas phase.

To analyze the intermolecular interactions in **2a**, Hirshfeld surfaces were calculated using Crystal Explorer 21.5 [61]. The procedure follows the approach detailed in Refs [62,63].

### 3.7. Synthesis of Vinyl Derivatives of 1,10-Phenanthroline (Neocuproine) 1a

Neocuproine **1a** (1.04 g, 5.0 mmol) was dissolved in Ac_2_O (100 mL), followed by the addition of 2,4-dihydroxybenzaldehyde (2.76 g, 20.0 mmol). The reaction was heated under reflux for 24 h. Then, the volatiles were removed in vacuo (16 mmHg). The crude product was purified by crystallization from a mixture of THF/Et_2_O and dried over P_4_O_10_ to yield precipitates as follows:

**((1*E*,1′*E*)-(1,10-phenanthroline-2,9-diyl)bis(ethene-2,1-diyl))bis(benzene-4,1,3-triyl) tetraacetate** (**2a**) beige; DSC m.p. = 188.54 °C; 3.0 g (4.8 mmol, 96%), ^1^H-NMR (DMSO-d6; 500.2 MHz) δ = 2.31 (s, 6H, CH_3_), 2.45 (s, 6H, CH_3_), 7.15 (d, ^3^*J*_H,H_ = 2.3 Hz, 2H, aromatic), 7.20 (dd, ^3^*J*_H,H_ = 8.5 Hz, ^4^*J*_H,H_ = 2.4 Hz, 2H, aromatic), 7.65 (d, ^3^*J*_H,H_ = 16.3 Hz, 2H, vinyl), 7.95 (s, 2H, aromatic), 7.95 (d, ^3^*J*_H,H_ = 16.3 Hz, 2H, vinyl), 8.06 (d, ^3^*J*_H,H_ = 8.7 Hz, 2H, aromatic), 8.12 (d, ^3^*J*_H,H_ = 8.4 Hz, 2H, aromatic), 8.48 (d, ^3^*J*_H,H_ = 8.3 Hz, 2H, aromatic); ^13^C{^1^H}-NMR (DMSO-d6; 125.8 MHz) δ = 20.8, 20.9, 117.1, 120.2, 121.7, 125.9, 126.2, 126.6, 127.2, 128.0, 131.2, 136.9, 145.2, 148.8, 150.8, 154.7, 168.9, 169.1; HRMS (ESI TOF): m/z Calcd for C_36_H_29_N_2_O_8_ (M + H)^+^ = 617.1924, Found 617.1921. CCDC 2355007.

**2a** (3.08 g, 5.0 mmol) was added to a water solution of hydrochloric acid (10%) and stirred under further reflux for 16 h. Next, a water solution of sodium hydroxide (10%) was added, and the red precipitate was filtered off and purified by crystallization from methanol and dried over P_4_O_10_ to yield precipitates as follows:

**4,4′-((1*E*,1′*E*)-(1,10-Phenanthroline-2,9-diyl)bis(ethene-2,1-diyl))bis(benzene-1,3-diol)** (**3a**) red 1.9 g (4.6 mmol, 91.0%), DSC m.p. = 264.26 °C; ^1^H-NMR (DMSO-d6/KOD; 500.2 MHz) δ = 6.31 (d, ^3^*J*_H,H_ = 8.4 Hz, 2H, aromatic), 6.36 (d, ^3^*J*_H,H_ = 2.4 Hz, 2H, aromatic), 7.42 (d, ^3^*J*_H,H_ = 16.4 Hz, 2H, vinyl), 7.50 (d, ^3^*J*_H,H_ = 8.4 Hz, 2H, aromatic), 7.78 (s, 2H, aromatic), 7.92 (d, ^3^*J*_H,H_ = 8.5 Hz, 2H, aromatic), 8.02 (d, ^3^*J*_H,H_ = 16.4 Hz, 2H, vinyl), 8.32 (d, ^3^*J*_H,H_ = 8.3 Hz, 2H, aromatic); ^13^C{^1^H}-NMR (DMSO-d6/KOD; 125.8 MHz) δ = 103.4, 108.2, 115.5, 121.1, 125.4, 126.1, 127.8, 129.3, 130.8, 137.3, 145.6, 157.2, 158.0, 160.0; HRMS (ESI TOF): m/z Calcd for C_28_H_21_N_2_O_4_ (M + H)^+^ = 449.1501, Found 449.1496.

### 3.8. Synthesis of Vinyl Derivatives of Quinoline

The synthesis of vinyl quinolines followed our procedure described in the literature [24,64,65]:

2-Methyl-8-(trifluoromethyl)quinoline (**1b**) or 8-isopropyl-2-methyl-5-nitroquinoline (**1c**) (10.0 mmol) was partially dissolved in Ac_2_O (100 mL), followed by the addition of 2-hydroxybenzaldehyde, 2,4-dihydroxybenzaldehyde or 3,4,5-trihydroxybenzaldehyde (20.0 mmol). The reaction was heated under reflux for 16 h. Then, the volatiles were evaporated from the resulting solution, and the residue was acidified by a water solution of hydrochloric acid (10%), and the reaction was stirred for 16 h, followed by the addition of CHCl_3_, and was filtered off. The crude product was dissolved in a water solution of NaOH (10%), and next was neutralized with an aqueous solution of hydrochloric acid (10%), and the solid was filtered. The crude product was purified by crystallization from methanol and dried over P_4_O_10_ to yield residues as follows:

**(*E*)-2-(2-(8-(Trifluoromethyl)quinolin-2-yl)vinyl)phenol** (**3b**) Yield: 2.14 g (6.8 mmol, 68.0%); DSC = 276.65 °C; ^1^H-NMR (DMSO–d6; 400.2 MHz) δ = 6.86 (td, ^3^*J*_H,H_ = 7.5 Hz, ^4^*J*_H,H_ = 1.2 Hz, 1H, aromatic), 7.00 (dd, ^3^*J*_H,H_ = 8.1 Hz, 4JH,H = 1.2 Hz, 1H, aromatic), 7.19 (ddd, ^3^*J*_H,H_ = 8.1 Hz, ^3^*J*_H,H_ = 7.2 Hz, ^4^*J*_H,H_ = 1.7 Hz, 1H, aromatic), 7.49 (d, ^3^*J*_H,H_ = 16.3 Hz, 1H, vinyl), 7.60 (t, ^3^*J*_H,H_ = 7.7 Hz, 1H, aromatic), 7.66 (d, ^3^*J*_H,H_ = 7.8 Hz, ^4^*J*_H,H_ = 1.6 Hz, 1H, aromatic), 7.89 (d, ^3^*J*_H,H_ = 8.6 Hz, 1H, aromatic), 8.10 (d, ^3^*J*_H,H_ = 7.4 Hz, 1H, aromatic), 8.16 (d, ^3^*J*_H,H_ = 7.6 Hz, 1H, aromatic), 8.17 (d, ^3^*J*_H,H_ = 16.1 Hz, 1H, vinyl), 8.41 (d, ^3^*J*_H,H_ = 8.7 Hz, 1H, aromatic), 10.12 (s, 1H, OH); ^13^C{^1^H}-NMR (DMSO–d6; 100.6 MHz) δ = 116.1, 119.5, 121.1, 122.9, 123.3, 124.6, 125.6 (q, ^1^*J*_C,F_ = 28.6 Hz CF_3_), 127.3, 127.7, 127.8, 128.3 (q, ^2^*J*_C,F_ = 5.5 Hz CF_3_), 130.1, 131.4, 132.8, 137.0, 144.0, 156.1, 157.0; ^19^F{^1^H}-NMR (DMSO–d6; 470.55 MHz) δ = −58.48; ^19^F-NMR (DMSO–d6; 470.55 MHz) δ = −58.48; LCMS-IT-TOF [M+H]^+^ = 316.0950 (100%), HRMS (IT TOF): m/z Calcd for C_18_H_13_F_3_NO [M+H]^+^: 316.0949, Found 316.0950.

**(*E*)-4-(2-(8-Isopropyl-5-nitroquinolin-2-yl)vinyl)benzene-1,3-diol** (**3c**) red Yield: 2.75 g (7.9 mmol, 78.6%); DSC = 213.12 °C; ^1^H-NMR (DMSO–d6; 500.2 MHz) δ = 1.37 (d, ^3^*J*_H,H_ = 6.9 Hz, 6H, 2CH_3_), 4.40 (hept, ^3^*J*_H,H_ = 6.9 Hz, 1H, CH), 6.70 (s, 2H, aromatic), 7.13 (d, ^3^*J*_H,H_ = 16.2 Hz, 1H, vinyl), 7.69 (d, ^3^*J*_H,H_ = 16.1 Hz, 1H, vinyl), 7.78 (d, ^3^*J*_H,H_ = 8.1 Hz, 1H, aromatic), 8.08 (d, ^3^*J*_H,H_ = 9.2 Hz, 1H, aromatic), 8.30 (d, ^3^*J*_H,H_ = 8.1 Hz, 1H, aromatic), 8.78 (d, ^3^*J*_H,H_ = 9.1 Hz, 1H, aromatic), 9.61 (bs, 1H, OH); ^13^C{^1^H}-NMR (DMSO–d6; 100.6 MHz) δ = 23.0, 27.6, 102.7, 107.7, 114.4, 118.8, 122.3, 123.4, 123.7, 124.3, 129.2, 132.0, 132.2, 143.4, 144.8, 153.9, 156.7, 157.7, 159.9; LCMS-IT-TOF [M+H]^+^ = 351.1340 (100%), HRMS (IT TOF): m/z Calcd for C_20_H_19_N_2_O_4_ [M+H]^+^: 351.1345, Found 351.1340.

**(*E*)-5-(2-(8-isopropyl-5-nitroquinolin-2-yl)vinyl)benzene-1,2,3-triol** (**3d**) black Yield: 0.9 g (24.6%); DSC = 213.12 °C; ^1^H-NMR (DMSO–d6; 500.2 MHz) δ = 1.38 (d, ^3^*J*_H,H_ = 7.0 Hz, 6H, 2CH_3_), 4.40 (hept, ^3^*J*_H,H_ = 6.9 Hz, 1H, CH), 6.69 (s, 2H, aromatic), 7.12 (d, ^3^*J*_H,H_ = 16.2 Hz, 1H, vinyl), 7.68 (d, ^3^*J*_H,H_ = 16.3 Hz, 1H, vinyl), 7.78 (d, ^3^*J*_H,H_ = 8.1 Hz, 1H, aromatic), 8.07 (d, ^3^*J*_H,H_ = 9.2 Hz, 1H, aromatic), 8.30 (d, ^3^*J*_H,H_ = 8.1 Hz, 1H, aromatic), 8.78 (d, ^3^*J*_H,H_ = 9.1 Hz, 1H, aromatic), 9.61 (bs, 1H, OH); ^13^C{^1^H}-NMR (DMSO–d6; 125.8 MHz) δ = 23.01, 27.7, 106.9, 118.9, 122.3, 123.6, 124.4, 124.5, 126.4, 132.1, 135.2, 136.9, 143.4, 144.8, 146.3, 154.0, 156.0; LCMS-IT-TOF [M-H]^−^ = 365.1142 (100%), HRMS (IT TOF): m/z Calcd for C_20_H_17_N_2_O_5_ [M-H]^−^: 365.1137, Found 365.1142.

## 4. Conclusions

Biological activity is believed to arise from the metal-chelating properties of the compounds toward various metal cations. This study tested the ability to chelate metal ions using selected water-soluble ligands based on the styryl derivatives of quinoline and 1,10-phenanthroline. These ligands were synthesized via standard Perkin condensation. Hydrophilic properties were introduced by incorporating phenol or polyphenol groups. The proton transfer reactions between the pyridine and phenol functional groups, or the generally simple protonation/deprotonation of pyridine rings in the 1,10-phenanthroline constitution, could be used as a pH indicator. The absorption spectra of compounds **3c** and **3d** at a concentration of 20 µM in various solvents confirmed that their spectral properties are influenced by basic media. For example, compound **3d** decomposed in CD_3_OD/KOD under aerobic conditions, due to oxidation.

The studied compounds were further characterized using analytical methods and rationalized based on quantum chemical DFT and TDDFT calculations using the B3LYP functional. The TDDFT/B3LYP/6-31G(d,p) calculations performed in DMSO confirm the experimental absorption and emission behavior of compounds **3a**, **3c**, and **3d**. For **3c** and **3d**, both *trans* and *cis* rotamers show absorption maxima in the near-UV region (364–366 nm), with the dominant transition being HOMO→LUMO+1. A weak, longer-wavelength absorption band corresponds to a low-intensity HOMO→LUMO transition (S_0_→S_1_). In the case of **3a**, the absorption and fluorescence properties are more complicated due to the presence of six rotamers. The observed weak absorption in the 520–430 nm region arises primarily from S_0_→S_1_ transitions in rotamers **1**–**3**, especially *trans* rotamer **2**, which shows the strongest oscillator strength (*f* = 0.5993). The emission of **3a** originates from the S_0_→S_1_ transition (LUMO→HOMO) and is contributed by all six rotamers. *Trans* rotamer **2** exhibits the most intense fluorescence (λ_em_ = 521 nm, *f* = 0.8771), making it the dominant species in the emission spectrum. Overall, the DFT and TDDFT calculations highlight the role of molecular conformation (rotamerism) in tuning the photophysical properties of **3a–3d** and explain the complex nature of their UV-Vis and fluorescence spectra. The strong correlation between computed and experimental results validates the use of TDDFT for designing and understanding such metal-sensing systems.

((1*E*,1′*E*)-(1,10-phenanthroline-2,9-diyl)bis(ethene-2,1-diyl))bis(benzene-4,1,3-triyl)tetraacetate (**2a**) was structurally characterized by the single-crystal X-ray diffraction method. The X-ray crystal structure analysis of **2a** showed the presence of the weak π···π stacking interactions between phenanthroline cores placed one above the other in neighboring molecules of **2a**. The ^1^H and ^13^C NMR spectra of the presented compounds displayed distinctively diagnostic signals from the vinyl (styryl) functional group. The analysis of the value of coupling constants for vinyl protons confirmed the sole presence of the *E* conformer.

In this work, we also developed a tandem UV-Vis and fluorescence spectroscopy method to detect metal ions. Compound **3a**, in DMSO:H_2_O (9:1, *v*/*v*), formed complexes with various metal cations, enabling their detection using colorimetric and fluorometric responses. This approach allowed sensitive detection of Hg^2+^, Ni^2+^, Cu^2+^, and Ag^+^ ions, with detection limits down to 1 mM, and 0.1 mM for Cu^2+^.

## Figures and Tables

**Table 1:** Selected structural parameters of *trans-* and *cis*-isomers of **3b**, **3c**, and **3d** and their corresponding energy differences Δ*E* (reported in kcal mol^−1^).

Compound	A(C2C3C4)	A(C3C4C5)	D(C1C2C3C4)	D(C2C3C4C5)	D(C3C4C5N6)	Δ*E*
**3b (*trans*)**	129.54	125.38	−0.002	−180.00	179.99	3.81
**3b (*cis*)**	130.54	129.84	−137.36	5.68	−157.61	
**3c (*trans*)**	129.74	125.29	0.32	−179.87	−178.35	1.61
**3c (*cis*)**	128.87	128.57	138.76	−8.83	−18.77	
**3d (*trans*)**	126.91	126.41	1.44	−179.78	−178.17	3.61
**3d (*cis*)**	129.07	129.21	143.64	−7.33	149.47	

The energy differences (Δ*E*) reported in Table 1 correspond to the B3LYP/6-311++G(d,p) with Grimme’s dispersion correction calculated energy differences relative to the most stable *trans*-isomer. Angles are reported in degrees.

**Table 2:** Selected structural parameters and energy barrier of the transition states (TS) of **3b**, **3c**, and **3d**.

TS	A(C2C3C4)	A(C3C4C5)	D(C1C2C3C4)	D(C2C3C4C5)	D(C3C4C5N6)	ΔE_ZPE_
**3b**	123.25	126.65	83.22	179.89	−175.09	13.56
**3c**	123.35	126.60	84.33	179.91	−175.91	4.72
**3d**	123.42	126.67	85.73	179.92	−176.12	5.43

The energy barrier of TSs (ΔE_ZPE_: TS—cis) is in kcal mol^−1^ (zero-point energies (ZPE) correction included). Angles are reported in degrees. Atom numbering corresponds to Figure 3.

**Table 3:** Spectroscopic characterization of **3a**, **3c,** and **3d** with different solutions.

	λ_abs_ [nm]	ε [M^−1^cm^−1^]	λ_em_[nm]	Stokes Shift [nm]	Φ_f_ [%]	τ [ns]
**3a**	463 ^a^ 351 ^a^	18,700 ^a^ 14,400 ^a^	550^a^	87 ^a^	<1 ^a^	2.75 ^a^
	387 ^c^ 346 ^c^	19,400 ^c^ 18,600 ^c^	500^c^	113 ^c^	8.17 ^c^	0.99 ^c^
**3c**	350 ^b^	21,400 ^b^	-	-	-	-
	362 ^c^	20,300 ^c^	-	-	-	-
	429 ^d^	27,400 ^d^	-	-	-	-
**3d**	346 ^b^	25,900 ^b^	-	-	-	-
	359 ^c^	23,800 ^c^	-	-	-	-
	323 ^d^	118,00 ^d^	-	-	-	-

^a^ EtOH, ^b^ MeOH, ^c^ DMSO, ^d^ 0.1 M KOH.

**Table 4:** The limit of detection and quantification for derivative **3a** with various metal ions.

Metal Ion	LOD [µM]	LOQ [µM]
Cu^2+^	0.97	2.96
Ag^+^	2.68	8.12
Hg^2+^	28.8	87.3
Ni^2+^	29.8	90.4

## Data Availability

The data set presented in this study is available in this article.

## Supplementary Materials

The following supporting information can be downloaded at https://www.mdpi.com/article/10.3390/molecules30122659/s1, Part I. Quantum chemical data, Figure S1: The optimized molecular structure and bond lengths (Å) of trans and cis rotamers of **3b**, **3c**, and **3d** in the ground singlet state; Figure S2: The optimized molecular structure and bond lengths (Å) of spatial trans, cis, and *cis*-*trans* rotamers of **3a** in the ground singlet S_0_ state and the first excited S_1_ state; Figure S3: The optimized geometry of the trans rotamer of **3a** with different functionals B3LYP-GD3, cam-B3LYP-GD3, wB97XD, and M06-2X; Figure S4: TDDFT calculated absorption spectra of the trans rotamer of **3a** calculated with B3LYP functional using both 6-31G(d,p) and 6-31+G(d,p) basis sets; Figure S5: The B3LYP-GD3/6-311++g(d,p) optimized geometry of the trans rotamer of **3a** in the gas phase and with the dimethylsulfoxide solvent effects included; Figure S6: TDDFT calculated absorption spectra of the complexes of the trans rotamer of **3a** with Li^+^, K^+^, Mg^2^⁺, and Ca^2^⁺ ions calculated with B3LYP/6-31G(d,p) method; Table S1: Molecular orbitals and corresponding energy levels for **3a** (rotamers 1–6), **3c**, and **3d** are calculated at the DFT/B3LYP/6-31G(d,p) level of theory; Table S2: Wavelengths (λ), oscillator strengths (*f*), and orbital assignment of the selected electronic transitions in the absorption spectra of the **3a** rotamers 1–6, *trans* and *cis* **3c**, and **3d** calculated at the TDDFT/B3LYP/6-31G(d,p) level of theory; Table S3: Spectroscopic data of the S_1_→S_0_ transitions for the **3a** (rotamers 1–6) calculated at the TDDFT/B3LYP/6-31G(d,p) level. Part II. Experimental data, Figure S7: The fluorescence decay profiles of compound **3a** in different solvents with excitation at 376.2 nm; Figure S8: Modified logarithmic-type Stern-Volmer plot for **3a** in the presence of various concentrations of Cu^2+^; Figure S9: Job’s plots for **3a** in the presence of various concentrations of Cu^2+^; Figure S10: The calibration curves of fluorescence intensity at 505 nm as a function of metal ion concentration for derivative **3a** (a) Cu^2+^, (b) Ag^+^, (c) Hg^2+^, (d) Ni^2+^ and NMR experimental data including ^1^H, ^13^C{^1^H}, ^19^F, ^19^F{^1^H} NMR, MS, and HRMS spectra of the compounds.

## Abbreviations

B3LYP	Becke, 3-parameter, Lee–Yang–Parr hybrid functional
DFT	density-functional theory
DMSO	dimethyl sulfoxide
DSS (NMR standard)	sodium trimethylsilylpropanesulfonate
HOMO	highest occupied molecular orbital
LUMO	lowest occupied molecular orbital
MS	mass spectrometry
ESI	electrospray ionization
HF	Hartree–Fock
HRMS	high-resolution mass spectrometry
THF	tetrahydrofuran
TS	transition state
SOC	spin–orbit coupling
