# Supplementary material for: New Tools in Heavy Metal Detection: Synthesis, Spectroscopic, and Quantum Chemical Characterization of Selected Water-Soluble Styryl Derivatives of Quinoline and 1,10-Phenanthroline
Source: Molecules. 2025 Jun 19;30(12):2659. doi: 10.3390/molecules30122659 (PMC12195994; doi:10.3390/molecules30122659)
Supplement: Supplementary file 1 [file molecules-30-02659-s001.zip › molecules-3666732-supplementary.pdf]

Supporting Information for

New Tools in Heavy Metal Detection: Synthesis, Spectroscopic and Quantum Chemical Characterization of Selected Water-Soluble Styryl Derivatives of Quinoline and 1,10-Phenanthroline

Jacek E. Nycz [1,*], Jolanta Kolińska [2], Nataliya Karaush-Karmazin [3,*], Tieqiao Chen [4,*], Maria Książek [5] and Joachim Kusz [5]

[1] Institute of Chemistry, Faculty of Science and Technology, University of Silesia in Katowice, ul. Szkolna 9; 40-006 Katowice, Poland; jacek.nycz@us.edu.pl (JN)
[2] Institute of Polymer and Dye Technology, Faculty of Chemistry, Lodz University of Technology, Stefanowskiego 12/16, 90-924 Lodz, Poland; jolanta.kolinska@p.lodz.pl (JK)
[3] Department of Chemistry and Nanomaterials Science, Bohdan Khmelnytsky National University, 18031 Cherkasy, Ukraine; karaush22@ukr.net (NKK)
[4] Ministry of Education Key Laboratory of Advanced Materials for Tropical Island Resources, Hainan Provincial Key Laboratory of Fine Chem, Hainan Provincial Fine Chemical Engineering Research Center, Hainan University, Haikou 570228, China; chentieqiao@hnu.edu.cn (TC)
[5] Institute of Physics, Faculty of Science and Technology, University of Silesia in Katowice, 75 Pułku Piechoty 1a, 41-500 Chorzów, Poland; maria.ksiazek@us.edu.pl (MK), joachim.kusz@us.edu.pl (JK)
* Author to whom correspondence should be addressed

Table of Contents

Part I. Quantum chemical data

Figure S1. The optimized molecular structure and bond lengths (Å) of *trans* and *cis* rotamers of **3b**, **3c**, and **3d** in the ground singlet state.

Figure S2. The optimized molecular structure and bond lengths (Å) of spatial *trans*, *cis*, and *cis-trans* rotamers of **3a** in the ground singlet S_0 state and the first excited S_1 state.

Figure S3. The optimized geometry of the *trans* rotamer of **3a** with different functionals B3LYP-GD3, cam-B3LYP-GD3, wB97XD, and M06-2X.

Figure S4. TDDFT calculated absorption spectra of the *trans* rotamer of **3a** calculated with B3LYP functional using both 6-31G(d,p) and 6-31+G(d,p) basis sets.

Figure S5. The B3LYP-GD3/6-311++g(d,p) optimized geometry of the *trans* rotamer of **3a** in the gas phase and with the dimethylsulfoxide solvent effects included.

Figure S6. TDDFT calculated absorption spectra of the complexes of the *trans* rotamer of **3a** with Li^+, K^+, Mg^{2+}, and Ca^{2+} ions calculated with B3LYP/6-31G(d,p) method.

Table S1. Molecular orbitals and corresponding energy levels for **3a** (rotamers **1–6**), **3c**, and **3d** are calculated at the DFT/B3LYP/6-31G(d,p) level of theory.

Table S2. Wavelengths (λ), oscillator strengths (*f*), and orbital assignment of the selected electronic transitions in the absorption spectra of the **3a** rotamers **1–6,** *trans* and *cis* **3c,** and **3d** calculated at the TDDFT/B3LYP/6-31G(d,p) level of theory.

Table S3. Spectroscopic data of the $S_1 \rightarrow S_0$ transitions for the **3a** (rotamers **1–6**) calculated at the TDDFT/B3LYP/6-31G(d,p) level.

Part II. Experimental data

Figure S7. The fluorescence decay profiles of compound **3a** in different solvents with excitation at 376.2 nm.

Figure S8. Modified logarithmic-type Stern-Volmer plot for **3a** in the presence of various concentrations of Cu^{2+}.

Figure S9. Job's plots for **3a** in the presence of various concentrations of Cu^{2+}.

Figure S10. The calibration curves of fluorescence intensity at 505 nm as a function of metal ion concentration for derivative **3a** (a) Cu^{2+}, (b) Ag^{+}, (c) Hg^{2+}, (d) Ni^{2+}.

Figures S11a-d. ^{1}H, $^{13}C\{^{1}H\}$ NMR; MS and HRMS spectra of the **2a**.

Figures S12a-d. ^{1}H, $^{13}C\{^{1}H\}$ NMR; MS and HRMS spectra of the **3a**.

Figures S13a-f. ^{1}H, $^{13}C\{^{1}H\}$, $^{19}F\{^{1}H\}$ NMR; MS and HRMS spectra of the **3b**.

Figures S14a-d. ^{1}H, $^{13}C\{^{1}H\}$ NMR; MS and HRMS spectra of the **3c**.

Figures S15a-d. ^{1}H, $^{13}C\{^{1}H\}$ NMR; MS and HRMS spectra of the **3d**.

Table S4. Crystal data and structure refinement details of compound **2a**.

Optimized Cartesian coordinates for the rotamers **1–6** of **3a** (Tables **S5–S10**)

Quantum chemical data

<table>
<tr><td>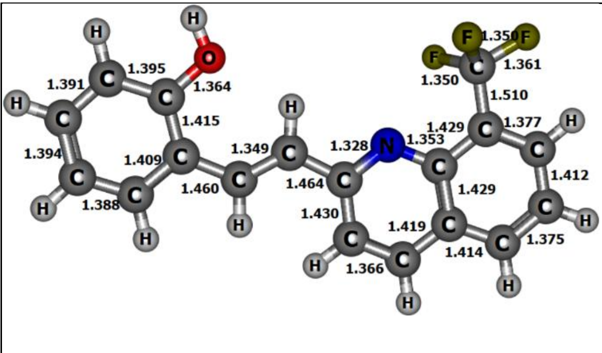</td><td>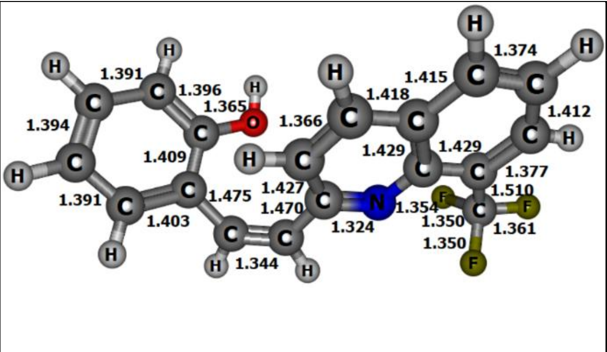</td></tr>
<tr><td>trans</td><td>cis</td></tr>
<tr><td colspan="2">3b</td></tr>
<tr><td>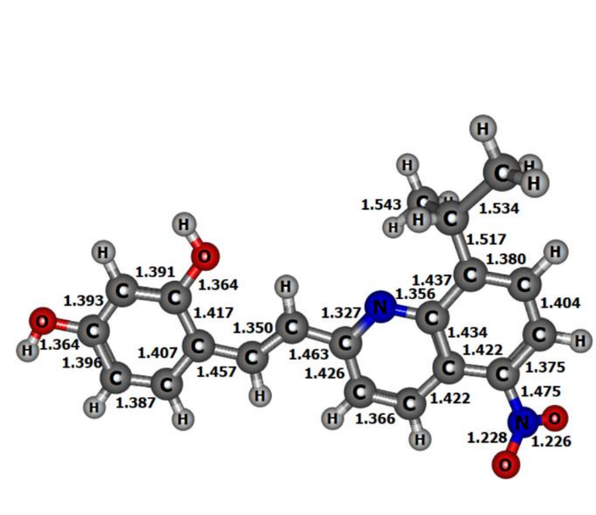</td><td>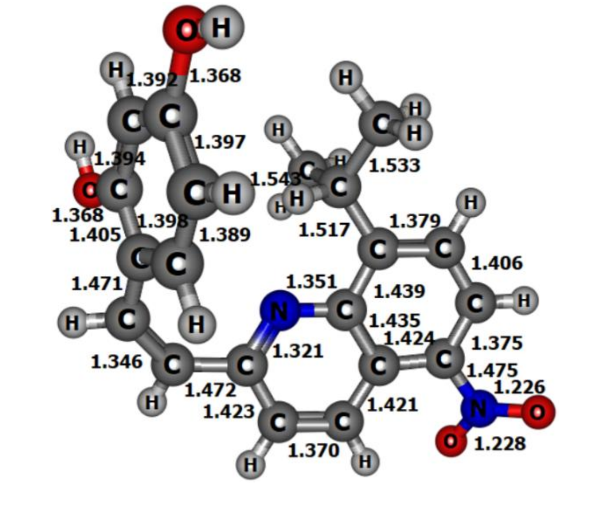</td></tr>
<tr><td>trans</td><td>cis</td></tr>
<tr><td colspan="2">3c</td></tr>
<tr><td>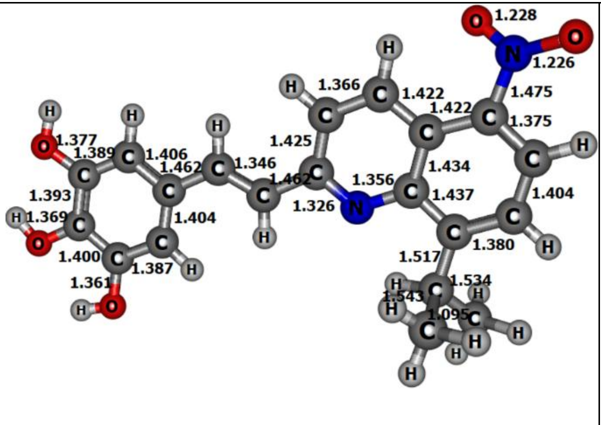</td><td>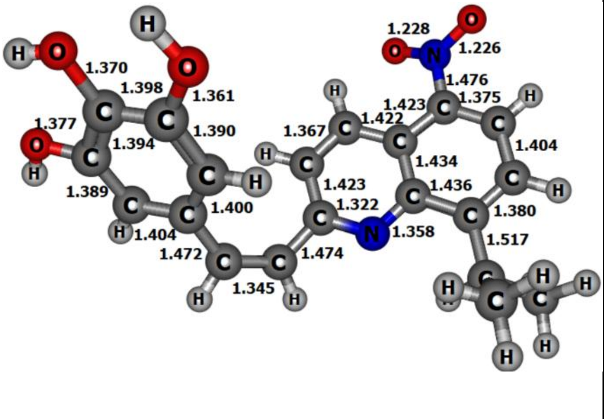</td></tr>
<tr><td>trans</td><td>cis</td></tr>
<tr><td colspan="2">3d</td></tr>
</table>

Figure S1. The optimized molecular structure and bond lengths (Å) of *trans* and *cis* rotamers of **3b**, **3c**, and **3d** in the ground singlet state calculated at the DFT/B3LYP/6-311++g(d,p) with Grimme's dispersion correction approach

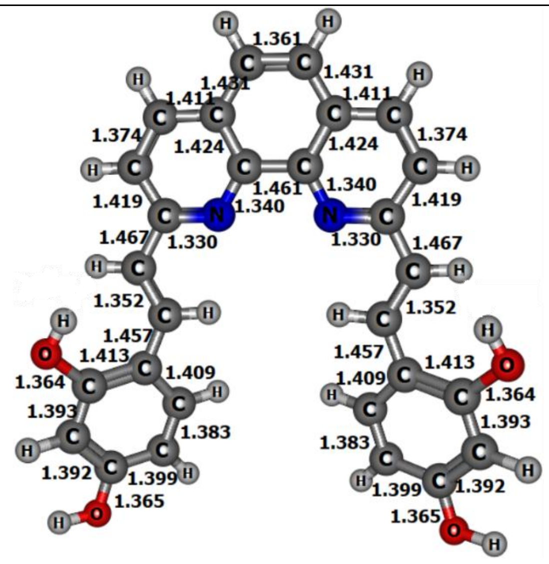

trans
Isomer 1 (S_0 state)

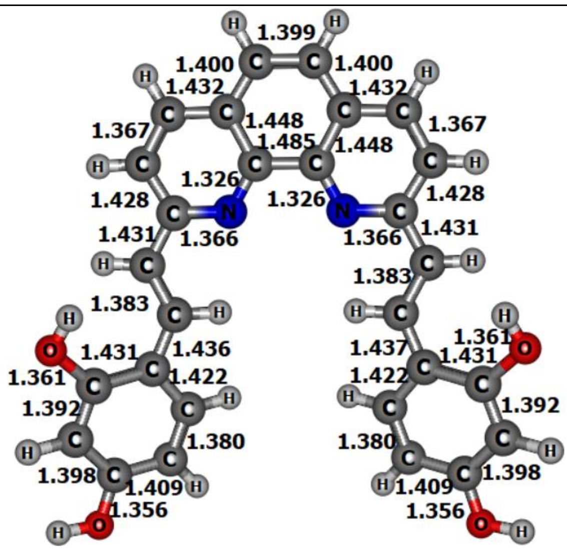

trans
Isomer 1 (S_1 state)

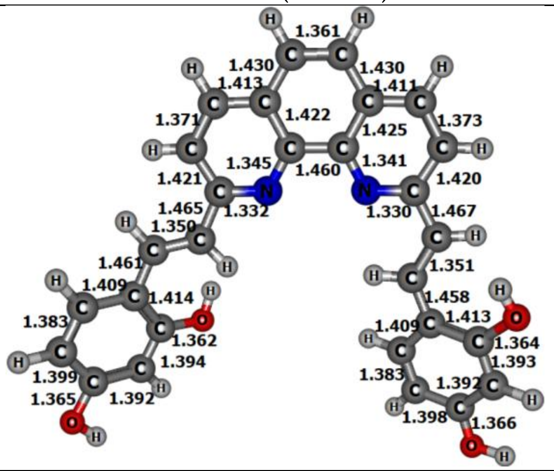

trans
Isomer 2 (S_0 state)

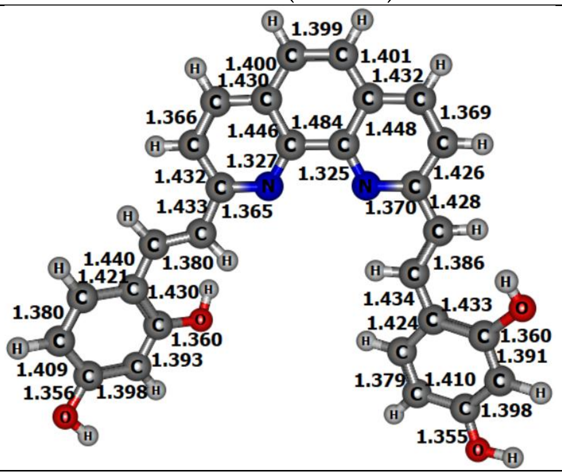

trans
Isomer 2 (S_1 state)

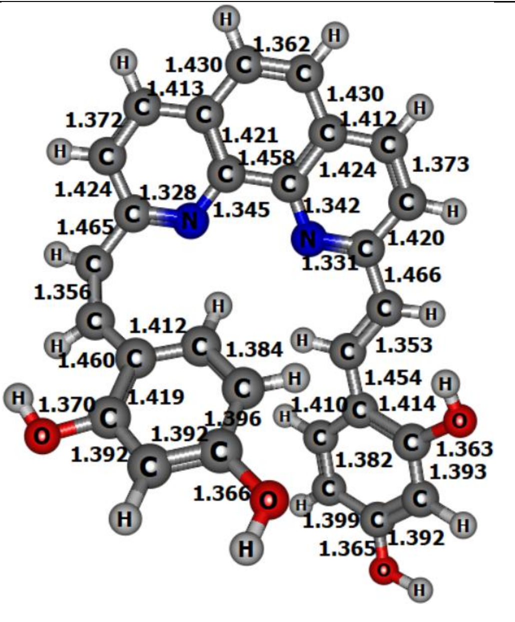

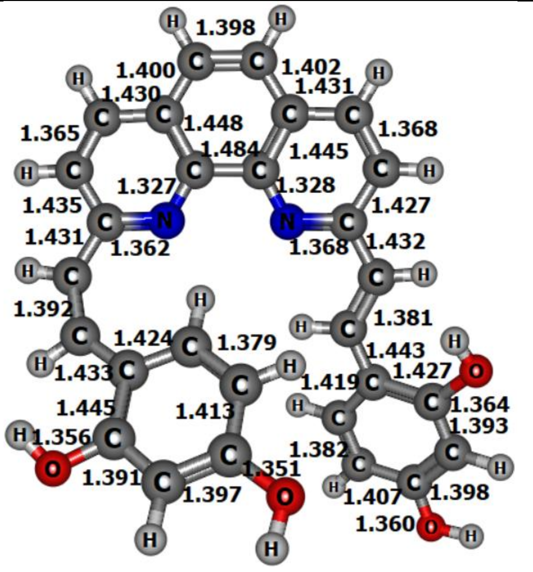

<table>
<tr><td>cis-trans
Isomer 3 (S_0 state)</td><td>cis-trans
Isomer 3 (S_1 state)</td></tr>
<tr><td>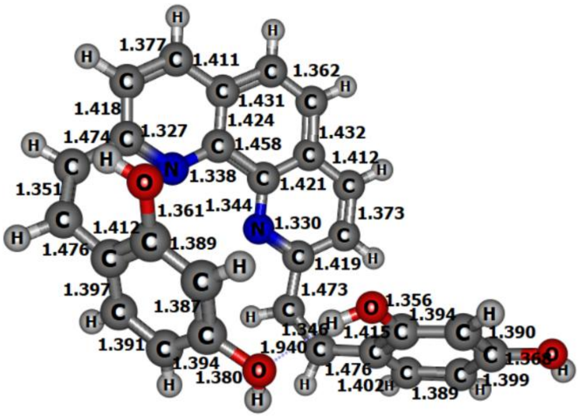</td><td>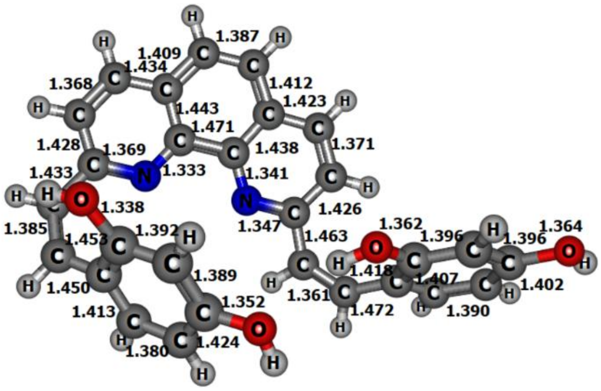</td></tr>
<tr><td>cis
Isomer 4 (S_0 state)</td><td>cis
Isomer 4 (S_1 state)</td></tr>
<tr><td>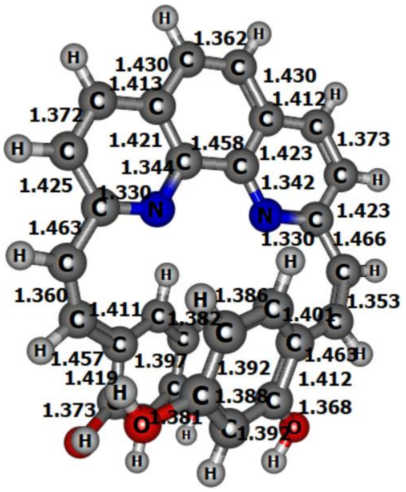</td><td>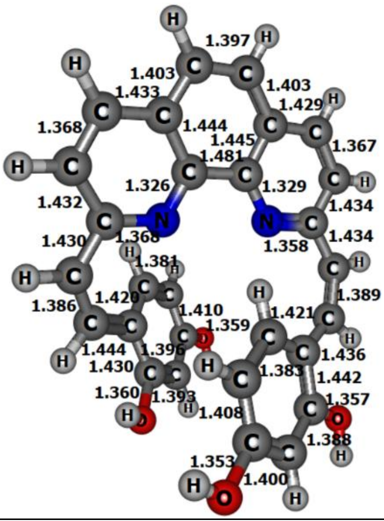</td></tr>
<tr><td>cis
Isomer 5 (S_0 state)</td><td>cis
Isomer 5 (S_1 state)</td></tr>
<tr><td>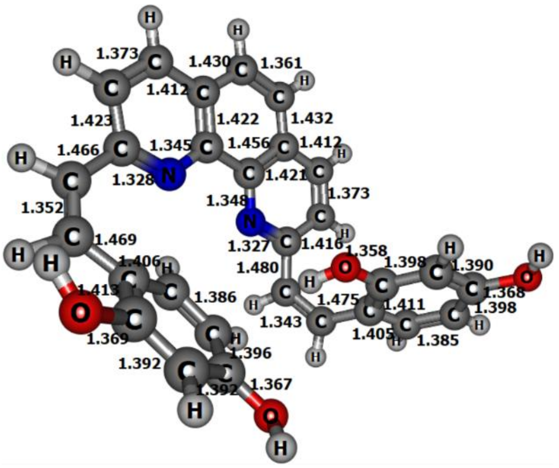</td><td>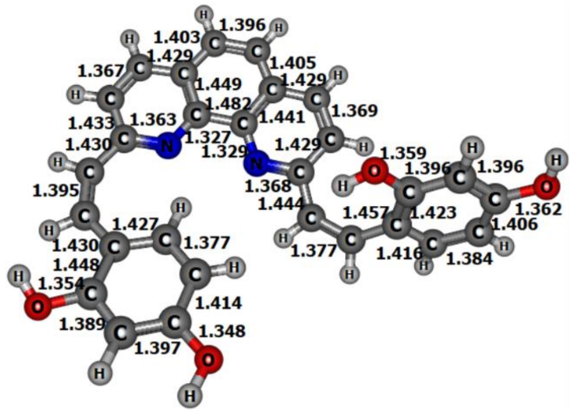</td></tr>
<tr><td>cis
Isomer 6 (S_0 state)</td><td>cis
Isomer 6 (S_1 state)</td></tr>
</table>

Figure S2. The optimized molecular structure and bond lengths (Å) of spatial *trans*, *cis*, and *cis-trans* rotamers of **3a** in the ground singlet S_0 state and the first excited S_1 state.

Testing the influence of functionals on the optimized geometry for the *trans* isomer of 3a

We chose the B3LYP-GD3/6-311++G(d,p) approach with inclusion of Grimme's D3 dispersion correction for geometry optimization because this functional and basis set combination has proven to be robust in accounting for common structural features and intramolecular interactions in organic systems. Although B3LYP can be considered somewhat outdated, it still offers a good balance between accuracy and computational cost, especially for geometry optimization of medium-sized systems. We have also checked the range-separated hybrid cam-B3LYP-GD3 functional with Grimme's D3 dispersion correction, long-range corrected hybrid density functional wB97XD, which includes empirical dispersion corrections, as well as the highly parametrized hybrid meta-GGA functional M06-2X, which incorporates 54% Hartree–Fock (HF) exchange with the 6-311++G(d,p) basis set (Fig. S3). The minimal structural changes (0.010–0.015 Å, Fig. S3) found during testing of these more modern functionals confirm that the B3LYP/6-311++G(d,p) level of theory is adequate to describe the key structural features of our systems. Furthermore, the small structural differences in bond length obtained using these alternative functionals (cam-B3LYP-GD3, wB97XD, and M06-2X) provide confidence that the structural conclusions made from the B3LYP-GD3 calculations remain valid and reliable.

<table>
<tr>
<td>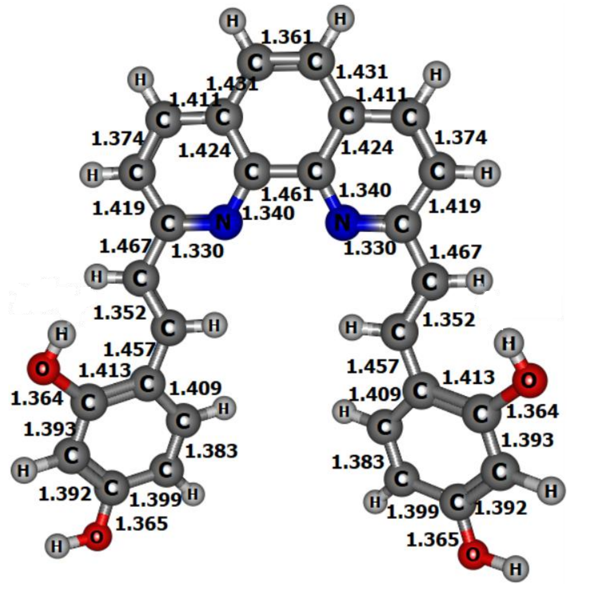
</td>
<td>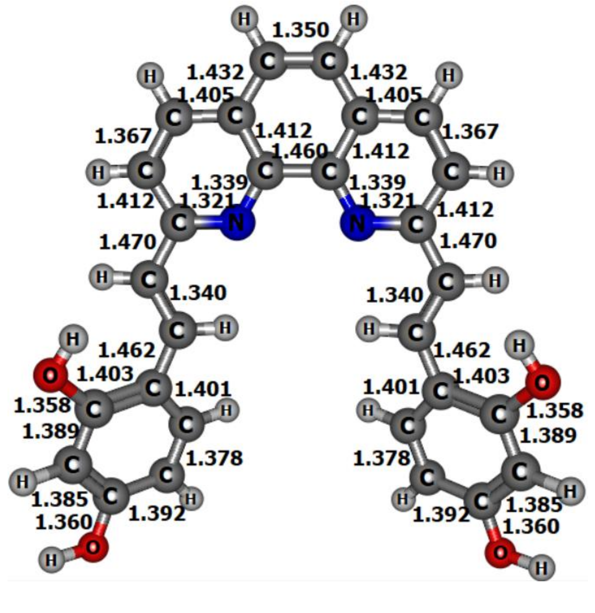
</td>
</tr>
<tr>
<td>trans
3a</td>
<td>trans
3a</td>
</tr>
<tr>
<td>B3LYP-GD3/6-311++g(d,p)</td>
<td>cam-B3LYP-GD3/6-311++g(d,p)</td>
</tr>
</table>

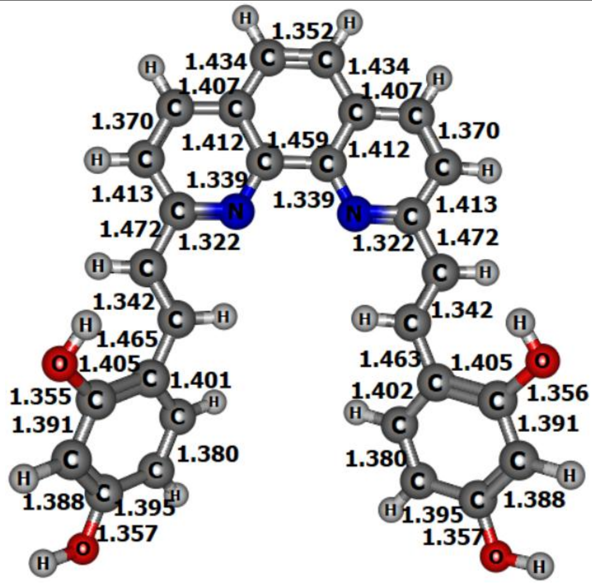	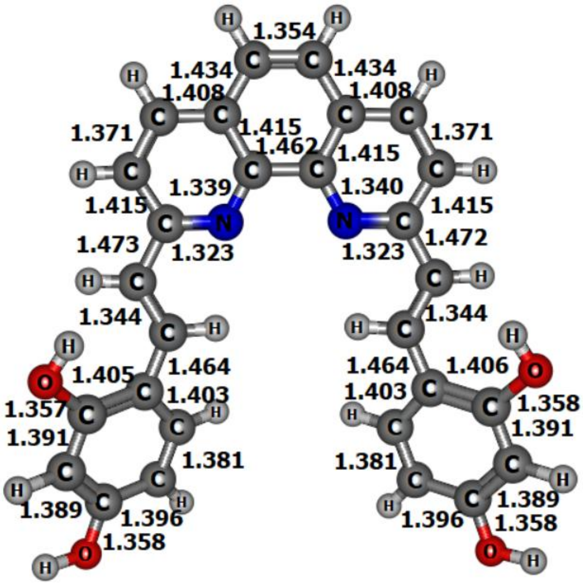
trans<br>**3a**	*trans*<br>**3a**
**wB97XD/6-311++g(d,p)**	**M06-2X/6-311++g(d,p)**

Figure S3. The optimized geometry of the *trans* rotamer of **3a** with different functionals B3LYP-GD3, cam-B3LYP-GD3, wB97XD, and M06-2X.

Testing the 6-31G(d,p) and 6-31+G(d,p) basis sets for absorption spectrum calculations

We have calculated absorption spectra for the *trans* rotamer of **3a** with B3LYP functional using both the 6-31G(d,p) and 6-31+G(d,p) basis sets (Fig. S4). Both spectra have the same overall shape. For comparison, the first band corresponds to the $S_0 \rightarrow S_1$ transition, calculated at 428 nm with the B3LYP/6-31G(d,p) approach and at 431 nm with the B3LYP/6-31+G(d,p) approach (Fig. S4). The second absorption band was calculated in the 365–400 nm range, with maxima at 377 nm (B3LYP/6-31G(d,p)) and 384 nm (B3LYP/6-31+G(d,p)), as shown in Figure S4. The third band maximum was calculated at 334 nm using the B3LYP/6-31G(d,p) and at 336 nm with the B3LYP/6-31+G(d,p) approach (Fig. S4). Such small shifts (~2–7 nm, red shift) are within the typical range of basis set effects when adding diffuse functions. This demonstrates that the diffuse functions only introduce minor corrections to the electronic excitation energies. Therefore, our choice of a smaller 6-31G(d,p) basis set for TDDFT was a pragmatic decision, given the computational demands of excited state calculations and the relatively small impact of this on the predicted transition energies compared to geometry inaccuracies.

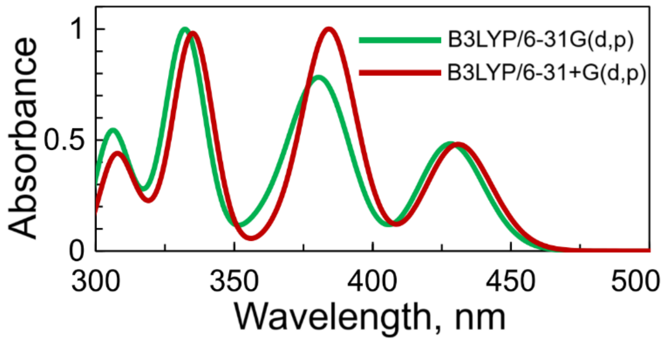

Figure S4. TDDFT calculated absorption spectra of the *trans* rotamer of **3a** calculated with B3LYP functional using both 6-31G(d,p) and 6-31+G(d,p) basis sets.

Testing the solvent effects on the optimized geometry for the *trans* isomer of 3a

The solvent effect was taken into account only in the TDDFT calculations because the minimal structural changes upon solvation do not significantly affect the optimized geometry (Fig. S5). Therefore, geometry optimization was performed in the gas phase, and the solvent effect was considered only in the excited state calculations to account for the environmental effects on the UV-visible absorption and emission spectra. We have calculated the optimized geometry of the *trans* rotamer of **3a** both in the gas phase and with the dimethylsulfoxide solvent effects included (Fig. S5). The comparison shows only negligible changes in bond lengths (0.001 Å, Fig. S5), which supports the decision to perform geometry optimizations in the gas phase.

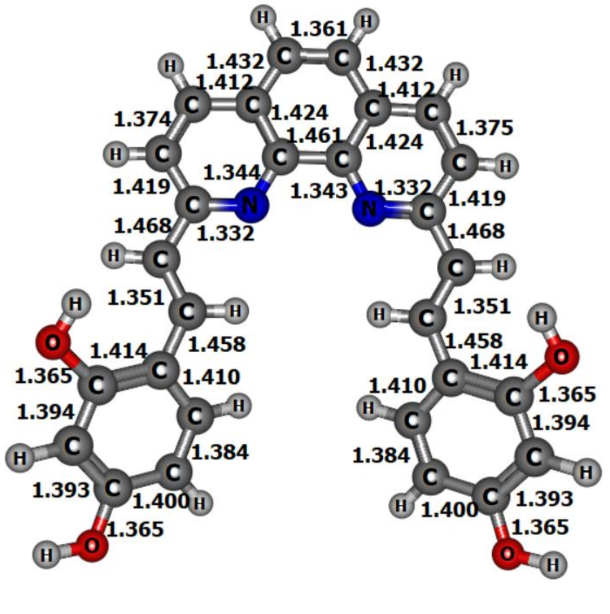

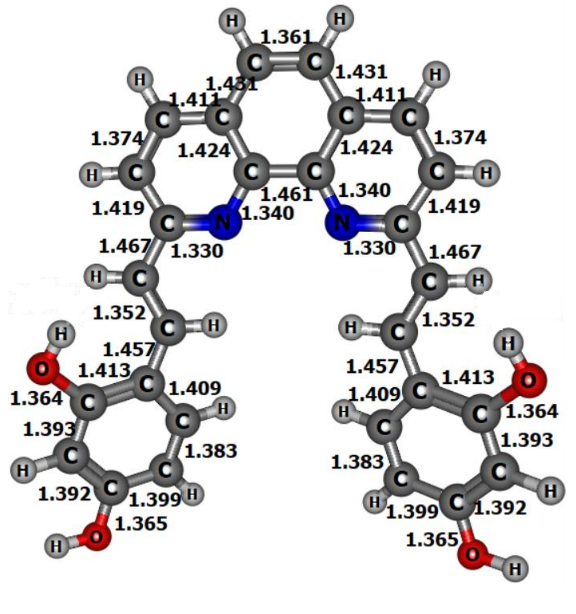

trans **3a**	*trans* **3a**
B3LYP-GD3/6-311++g(d,p) **with accounting for the DMSO solvent effects**	B3LYP-GD3/6-311++g(d,p) **in the gas phase**

Figure S5. The B3LYP-GD3/6-311++g(d,p) optimized geometry of the *trans* rotamer of **3a** in the gas phase (right) and with the dimethylsulfoxide solvent effects included (left).

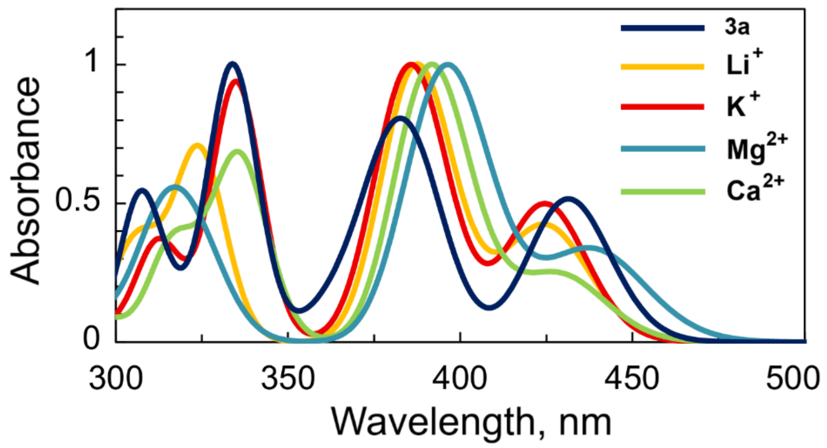

Figure S6. TDDFT calculated absorption spectra of the complexes of the *trans* rotamer of **3a** with Li^{+}, K^{+}, Mg^{2+}, and Ca^{2+} ions calculated with B3LYP/6-31G(d,p) method.

Table S1. Molecular orbitals and corresponding energy levels for **3a** (**rotamers 1–6**), *trans* and *cis* rotamers of **3c**, and **3d** are calculated at the DFT/B3LYP/6-31G(d,p) level of theory

**Compound 3a**<br>**Trans rotamer 1**		
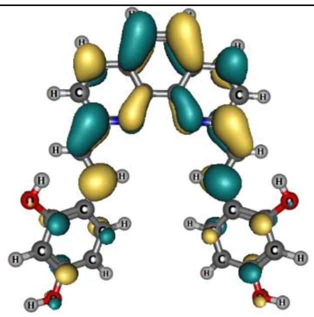	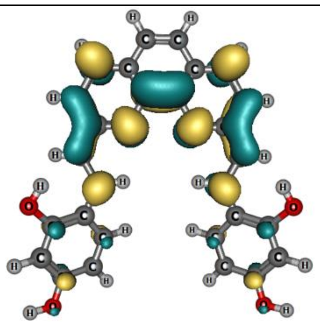	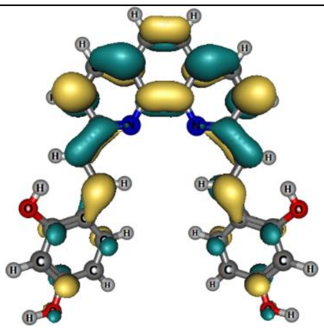
**LUMO (-1.88 eV)**	**LUMO+1 (-1.46 eV)**	**LUMO+2 (-1.08 eV)**
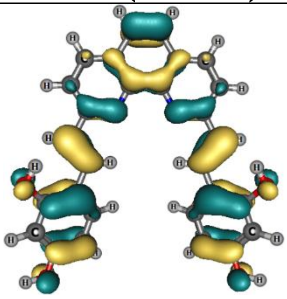	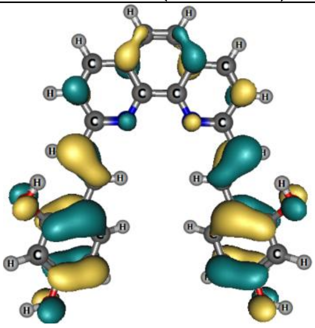	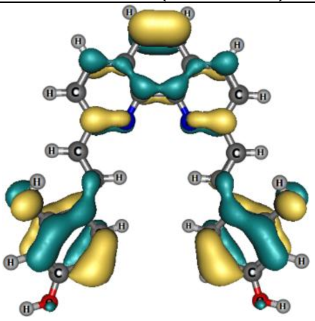
**HOMO (-5.23 eV)**	**HOMO-1 (-5.47 eV)**	**HOMO-2 (-6.24 eV)**
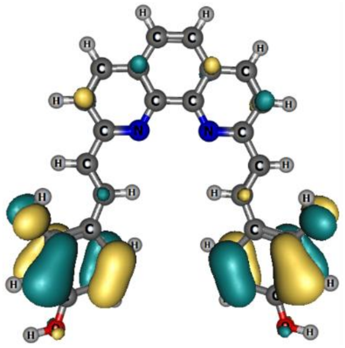	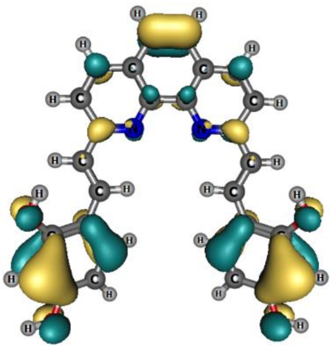	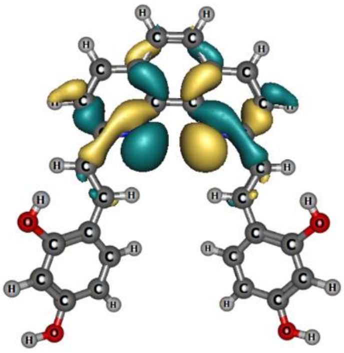
**HOMO-3 (-6.35 eV)**	**HOMO-4 (-6.49 eV)**	**HOMO-5 (-6.56 eV)**

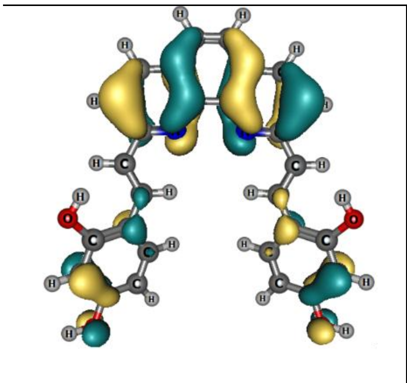	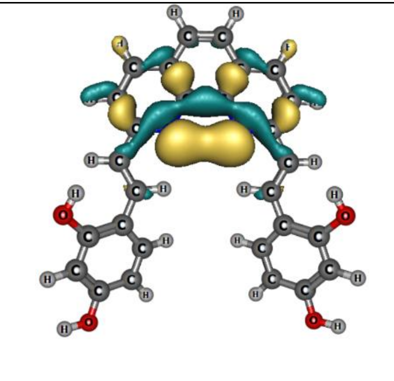	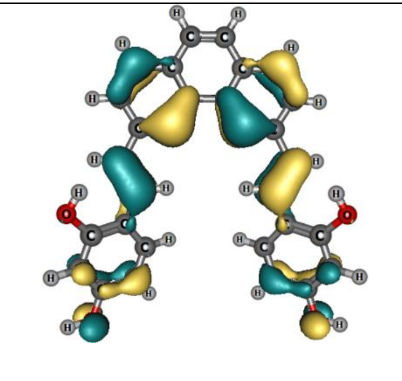
**HOMO-6 (-6.68 eV)**	**HOMO-7 (-6.96 eV)**	**HOMO-8 (-7.40 eV)**
	**Compound 3a**<br>**Trans rotamer 2**	
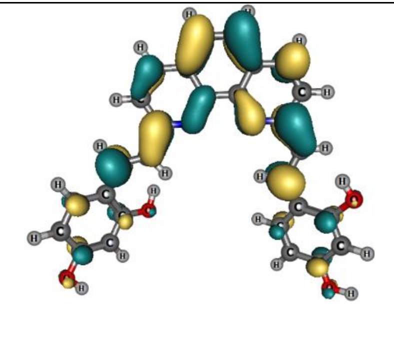	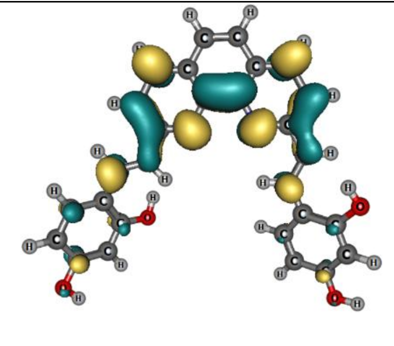	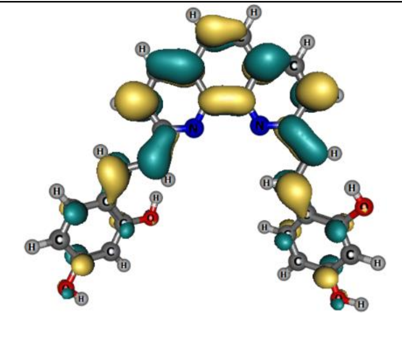
**LUMO (-1.89 eV)**	**LUMO+1 (-1.36 eV)**	**LUMO+2 (-1.18 eV)**
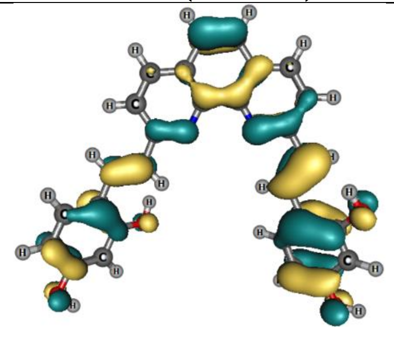	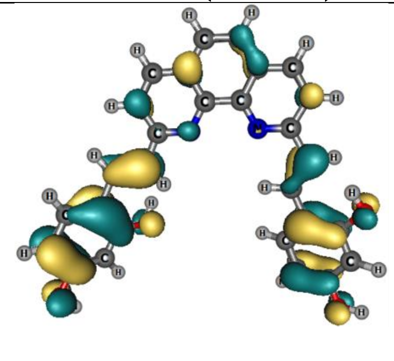	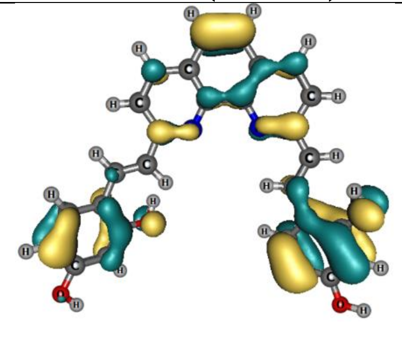
**HOMO (-5.25 eV)**	**HOMO-1 (-5.49 eV)**	**HOMO-2 (-6.23 eV)**
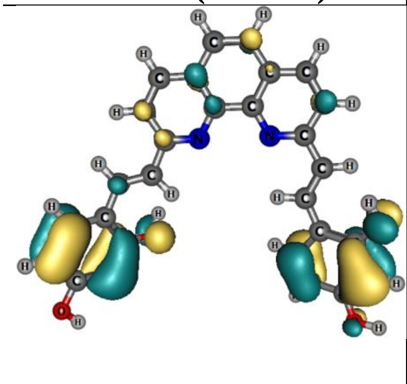	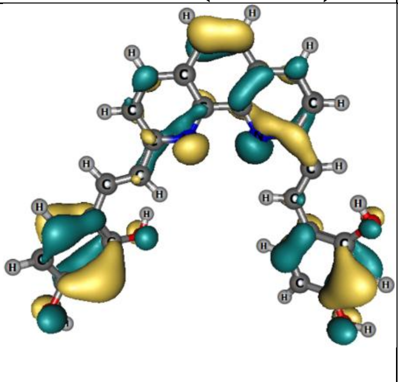	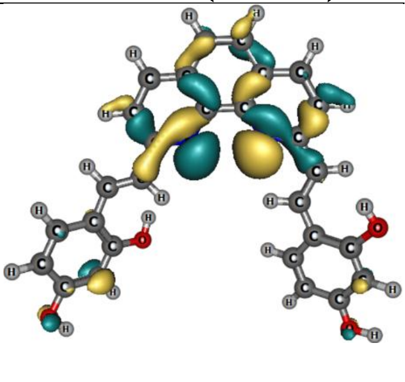
**HOMO-3 (-6.35 eV)**	**HOMO-4 (-6.48 eV)**	**HOMO-5 (-6.58 eV)**

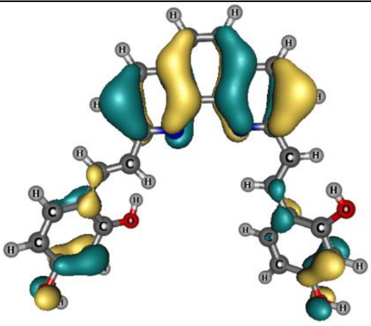	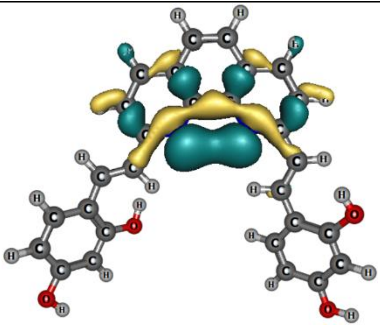	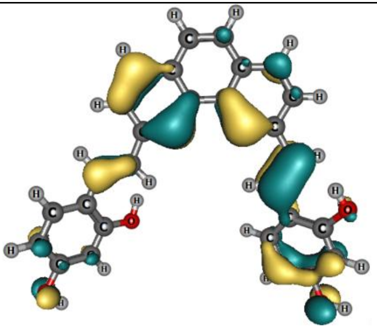
**HOMO-6 (-6.66 eV)**	**HOMO-7 (-6.96 eV)**	**HOMO-8 (-7.43 eV)**
	**Compound 3a**<br>**Cis-Trans rotamer 3**	
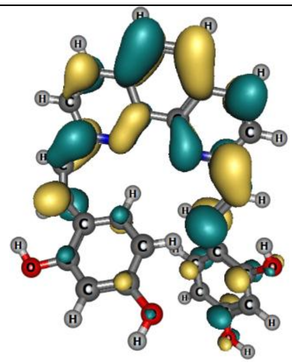	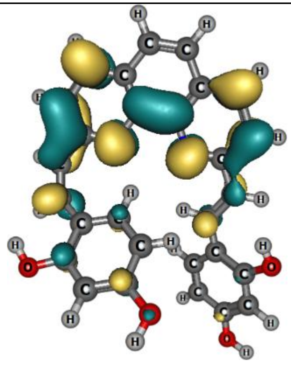	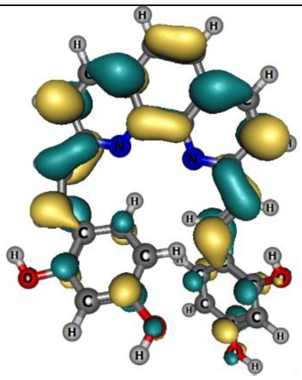
**LUMO (-1.85 eV)**	**LUMO+1 (-1.41 eV)**	**LUMO+2 (-1.08 eV)**
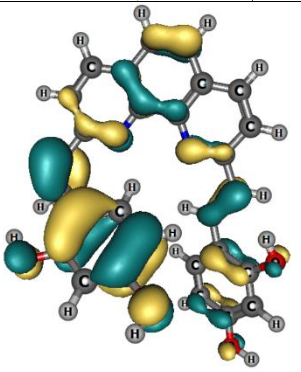	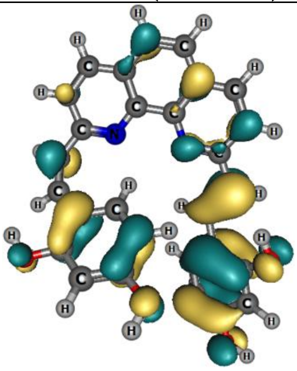	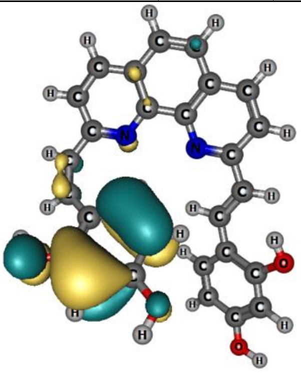
**HOMO (-5.17 eV)**	**HOMO-1 (-5.35 eV)**	**HOMO-2 (-6.03 eV)**
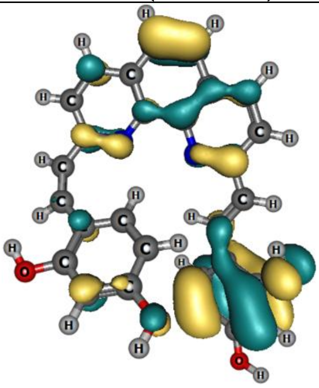	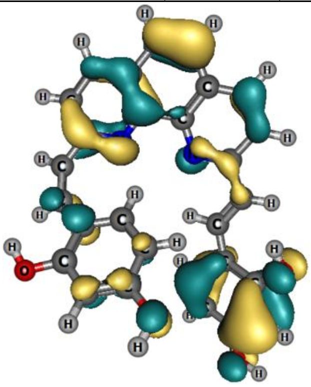	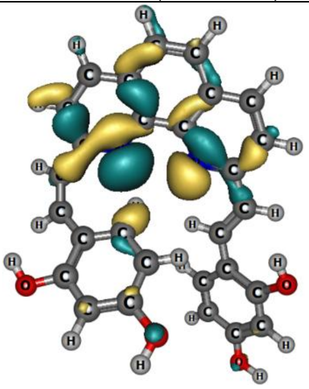
**HOMO-3 (-6.22 eV)**	**HOMO-4 (-6.39 eV)**	**HOMO-5 (-6.59 eV)**

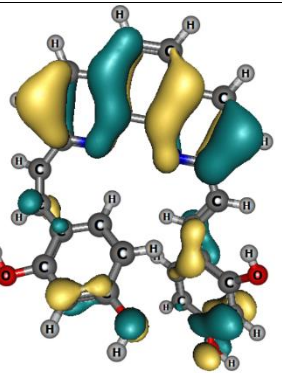	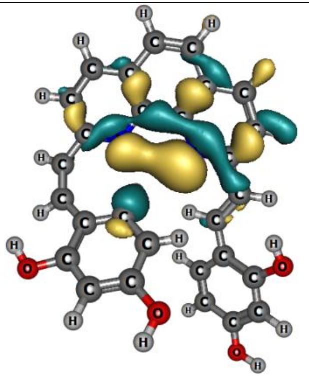	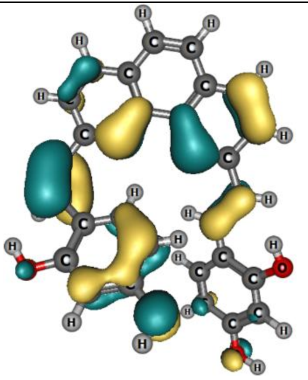
**HOMO-6 (-6.63 eV)**	**HOMO-7 (-6.90 eV)**	**HOMO-8 (-7.34 eV)**

Compound 3a
Cis rotamer 4

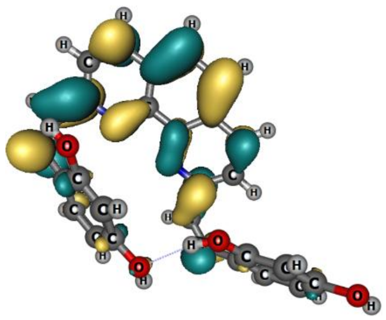	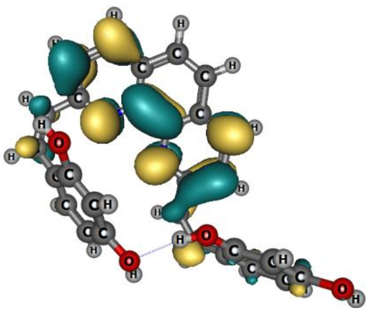	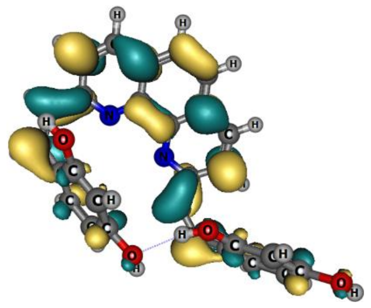
**LUMO (-1.77 eV)**	**LUMO+1 (-1.24 eV)**	**LUMO+2 (-1.05 eV)**
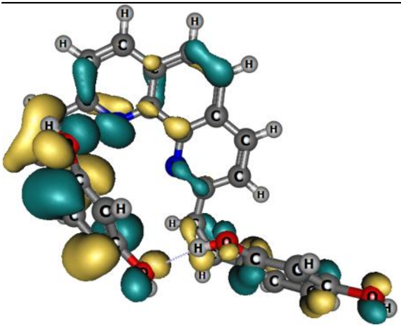	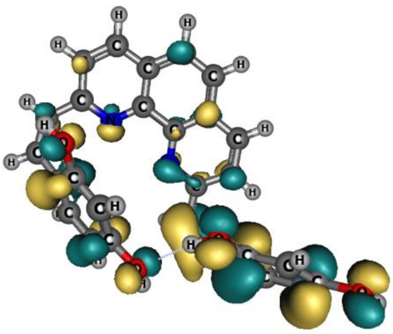	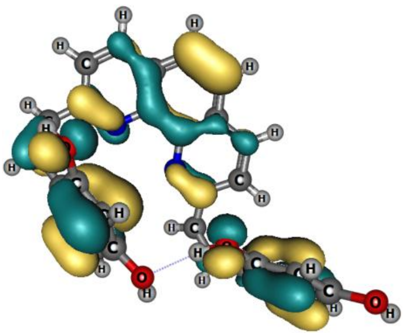
**HOMO (-5.33 eV)**	**HOMO-1 (-5.45 eV)**	**HOMO-2 (-5.96 eV)**
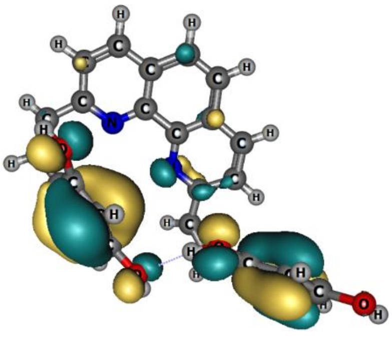	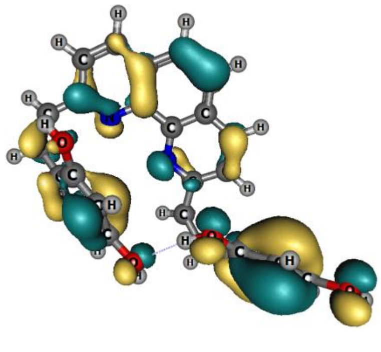	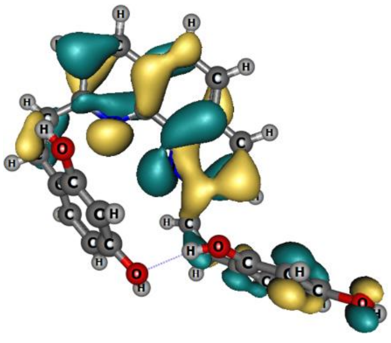
**HOMO-3 (-6.06 eV)**	**HOMO-4 (-6.19 eV)**	**HOMO-5 (-6.38 eV)**

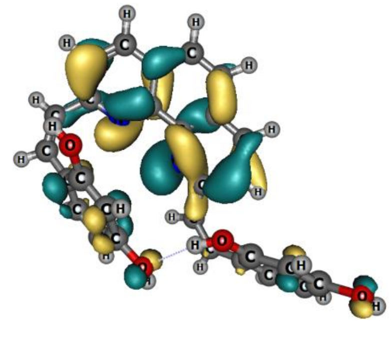	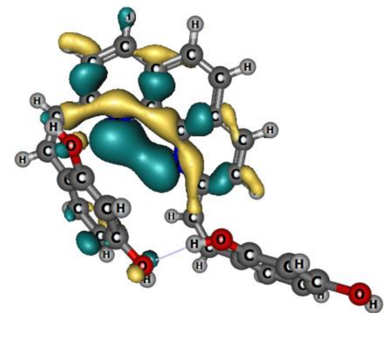	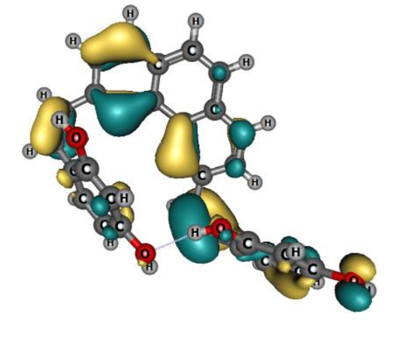
**HOMO-6 (-6.58 eV)**	**HOMO-7 (-6.84 eV)**	**HOMO-8 (-7.33 eV)**

Compound 3a
Cis rotamer 5

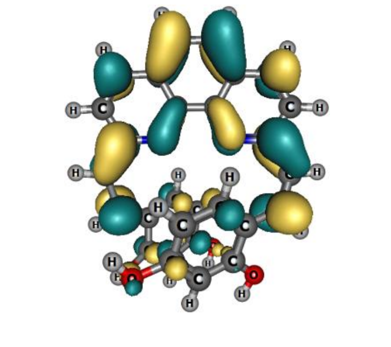	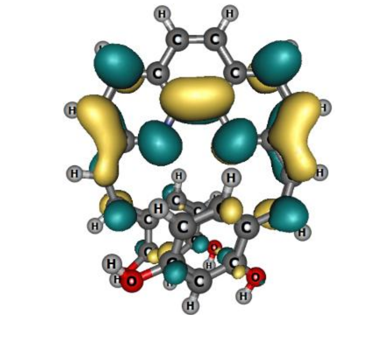	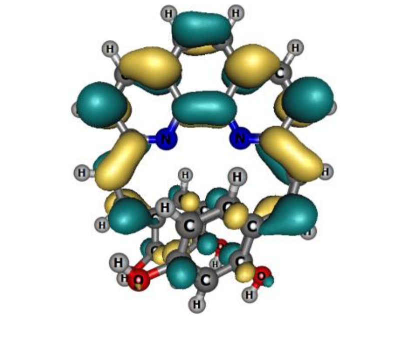
**LUMO (-1.78 eV)**	**LUMO+1 (-1.35 eV)**	**LUMO+2 (-0.98 eV)**
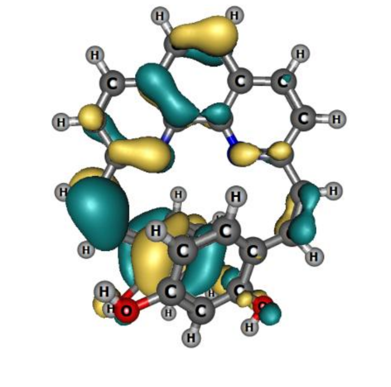	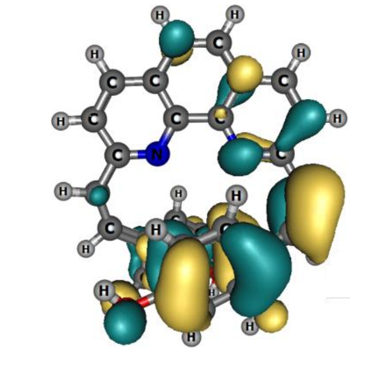	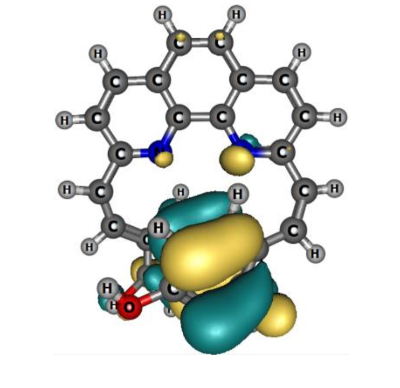
**HOMO (-5.10 eV)**	**HOMO-1 (-5.18 eV)**	**HOMO-2 (-5.96 eV)**
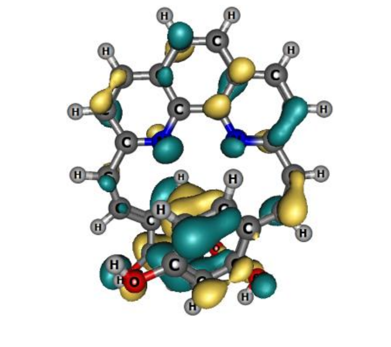	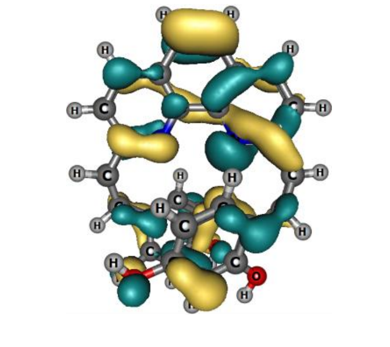	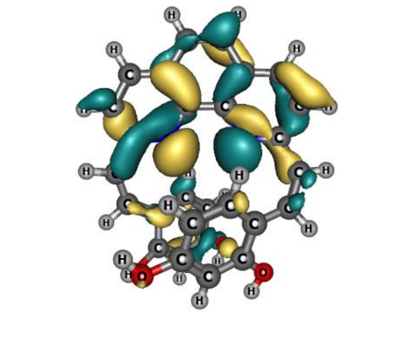
**HOMO-3 (-6.13 eV)**	**HOMO-4 (-6.27 eV)**	**HOMO-5 (-6.42 eV)**

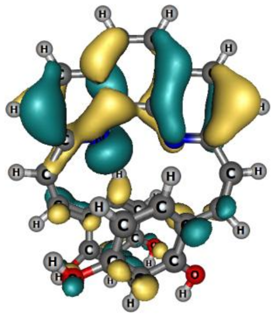	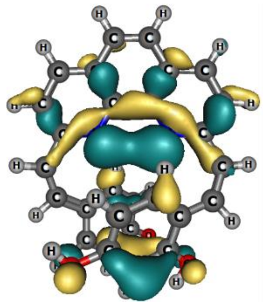	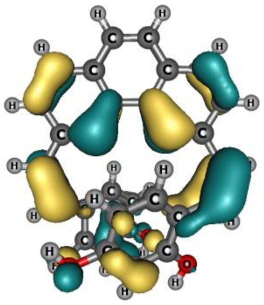
**HOMO-6 (-6.55 eV)**	**HOMO-7 (-6.85 eV)**	**HOMO-8 (-7.29 eV)**

Compound 3a
Cis rotamer 6

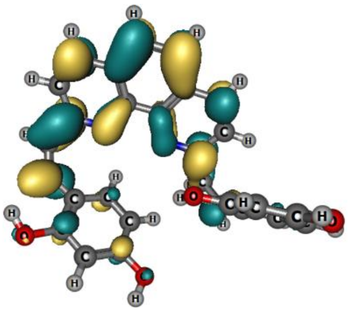	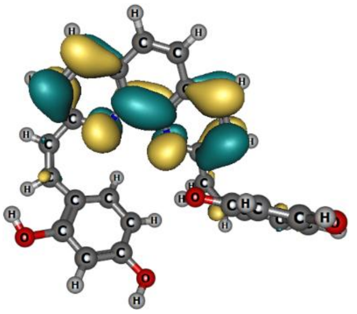	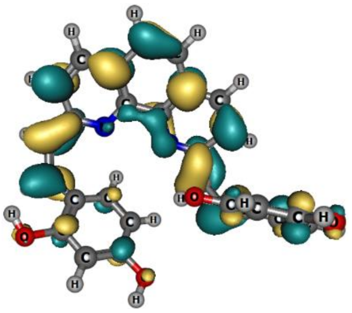
**LUMO (-1.78 eV)**	**LUMO+1 (-1.38 eV)**	**LUMO+2 (-0.94 eV)**
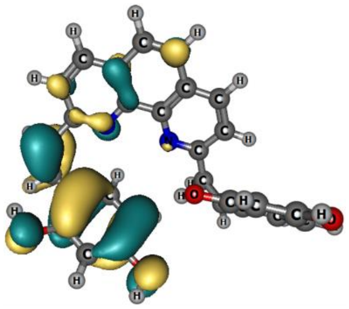	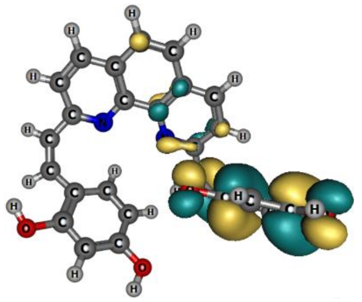	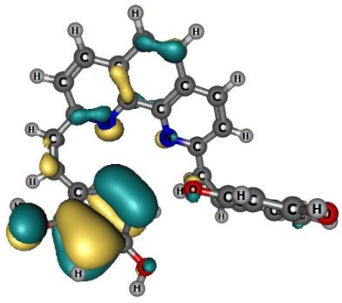
**HOMO (-5.33 eV)**	**HOMO-1 (-5.46 eV)**	**HOMO-2 (-6.04 eV)**
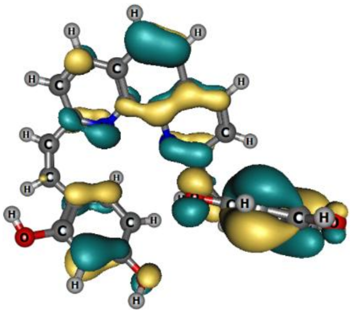	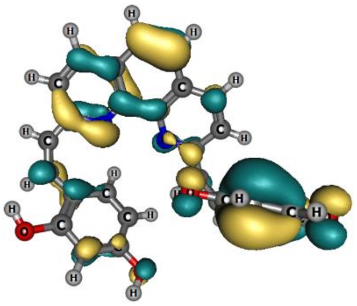	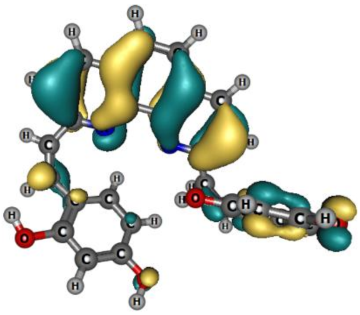
**HOMO-3 (-6.10 eV)**	**HOMO-4 (-6.27 eV)**	**HOMO-5 (-6.58 eV)**

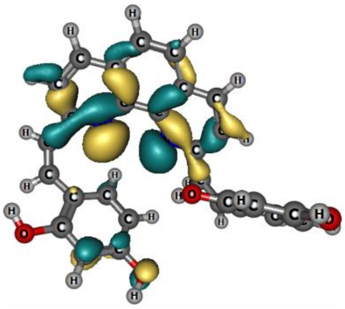	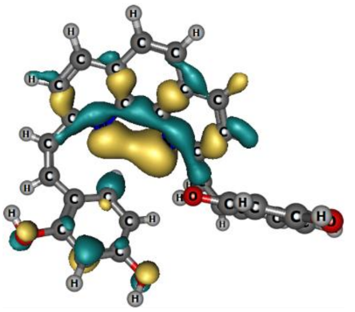	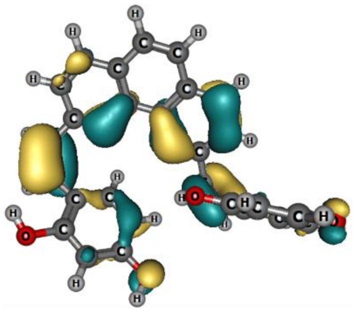
**HOMO-6 (-6.64 eV)**	**HOMO-7 (-6.97 eV)**	**HOMO-8 (-7.35 eV)**

Compound 3c
Trans

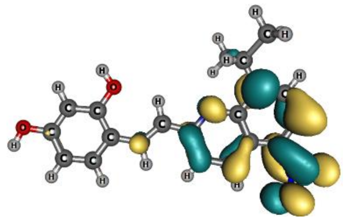	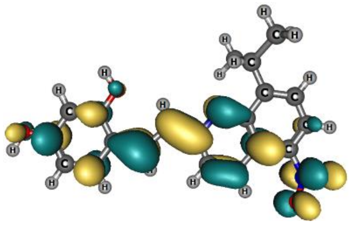	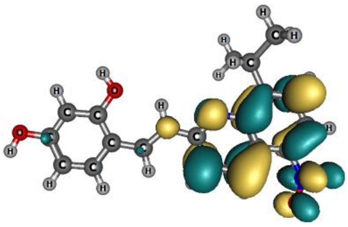
**LUMO (-2.27 eV)**	**LUMO+1 (-1.61 eV)**	**LUMO+2 (-0.92 eV)**
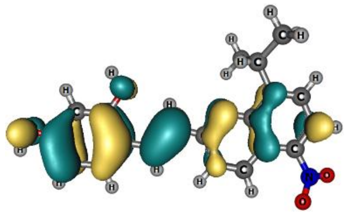	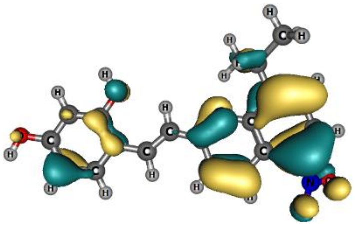	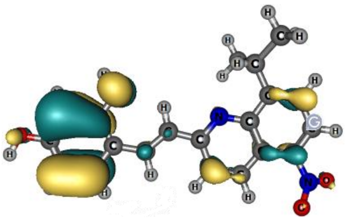
**HOMO (-5.53 eV)**	**HOMO-1 (-6.54 eV)**	**HOMO-2 (-6.69 eV)**
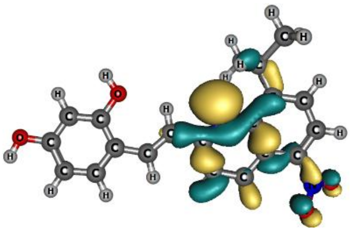	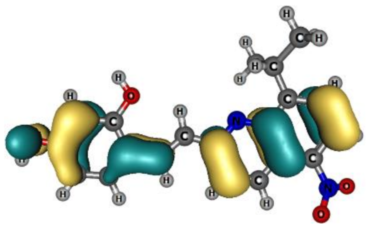	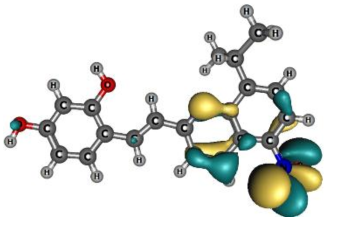
**HOMO-3 (-6.93 eV)**	**HOMO-4 (-7.06 eV)**	**HOMO-5 (-7.66 eV)**
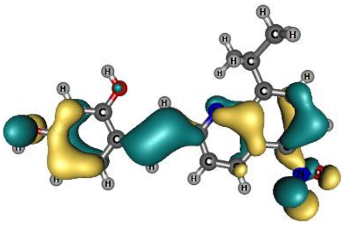	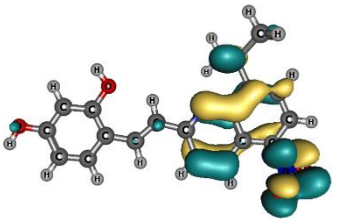	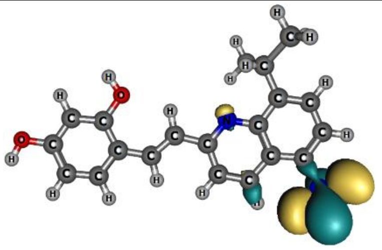
**HOMO-6 (-8.05 eV)**	**HOMO-7 (-8.17 eV)**	**HOMO-8 (-8.24 eV)**
	**Compound 3c**<br>**Cis**	

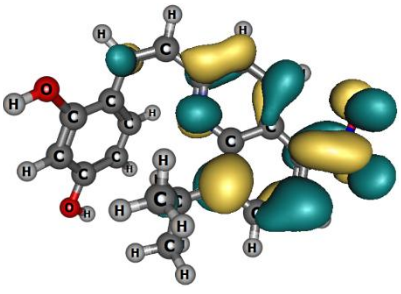	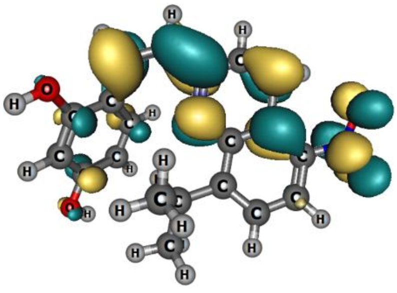	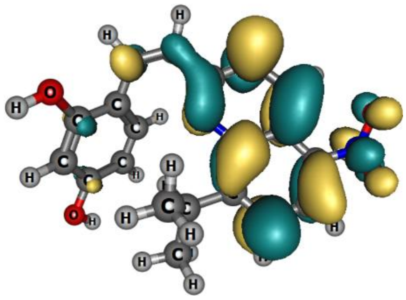
**LUMO (-2.37 eV)**	**LUMO+1 (-1.61 eV)**	**LUMO+2 (-0.91 eV)**
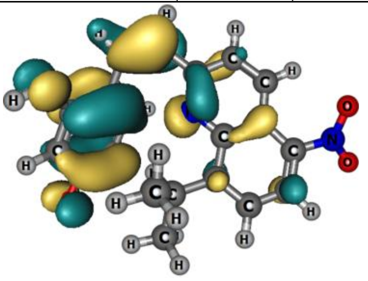	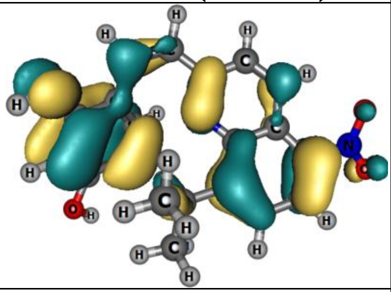	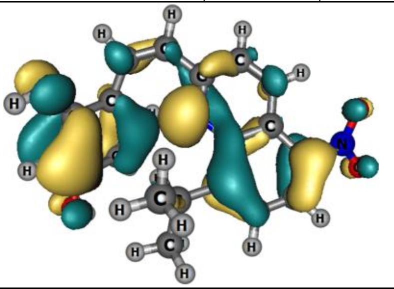
**HOMO (-5.64 eV)**	**HOMO-1 (-6.39 eV)**	**HOMO-2 (-6.57 eV)**
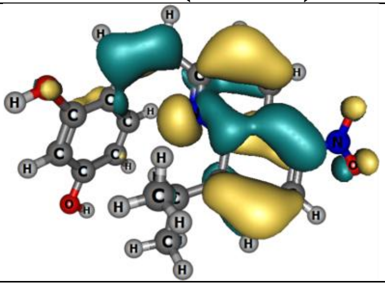	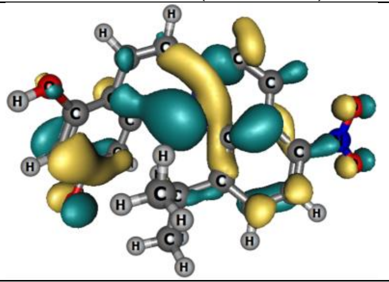	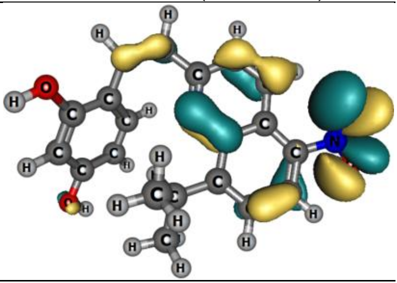
**HOMO-3 (-6.84 eV)**	**HOMO-4 (-7.10 eV)**	**HOMO-5 (-7.71 eV)**
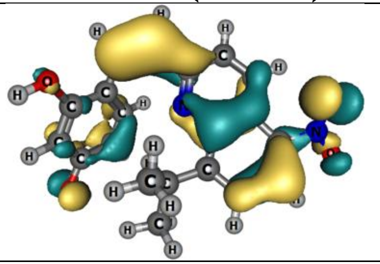	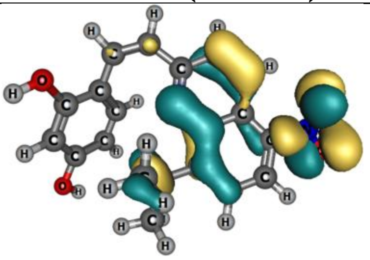	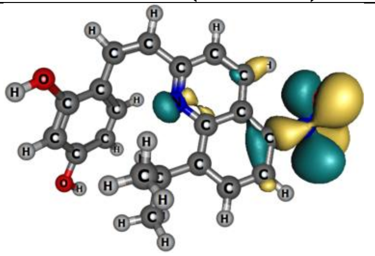
**HOMO-6 (-7.99 eV)**	**HOMO-7 (-8.26 eV)**	**HOMO-8 (-8.30 eV)**
	**Compound 3d**<br>**Trans**	
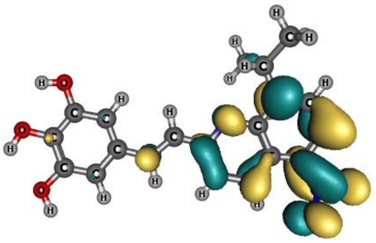	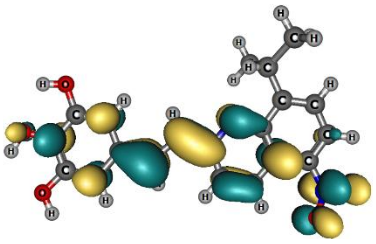	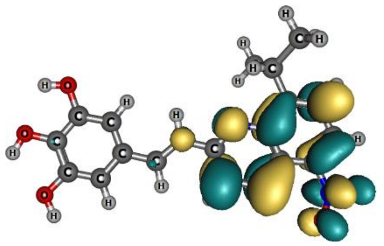
**LUMO (-2.37 eV)**	**LUMO+1 (-1.77 eV)**	**LUMO+2 (-1.03 eV)**

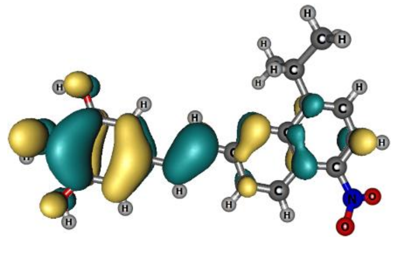	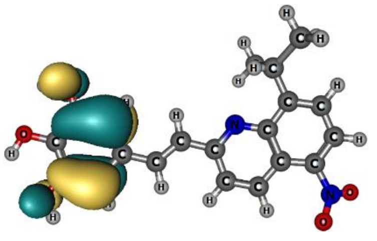	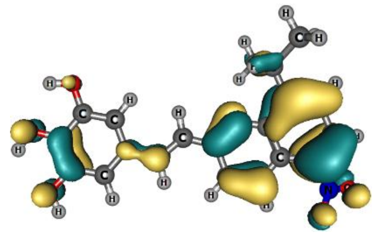
**HOMO (-5.55 eV)**	**HOMO-1 (-6.20 eV)**	**HOMO-2 (-6.59 eV)**
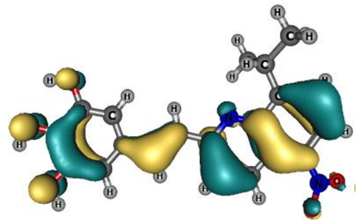	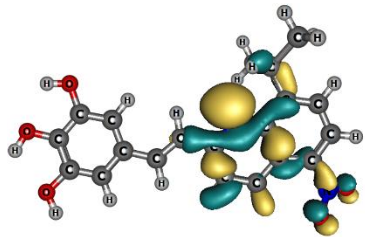	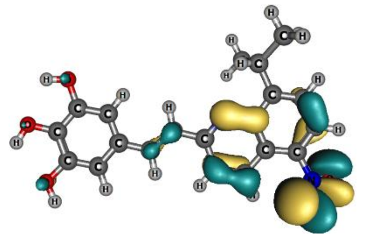
**HOMO-3 (-6.92 eV)**	**HOMO-4 (-7.08 eV)**	**HOMO-5 (-7.74 eV)**
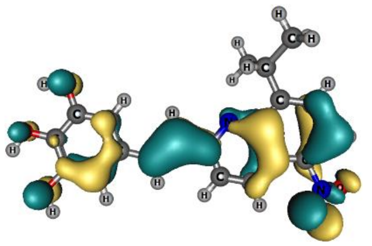	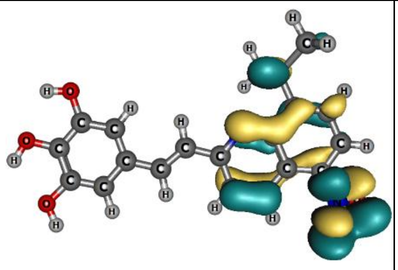	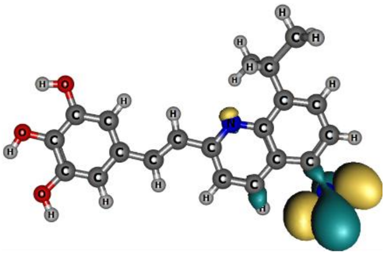
**HOMO-6 (-7.96 eV)**	**HOMO-7 (-8.25 eV)**	**HOMO-8 (-8.33 eV)**
	**Compound 3d**<br>**Cis**	
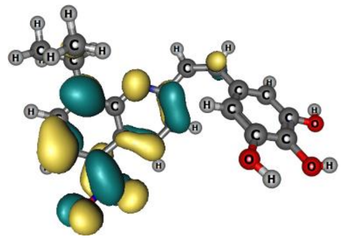	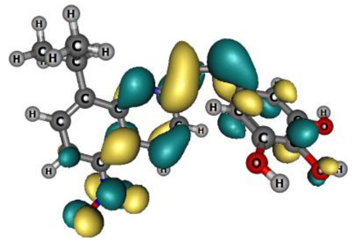	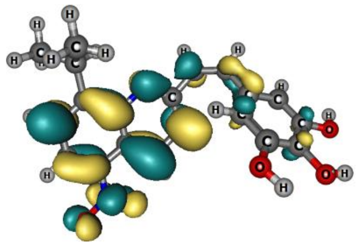
**LUMO (-2.31 eV)**	**LUMO+1 (-1.58 eV)**	**LUMO+2 (-0.96 eV)**
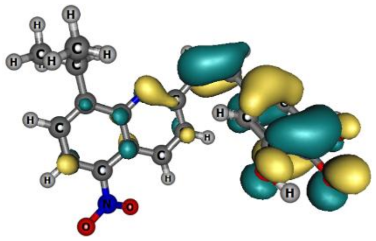	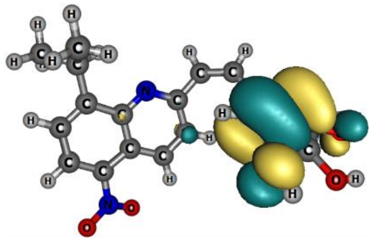	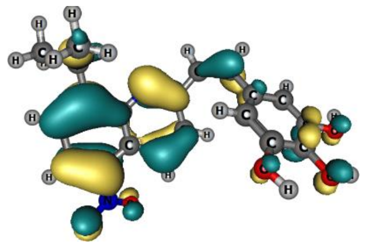
**HOMO (-5.65 eV)**	**HOMO-1 (-6.14 eV)**	**HOMO-2 (-6.50 eV)**

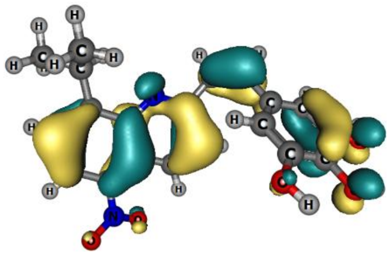	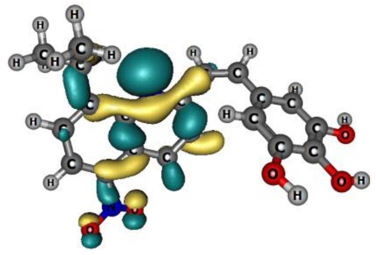	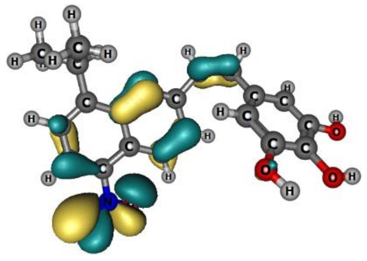
**HOMO-3 (-6.88 eV)**	**HOMO-4 (-7.04 eV)**	**HOMO-5 (-7.70 eV)**
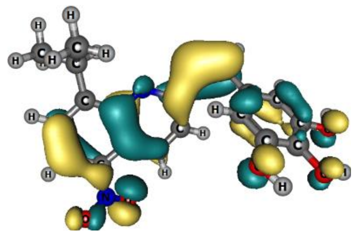	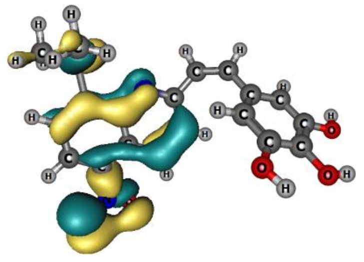	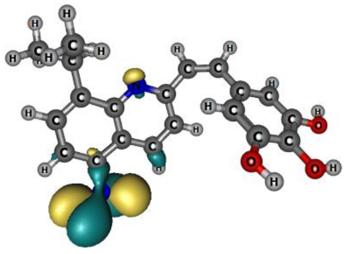
**HOMO-6 (-7.84 eV)**	**HOMO-7 (-8.21 eV)**	**HOMO-8 (-8.28 eV)**

Table S2. Wavelengths (λ), oscillator strengths (*f*), and orbital assignment of the selected electronic transitions in the absorption spectra of the **3a** rotamers **1**–**6** calculated at the TDDFT/B3LYP/6-31G(d,p) level of theory, taking into account the dimethylsulfoxide solvent effects.

Compounds	Transition	λ, nm	*f*	Assignment
**3a**<br>***Trans***<br>**rotamer 1**	$S_0 \rightarrow S_1$	428	0.3689	HOMO→LUMO (+99%)
	$S_0 \rightarrow S_2$	385	0.4182	HOMO-1→LUMO (+92%)
	$S_0 \rightarrow S_3$	374	0.2765	HOMO→LUMO+1 (+91%)
	$S_0 \rightarrow S_4$	359	0.0807	HOMO-1→LUMO+1 (+97%)
	$S_0 \rightarrow S_5$	332	0.7526	HOMO→LUMO+2 (+77%)<br>HOMO-3→LUMO (8%)<br>HOMO-5→LUMO (6%)
	$S_0 \rightarrow S_6$	320	0.0422	HOMO-6→LUMO (+55%)<br>HOMO-5→LUMO (+35%)
	$S_0 \rightarrow S_7$	317	0.0434	HOMO-2→LUMO (+86%)<br>HOMO-1→LUMO+2 (+10%)
	$S_0 \rightarrow S_8$	309	0.0013	HOMO-1→LUMO+2 (+63%)<br>HOMO-4→LUMO (+17%)<br>HOMO-2→LUMO (6%)
	$S_0 \rightarrow S_9$	307	0.0025	HOMO-6→LUMO+1 (+41%)<br>HOMO-5→LUMO+1 (+27%)<br>HOMO-7→LUMO (+21%)<br>HOMO-1→LUMO+2 (+9%)
	$S_0 \rightarrow S_{10}$	306	0.3962	HOMO-3→LUMO (+78%)<br>HOMO→LUMO+2 (+10%)<br>HOMO-2→LUMO+1 (6%)

**3a** *Trans* **rotamer 2**	$S_0 \rightarrow S_1$	422	0.5993	HOMO→LUMO (+99%)
	$S_0 \rightarrow S_2$	382	0.4612	HOMO-1→LUMO (+95%)
	$S_0 \rightarrow S_3$	360	0.0673	HOMO→LUMO+1 (+87%)<br>HOMO→LUMO+2 (8%)
	$S_0 \rightarrow S_4$	346	0.1336	HOMO-1→LUMO+1 (+89%)<br>HOMO→LUMO+2 (5%)
	$S_0 \rightarrow S_5$	336	0.8592	HOMO→LUMO+2 (+71%)<br>HOMO→LUMO+1 (+10%)<br>HOMO-3→LUMO (6%)<br>HOMO-1→LUMO+1 (+5%)
	$S_0 \rightarrow S_6$	320	0.0341	HOMO-6→LUMO (+72%)<br>HOMO-2→LUMO (10%)
	$S_0 \rightarrow S_7$	316	0.0874	HOMO-2→LUMO (+51%)<br>HOMO-1→LUMO+2 (+33%)<br>HOMO-6→LUMO (+10%)
	$S_0 \rightarrow S_8$	313	0.0068	HOMO-1→LUMO+2 (+55%)<br>HOMO-2→LUMO (28%)<br>HOMO-4→LUMO (+9%)
	$S_0 \rightarrow S_9$	307	0.2890	HOMO-3→LUMO (+82%)<br>HOMO→LUMO+2 (+7%)
	$S_0 \rightarrow S_{10}$	299	0.0034	HOMO-6→LUMO+1 (+52%)<br>HOMO-7→LUMO (30%)
**3a** ***Cis-Trans*** **rotamer 3**	$S_0 \rightarrow S_1$	430	0.1561	HOMO→LUMO (+99%)
	$S_0 \rightarrow S_2$	392	0.4459	HOMO-1→LUMO (+93%)
	$S_0 \rightarrow S_3$	371	0.1900	HOMO→LUMO+1 (+86%)
	$S_0 \rightarrow S_4$	359	0.0287	HOMO-1→LUMO+1 (+92%)<br>HOMO→LUMO+1 (+5%)
	$S_0 \rightarrow S_5$	334	0.6086	HOMO→LUMO+2 (+73%)<br>HOMO-2→LUMO (8%)<br>HOMO-3→LUMO (6%)
	$S_0 \rightarrow S_6$	324	0.1163	HOMO-2→LUMO (+57%)<br>HOMO-6→LUMO (20%)<br>HOMO→LUMO+2 (+8%)
	$S_0 \rightarrow S_7$	322	0.0216	HOMO-6→LUMO (+27%)<br>HOMO-1→LUMO+2 (24%)<br>HOMO-2→LUMO (+20%)<br>HOMO-3→LUMO (18%)<br>HOMO-4→LUMO (7%)
	$S_0 \rightarrow S_8$	316	0.0399	HOMO-1→LUMO+2 (+56%)<br>HOMO-6→LUMO (+26%)<br>HOMO-2→LUMO (+10%)
	$S_0 \rightarrow S_9$	311	0.1054	HOMO-3→LUMO (+61%)<br>HOMO-4→LUMO (14%)<br>HOMO-6→LUMO (+6%)
	$S_0 \rightarrow S_{10}$	307	0.0083	HOMO-6→LUMO+1 (+49%)

				HOMO-7→LUMO (32%)<br>HOMO-3→LUMO+1 (6%)
**3a**<br>***Cis***<br>**rotamer 4**	S_0→S_1	414	0.0838	HOMO→LUMO (+79%)<br>HOMO-1→LUMO (15%)
	S_0→S_2	387	0.2500	HOMO-1→LUMO (+79%)<br>HOMO→LUMO (+16%)
	S_0→S_3	359	0.0057	HOMO→LUMO+1 (+68%)<br>HOMO-1→LUMO+1 (25%)
	S_0→S_4	344	0.0153	HOMO-1→LUMO+1 (+61%)<br>HOMO→LUMO+1 (+21%)<br>HOMO-2→LUMO (8%)
	S_0→S_5	333	0.0878	HOMO→LUMO+2 (+68%)<br>HOMO-3→LUMO (+16%)<br>HOMO-5→LUMO (+5%)
	S_0→S_6	332	0.1602	HOMO-2→LUMO (+80%)<br>HOMO-3→LUMO (+6%)
	S_0→S_7	323	0.0389	HOMO-5→LUMO (+28%)<br>HOMO-4→LUMO (24%)<br>HOMO-3→LUMO (19%)<br>HOMO-6→LUMO (14%)
	S_0→S_8	319	0.0595	HOMO-1→LUMO+2 (+61%)<br>HOMO-3→LUMO (+19%)
	S_0→S_9	313	0.2049	HOMO-3→LUMO (+28%)<br>HOMO→LUMO+2 (21%)<br>HOMO-1→LUMO+2 (20%)<br>HOMO-6→LUMO (10%)<br>HOMO-5→LUMO (+9%)
	S_0→S_{10}	307	0.0149	HOMO-4→LUMO (+55%)<br>HOMO-6→LUMO (13%)<br>HOMO-5→LUMO (+10%)
	S_0→S_{11}	304	0.0136	HOMO-2→LUMO+1 (+39%)<br>HOMO-6→LUMO (+24%)<br>HOMO-3→LUMO (7%)<br>HOMO-4→LUMO (+6%)
**3a**<br>***Cis***<br>**rotamer 5**	S_0→S_1	444	0.0637	HOMO→LUMO (+81%)<br>HOMO-1→LUMO (17%)
	S_0→S_2	419	0.2335	HOMO-1→LUMO (+78%)<br>HOMO→LUMO (+17%)
	S_0→S_3	383	0.0147	HOMO-1→LUMO+1 (+90%)<br>HOMO→LUMO+1 (+8%)
	S_0→S_4	379	0.1381	HOMO→LUMO+1 (+85%)<br>HOMO-1→LUMO+1 (8%)
	S_0→S_5	345	0.0041	HOMO-2→LUMO (+75%)<br>HOMO-5→LUMO (6%)<br>HOMO-4→LUMO (5%)
	S_0→S_6	339	0.2398	HOMO→LUMO+2 (+59%)<br>HOMO-5→LUMO (+13%)<br>HOMO-3→LUMO (12%)

				HOMO-2→LUMO (+10%)
	S_0→S_7	333	0.1517	HOMO-1→LUMO+2 (+50%)<br>HOMO-6→LUMO (12%)<br>HOMO-5→LUMO (+12%)<br>HOMO-4→LUMO (+9%)<br>HOMO-3→LUMO (6%)<br>HOMO→LUMO+2 (5%)
	S_0→S_8	332	0.0503	HOMO-1→LUMO+2 (+37%)<br>HOMO-5→LUMO (27%)<br>HOMO→LUMO+2 (+15%)<br>HOMO-6→LUMO (+10%)
	S_0→S_9	322	0.0036	HOMO-3→LUMO (+32%)<br>HOMO-4→LUMO (+31%)<br>HOMO-5→LUMO+1 (8%)<br>HOMO-2→LUMO (+7%)<br>HOMO-7→LUMO (+6%)
	S_0→S_{10}	312	0.1694	HOMO-3→LUMO (+30%)<br>HOMO-5→LUMO+1 (+21%)<br>HOMO-6→LUMO+1 (9%)<br>HOMO→LUMO+2 (+9%)<br>HOMO-4→LUMO+1 (+6%)
	S_0→S_{11}	310	0.0110	HOMO-2→LUMO+1 (+81%)
	S_0→S_{12}	307	0.0762	HOMO-4→LUMO (+33%)<br>HOMO-5→LUMO+1 (+16%)<br>HOMO-5→LUMO (14%)<br>HOMO-3→LUMO (11%)<br>HOMO-3→LUMO+1 (8%)
**3a**<br>***Cis***<br>**rotamer 6**	S_0→S_1	395	0.2308	HOMO→LUMO (+83%)<br>HOMO-1→LUMO (15%)
	S_0→S_2	375	0.1776	HOMO-1→LUMO (+92%)
	S_0→S_3	357	0.0082	HOMO→LUMO+1 (+94%)
	S_0→S_4	341	0.0370	HOMO-1→LUMO+1 (+91%)
	S_0→S_5	327	0.0191	HOMO-3→LUMO (+63%)<br>HOMO-6→LUMO (+19%)<br>HOMO-4→LUMO (+5%)
	S_0→S_6	321	0.1160	HOMO-2→LUMO (+82%)<br>HOMO→LUMO+2 (+7%)
	S_0→S_7	316	0.0811	HOMO→LUMO+2 (+37%)<br>HOMO-5→LUMO (16%)<br>HOMO-2→LUMO+1 (12%)<br>HOMO-3→LUMO (+9%)<br>HOMO-6→LUMO (8%)<br>HOMO-4→LUMO (7%)
	S_0→S_8	307	0.1819	HOMO→LUMO+2 (+40%)<br>HOMO-4→LUMO (+25%)<br>HOMO-3→LUMO (10%)<br>HOMO-2→LUMO (6%)
	S_0→S_9	306	0.0148	HOMO-6→LUMO (+29%)<br>HOMO-2→LUMO+1 (28%)

				HOMO-6→LUMO+1 (7%)
	$S_0 \rightarrow S_{10}$	303	0.0838	HOMO-1→LUMO+2 (+72%)<br>HOMO-6→LUMO+1 (6%)
**3c**<br>***Trans***	$S_0 \rightarrow S_1$	474	0.1051	HOMO→LUMO (+99%)
	$S_0 \rightarrow S_2$	362	1.1841	HOMO→LUMO+1 (+93%)
	$S_0 \rightarrow S_3$	347	0.1009	HOMO-2→LUMO (+60%)<br>HOMO-1→LUMO (14%)<br>HOMO-3→LUMO (+7%)
	$S_0 \rightarrow S_4$	336	0.0452	HOMO-1→LUMO (+81%)<br>HOMO-2→LUMO (+13%)
	$S_0 \rightarrow S_5$	332	0.0279	HOMO-4→LUMO (+63%)<br>HOMO-3→LUMO (+15%)<br>HOMO-2→LUMO (7%)
	$S_0 \rightarrow S_6$	315	0.0671	HOMO→LUMO+2 (+72%)<br>HOMO-2→LUMO+1 (8%)<br>HOMO-5→LUMO (6%)
	$S_0 \rightarrow S_7$	310	0.0916	HOMO-3→LUMO (+43%)<br>HOMO-4→LUMO (20%)<br>HOMO→LUMO+2 (16%)<br>HOMO-5→LUMO (8%)
	$S_0 \rightarrow S_8$	307	0.0534	HOMO-5→LUMO (+27%)<br>HOMO-6→LUMO (+23%)<br>HOMO-3→LUMO (+16%)<br>HOMO-2→LUMO (15%)<br>HOMO-7→LUMO (6%)
	$S_0 \rightarrow S_9$	292	0.0908	HOMO-1→LUMO+1 (+82%)
	$S_0 \rightarrow S_{10}$	288	0.0190	HOMO-4→LUMO+1 (+42%)<br>HOMO-2→LUMO+1 (+19%)<br>HOMO-3→LUMO+1 (+15%)<br>HOMO-8→LUMO (7%)
	$S_0 \rightarrow S_{11}$	279	0.0133	HOMO-2→LUMO+1 (+63%)<br>HOMO-4→LUMO+1 (15%)
	$S_0 \rightarrow S_{12}$	271	0.0020	HOMO-8→LUMO (+32%)<br>HOMO-9→LUMO (+16%)<br>HOMO-7→LUMO (+10%)<br>HOMO-4→LUMO+1 (+10%)<br>HOMO-3→LUMO+1 (+8%)
	$S_0 \rightarrow S_{13}$	261	0.0276	HOMO-3→LUMO+1 (+42%)<br>HOMO-4→LUMO+1 (18%)<br>HOMO→LUMO+3 (17%)<br>HOMO-1→LUMO+2 (8%)<br>HOMO-2→LUMO+2 (+6%)
	$S_0 \rightarrow S_{14}$	251	0.1714	HOMO→LUMO+3 (+39%)<br>HOMO-2→LUMO+2 (+33%)
	$S_0 \rightarrow S_{15}$	248	0.0560	HOMO→LUMO+4 (+30%)<br>HOMO-1→LUMO+2 (25%)<br>HOMO-4→LUMO+2 (+12%)<br>HOMO-3→LUMO+1 (11%)<br>HOMO-3→LUMO+2 (+5%)

**3c**<br>***Cis***	$S_0 \rightarrow S_1$	482	0.0283	HOMO→LUMO (+98%)
	$S_0 \rightarrow S_2$	379	0.1681	HOMO→LUMO+1 (+90%)
	$S_0 \rightarrow S_3$	370	0.0345	HOMO-1→LUMO(+93%)
	$S_0 \rightarrow S_4$	356	0.0268	HOMO-2→LUMO(+65%)<br>HOMO-3→LUMO(+11%)<br>HOMO-4→LUMO(9%)<br>HOMO→LUMO+1(7%)
	$S_0 \rightarrow S_5$	340	0.1562	HOMO-3→LUMO(+70%)<br>HOMO-2→LUMO(14%)
	$S_0 \rightarrow S_6$	322	0.0109	HOMO-4→LUMO(+72%)<br>HOMO-3→LUMO(+6%)<br>HOMO-2→LUMO(+6%)
	$S_0 \rightarrow S_7$	311	0.0026	HOMO-6→LUMO(+32%)<br>HOMO-5→LUMO(+22%)<br>HOMO→LUMO+2(+10%)<br>HOMO-7→LUMO(+10%)
	$S_0 \rightarrow S_8$	307	0.0187	HOMO-1→LUMO+1(+50%)<br>HOMO→LUMO+2(19%)<br>HOMO-2→LUMO+1(+11%)
	$S_0 \rightarrow S_9$	304	0.0736	HOMO→LUMO+2(+61%)<br>HOMO-1→LUMO+1(+28%)
	$S_0 \rightarrow S_{10}$	296	0.2045	HOMO-2→LUMO+1(+52%)<br>HOMO-4→LUMO+1(12%)<br>HOMO-1→LUMO+1(11%)<br>HOMO-4→LUMO(7%)
	$S_0 \rightarrow S_{11}$	282	0.0232	HOMO-3→LUMO+1(+35%)<br>HOMO-2→LUMO+1(18%)<br>HOMO-4→LUMO+1(16%)<br>HOMO-9→LUMO(+14%)
	$S_0 \rightarrow S_{12}$	273	0.0377	HOMO-3→LUMO+1(+37%)<br>HOMO-9→LUMO(30%)<br>HOMO-1→LUMO+2(+7%)<br>HOMO-4→LUMO+1(+6%)
	$S_0 \rightarrow S_{13}$	267	0.0024	HOMO-4→LUMO+1(+43%)<br>HOMO-9→LUMO(+21%)<br>HOMO-2→LUMO+2(7%)
	$S_0 \rightarrow S_{14}$	257	0.0318	HOMO→LUMO+3(+36%)<br>HOMO-2→LUMO+2(34%)<br>HOMO-1→LUMO+2(8%)<br>HOMO-4→LUMO+2(+5%)
	$S_0 \rightarrow S_{15}$	256	0.0924	HOMO-5→LUMO(+45%)<br>HOMO-1→LUMO+2(17%)<br>HOMO-6→LUMO(16%)<br>HOMO-3→LUMO+2(6%)
	$S_0 \rightarrow S_{16}$	252	0.0623	HOMO-1→LUMO+2 (+53%)<br>HOMO→LUMO+3 (+21%)<br>HOMO-5→LUMO (+11%)
**3d**<br>***Trans***	$S_0 \rightarrow S_1$	476	0.1078	HOMO→LUMO (+99%)

	$S_0 \rightarrow S_2$	378	0.0009	HOMO-1→LUMO (+99%)
	$S_0 \rightarrow S_3$	371	1.0846	HOMO→LUMO+1(+97%)
	$S_0 \rightarrow S_4$	347	0.1185	HOMO-2→LUMO (+71%)<br>HOMO-3→LUMO (9%)<br>HOMO-4→LUMO (+6%)
	$S_0 \rightarrow S_5$	331	0.0181	HOMO-4→LUMO (+80%)
	$S_0 \rightarrow S_6$	327	0.0510	HOMO-3→LUMO (+67%)<br>HOMO-1→LUMO+1(+11%)<br>HOMO-2→LUMO (+8%)
	$S_0 \rightarrow S_7$	326	0.0074	HOMO-1→LUMO +1(+83%)<br>HOMO-3→LUMO (9%)
	$S_0 \rightarrow S_8$	316	0.0960	HOMO→LUMO+2(+81%)<br>HOMO-6→LUMO (5%)
	$S_0 \rightarrow S_9$	308	0.0774	HOMO-6→LUMO (+41%)<br>HOMO-5→LUMO (16%)<br>HOMO-2→LUMO (9%)<br>HOMO→LUMO+2(+8%)<br>HOMO-7→LUMO (8%)
	$S_0 \rightarrow S_{10}$	292	0.0380	HOMO-2→LUMO+1 (+46%)<br>HOMO-4→LUMO+1 (+31%)
	$S_0 \rightarrow S_{11}$	284	0.0609	HOMO-4→LUMO+1 (+46%)<br>HOMO-2→LUMO+1 (35%)
	$S_0 \rightarrow S_{12}$	274	0.0976	HOMO-3→LUMO+1 (+65%)<br>HOMO-8→LUMO (7%)<br>HOMO-2→LUMO+2 (6%)
	$S_0 \rightarrow S_{13}$	271	0.0231	HOMO-8→LUMO (+23%)<br>HOMO-4→LUMO+1 (+15%)<br>HOMO-3→LUMO+1 (+14%)<br>HOMO-9→LUMO (+14%)<br>HOMO-7→LUMO (+9%)
	$S_0 \rightarrow S_{14}$	266	0.0012	HOMO-1→LUMO+2 (+94%)
	$S_0 \rightarrow S_{15}$	257	0.0996	HOMO-5→LUMO (+58%)<br>HOMO-2→LUMO+2 (16%)<br>HOMO-6→LUMO (+9%)
	$S_0 \rightarrow S_{16}$	252	0.1033	HOMO→LUMO+3(+40%)<br>HOMO-2→LUMO+2 (+38%)<br>HOMO-5→LUMO (+10%)
**3d**<br>***Cis***	$S_0 \rightarrow S_1$	467	0.0699	HOMO→LUMO (+99%)
	$S_0 \rightarrow S_2$	397	0.0114	HOMO-1→LUMO (+99%)
	$S_0 \rightarrow S_3$	361	0.3547	HOMO→LUMO+1 (+93%)
	$S_0 \rightarrow S_4$	350	0.1675	HOMO-2→LUMO (+79%)<br>HOMO→LUMO+1 (5%)
	$S_0 \rightarrow S_5$	332	0.0245	HOMO-3→LUMO (+53%)<br>HOMO-4→LUMO (+34%)<br>HOMO-2→LUMO (+6%)
	$S_0 \rightarrow S_6$	328	0.0478	HOMO-4→LUMO (+48%)<br>HOMO-3→LUMO (30%)<br>HOMO-6→LUMO (10%)

	$S_0 \rightarrow S_7$	324	0.0047	HOMO-1→LUMO+1 (+91%)
	$S_0 \rightarrow S_8$	310	0.0328	HOMO→LUMO+2 (+67%)<br>HOMO-6→LUMO (+16%)
	$S_0 \rightarrow S_9$	307	0.0411	HOMO-6→LUMO (+39%)<br>HOMO→LUMO+2 (25%)<br>HOMO-7→LUMO (7%)<br>HOMO-3→LUMO (6%)<br>HOMO-2→LUMO (+5%)
	$S_0 \rightarrow S_{10}$	288	0.0721	HOMO-2→LUMO+1 (+53%)<br>HOMO-4→LUMO+1 (18%)<br>HOMO-8→LUMO (+8%)<br>HOMO-3→LUMO+1 (5%)
	$S_0 \rightarrow S_{11}$	281	0.0850	HOMO-4→LUMO+1 (+42%)<br>HOMO-2→LUMO+1 (+30%)<br>HOMO-8→LUMO (13%)
	$S_0 \rightarrow S_{12}$	277	0.1033	HOMO-1→LUMO+2 (+86%)
	$S_0 \rightarrow S_{13}$	272	0.0160	HOMO-3→LUMO+1 (+51%)<br>HOMO-8→LUMO (+14%)<br>HOMO-2→LUMO+2 (+9%)<br>HOMO-1→LUMO+2 (+6%)
	$S_0 \rightarrow S_{14}$	269	0.0101	HOMO-4→LUMO+1 (+27%)<br>HOMO-3→LUMO+1 (16%)<br>HOMO-8→LUMO (+15%)<br>HOMO-5→LUMO (+14%)
	$S_0 \rightarrow S_{15}$	261	0.0355	HOMO-5→LUMO (+63%)<br>HOMO-8→LUMO (7%)<br>HOMO-3→LUMO+1 (+6%)
	$S_0 \rightarrow S_{16}$	251	0.2891	HOMO-2→LUMO+2 (+48%)<br>HOMO→LUMO+3 (32%)<br>HOMO-5→LUMO (+6%)

Table S3. Spectroscopic data of the $S_1 \rightarrow S_0$ transitions for the **3a** calculated at the TDDFT/B3LYP/6-31G(d,p) level of theory taking into account solvent effects (solvent – dimethylsulfoxide).

Compound	Conformer	Transition	λ_{em}, nm	*E*, eV	*f*
**3a**	1	$S_1 \rightarrow S_0$	527	2.35	0.5685
	2	$S_1 \rightarrow S_0$	521	2.38	0.8771
	3	$S_1 \rightarrow S_0$	568	2.18	0.1271

	4	$S_1 \rightarrow S_0$	539	2.30	0.1578
	5	$S_1 \rightarrow S_0$	536	2.31	0.2937
	6	$S_1 \rightarrow S_0$	525	2.36	0.5939

Part II. Experimental data

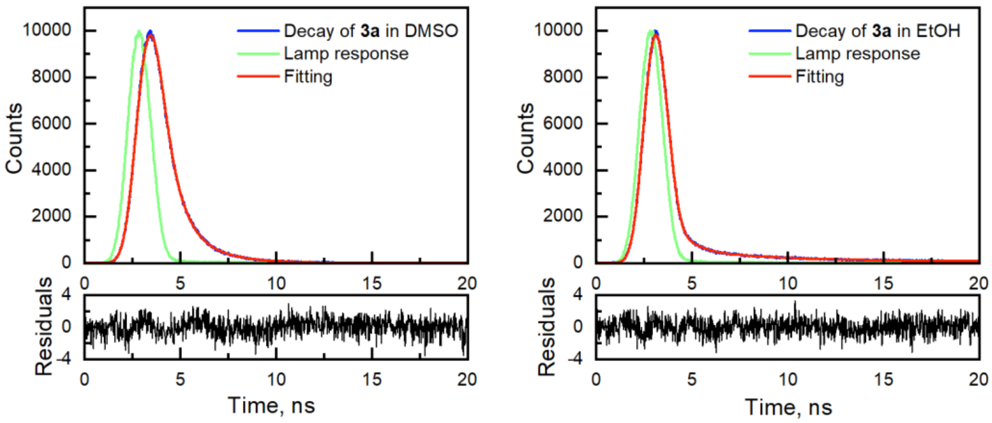

Figure S7. The fluorescence decay profiles of compound **3a** in different solvents with excitation at 376.2 nm. Lamp response was determined using Ludox. Residuals are shown versus time.

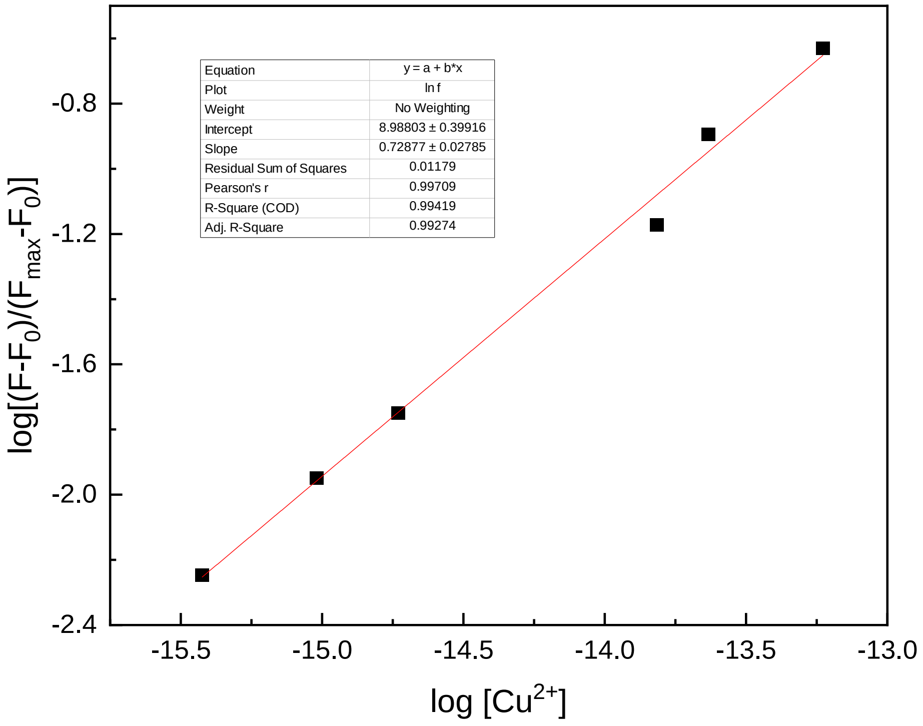

Figure S8. Modified logarithmic-type Stern-Volmer plot for **3a** in the presence of various concentrations of Cu^{2+}.

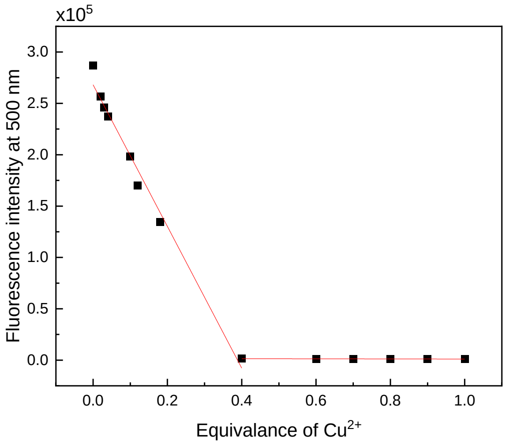

Figure S9. Job's plots for **3a** in the presence of various concentrations of Cu^{2+}.

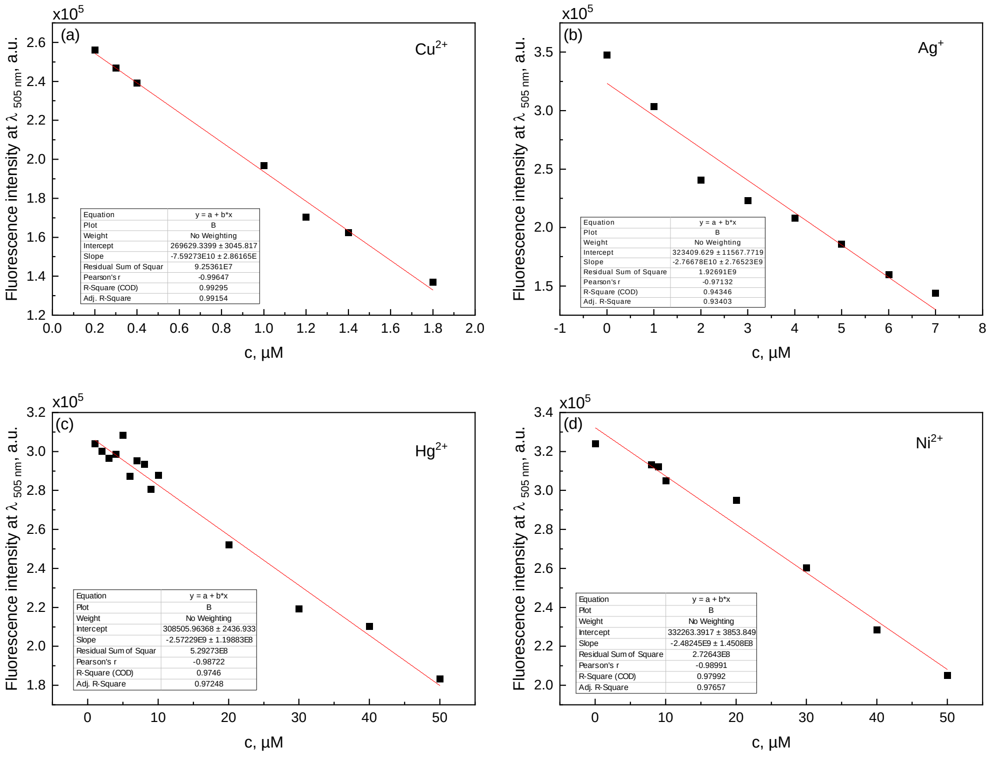

Figure S10. The calibration curves of fluorescence intensity at 505 nm as a function of metal ion concentration for derivative **3a** (a) Cu^{2+}, (b) Ag^{+}, (c) Hg^{2+}, (d) Ni^{2+}.

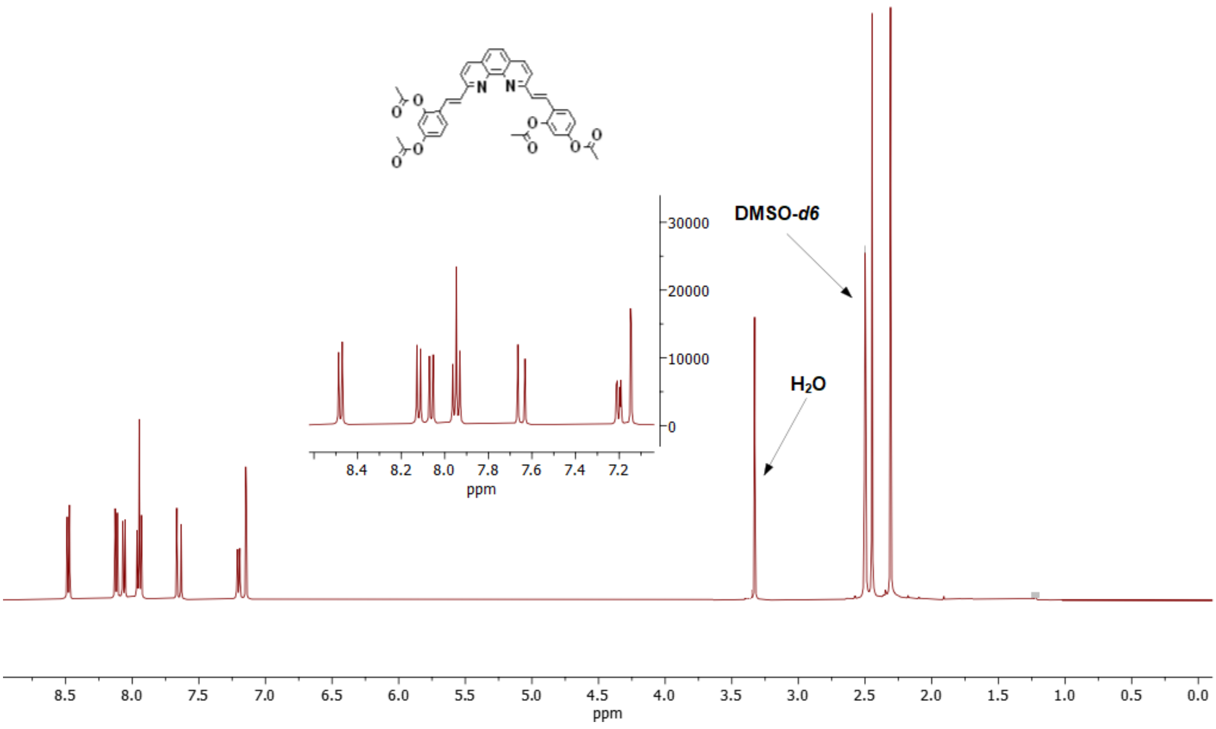

Fig. S11a. ¹H-NMR (DMSO-*d6*; 500.2 MHz) spectrum of **2a**.

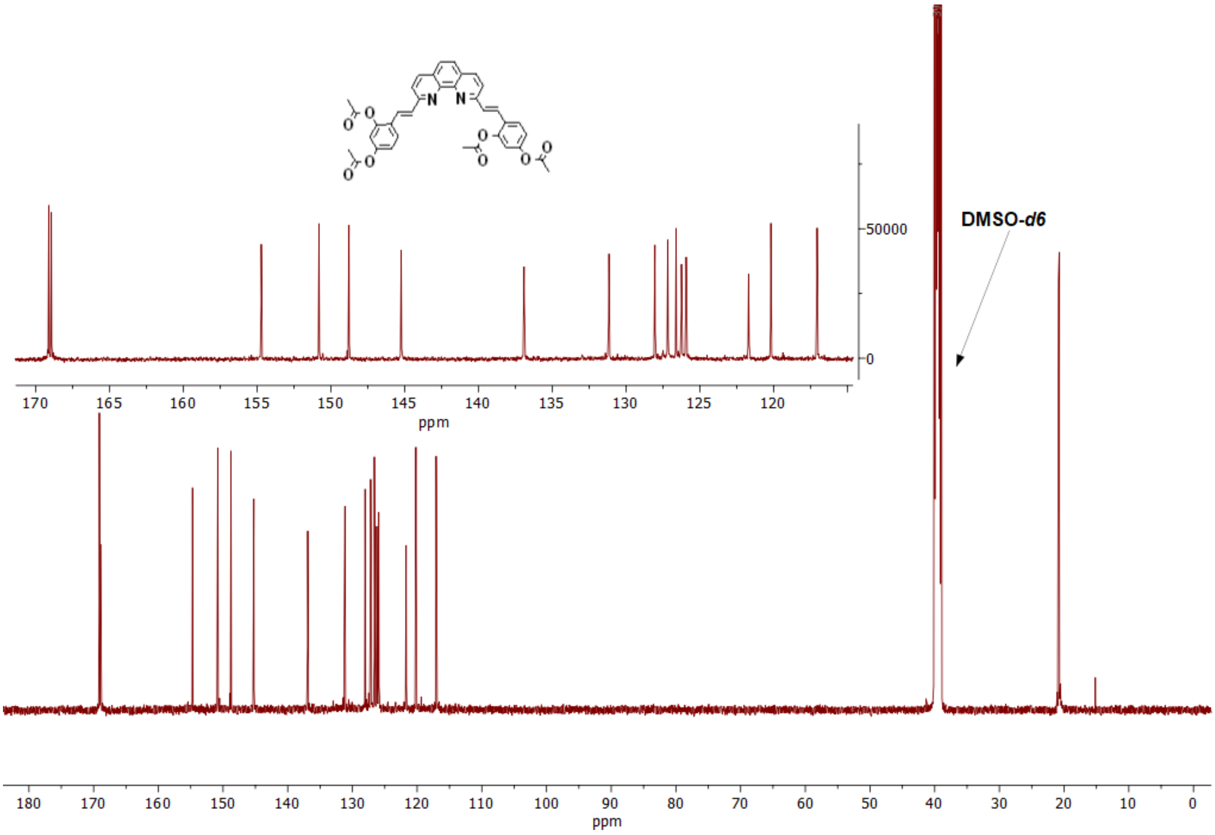

Fig. S11b. ^{13}C{^{1}H}-NMR (DMSO-*d6*; 125.8 MHz) spectrum of **2a**.

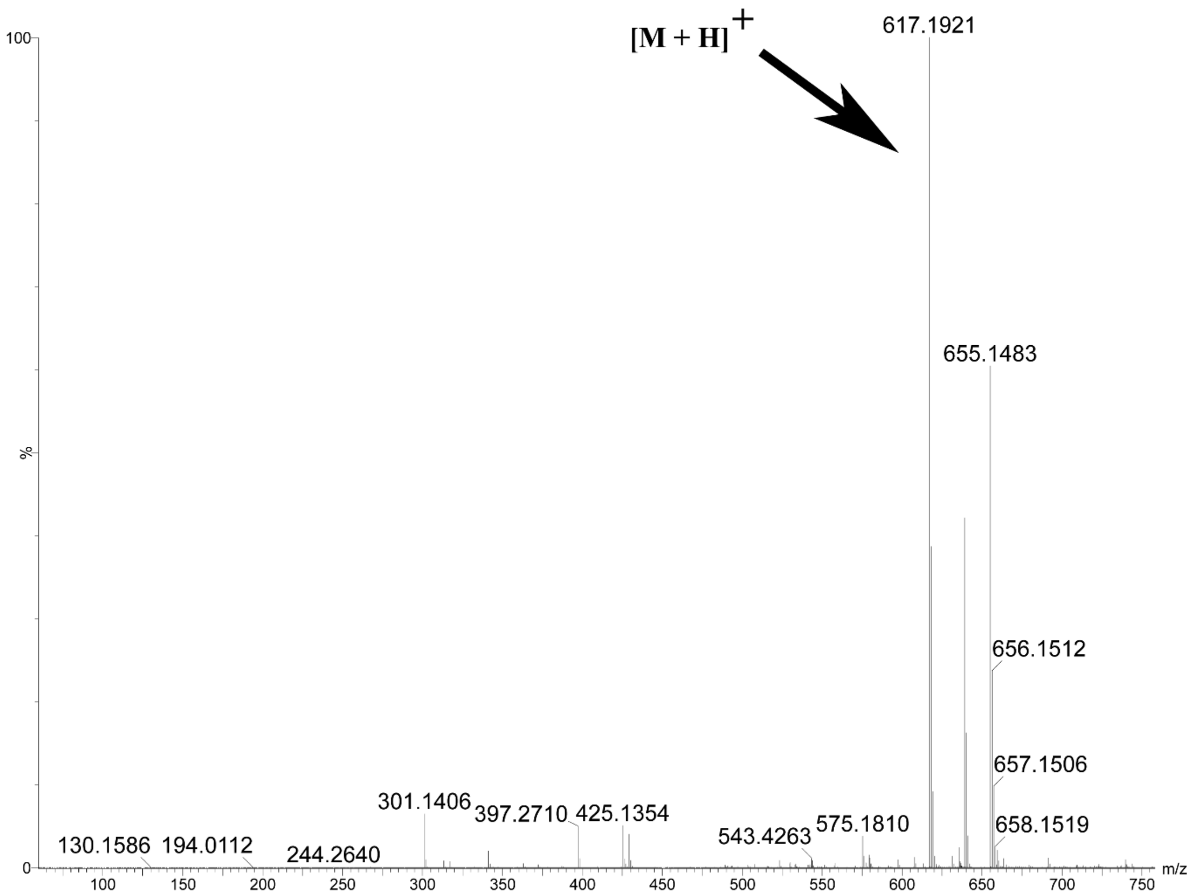

Fig. S11c. MS spectrum of the molecule **2a**.

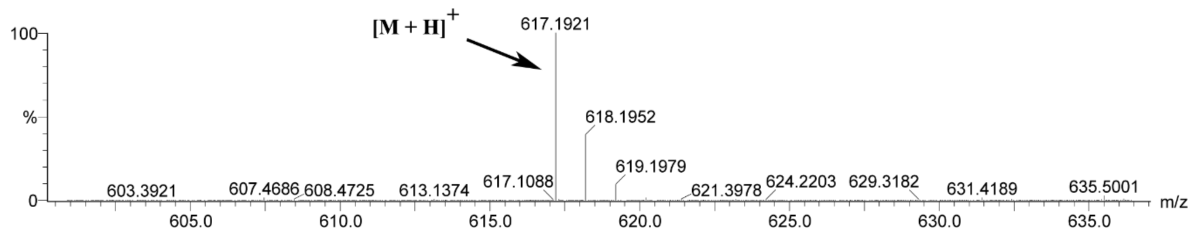

Fig. S11d. HRMS spectrum of the molecule **2a**.

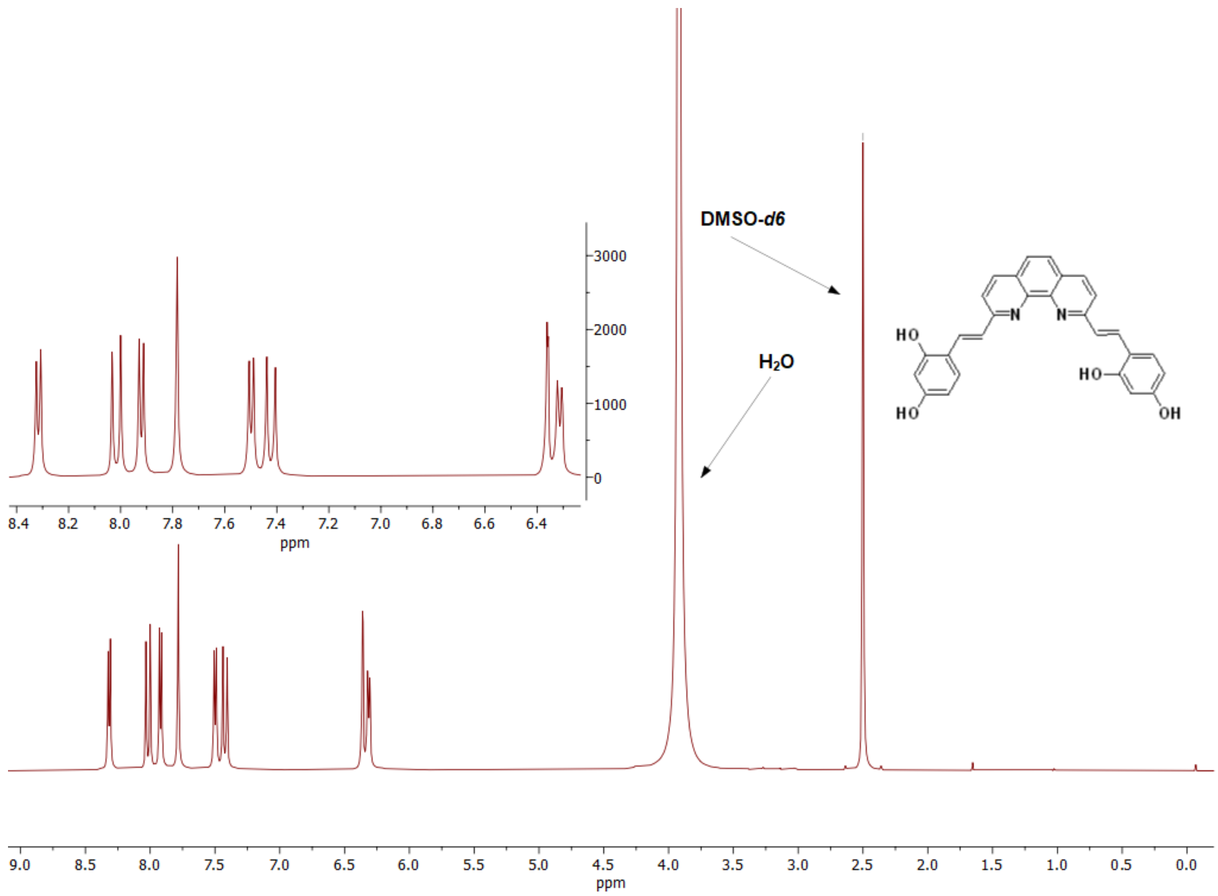

Fig. S12a. ^{1}H-NMR (DMSO-*d6*/KOD; 500.2 MHz) spectrum of **3a**.

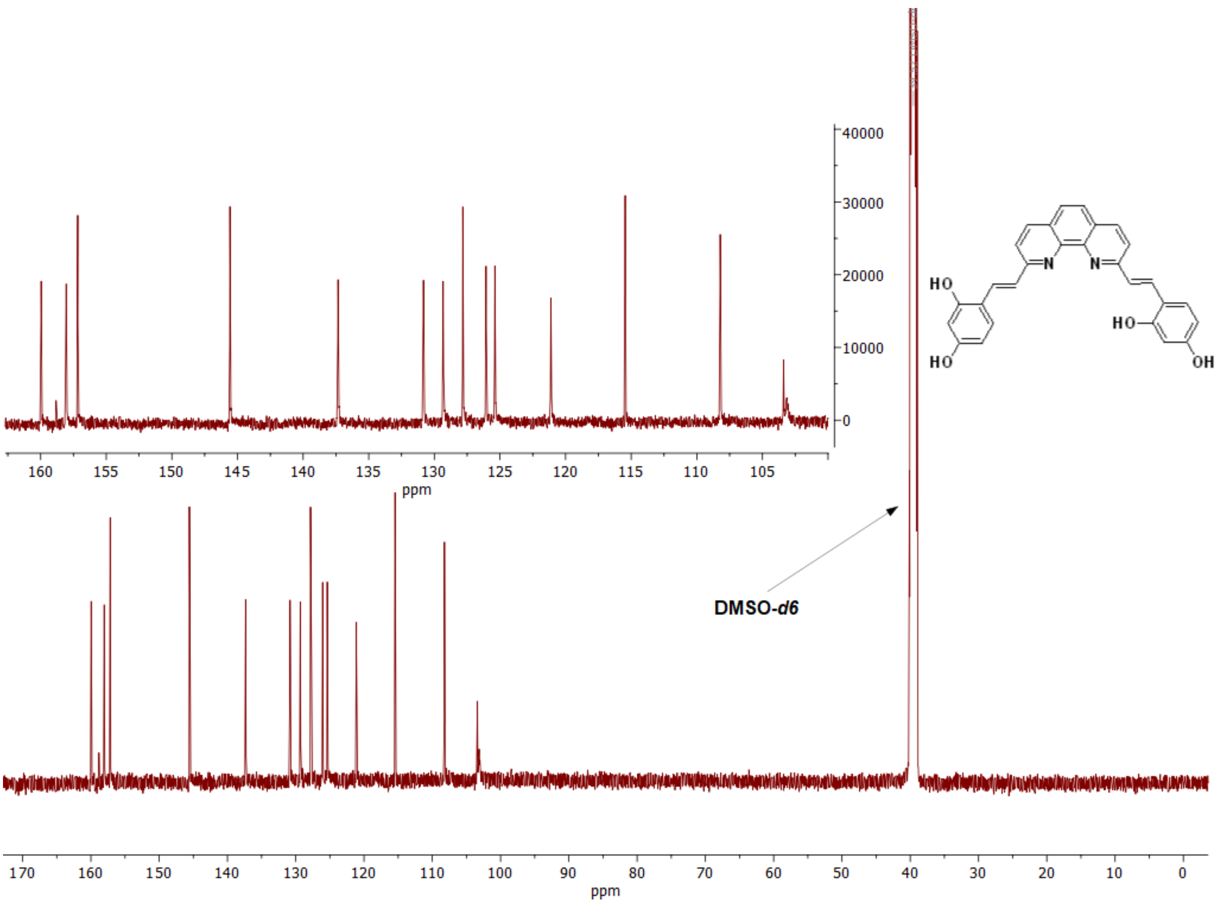

Fig. S12b. $^{13}C\{^{1}H\}$-NMR (DMSO-*d6*/KOD; 125.8 MHz) spectrum of **3a**.

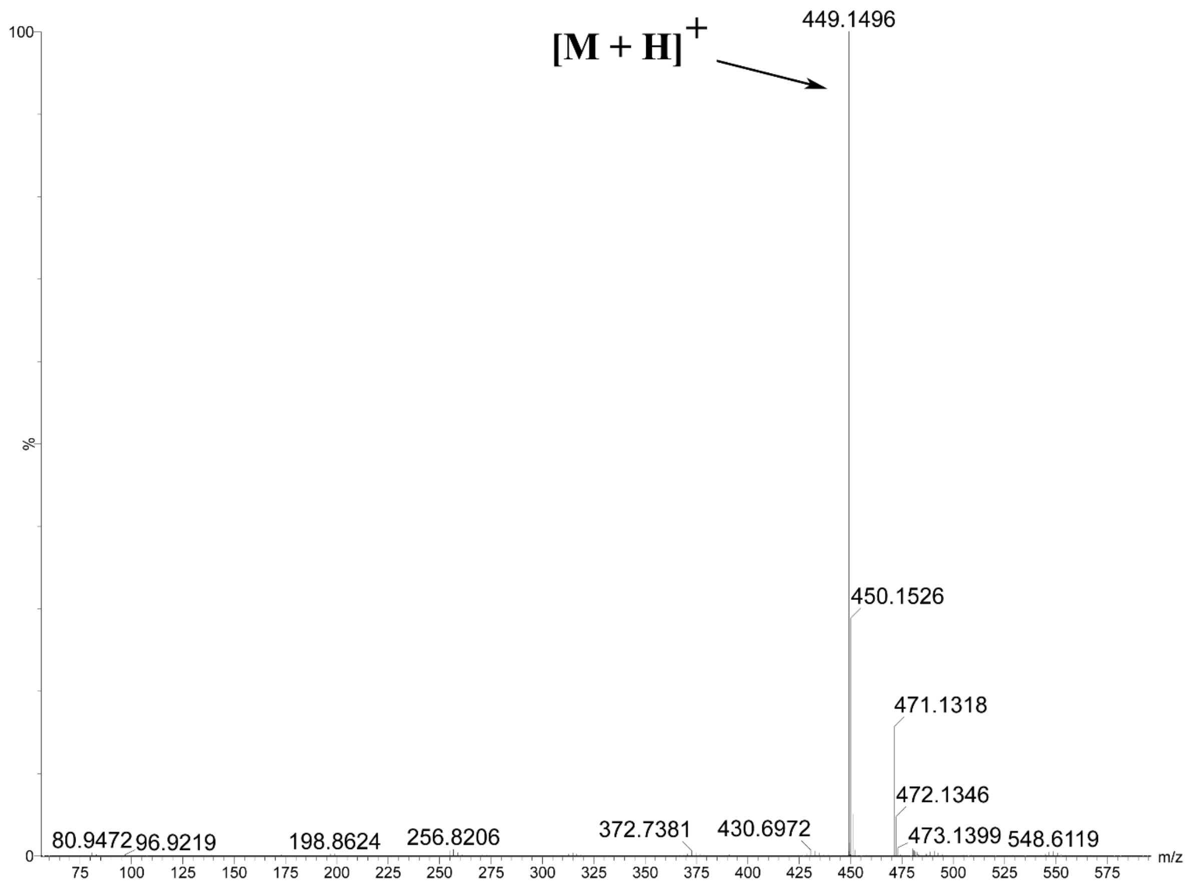

Fig. S12c. MS spectrum of the molecule **3a**.

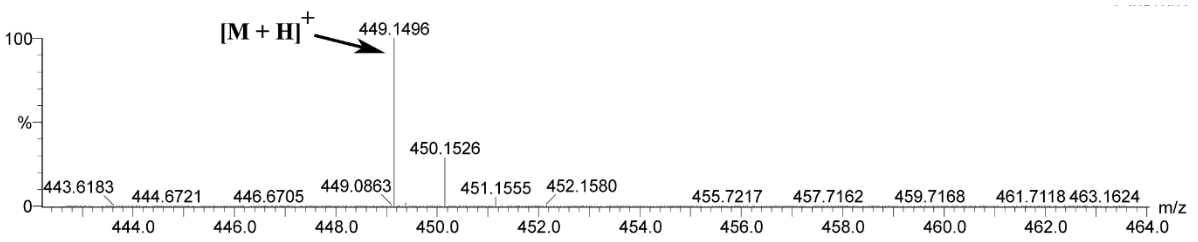

Fig. S12d. HRMS spectrum of the molecule **3a**.

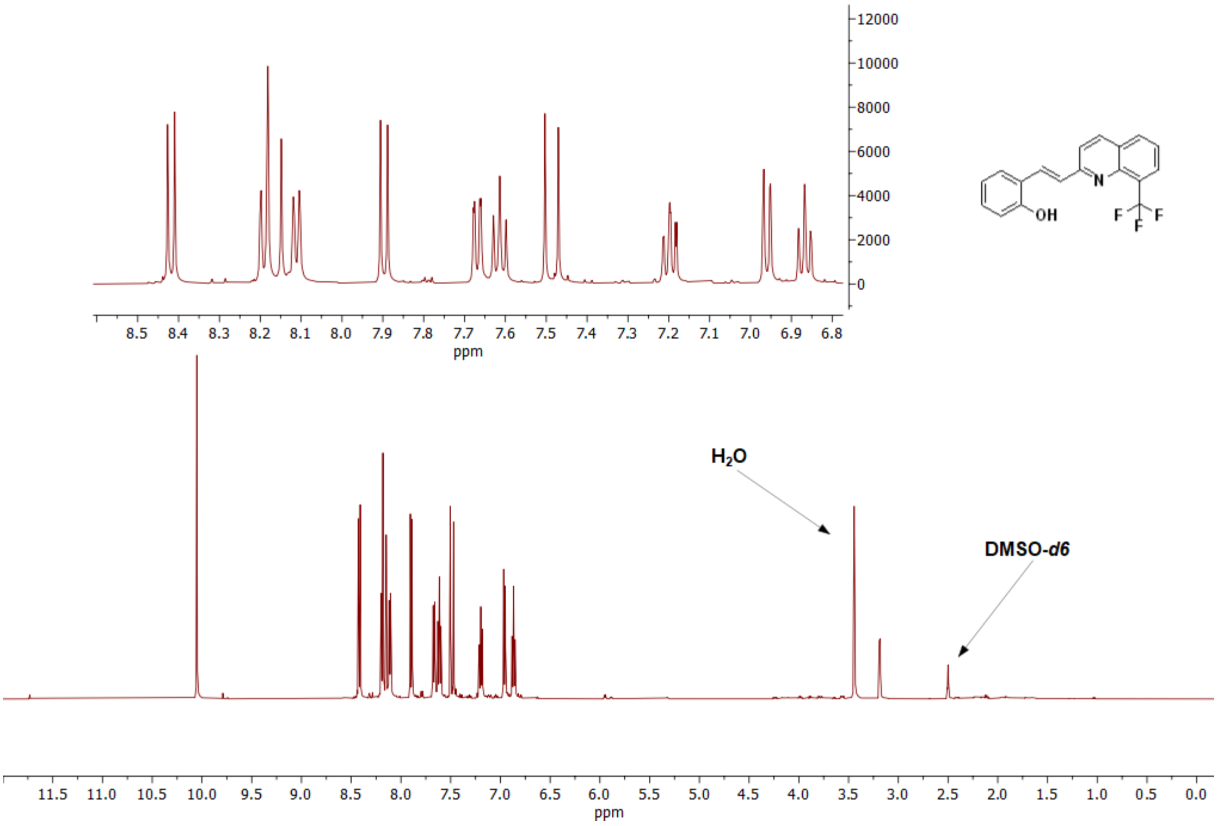

Fig. S13a. ^{1}H-NMR (DMSO-*d6*; 400.2 MHz) spectrum of **3b**.

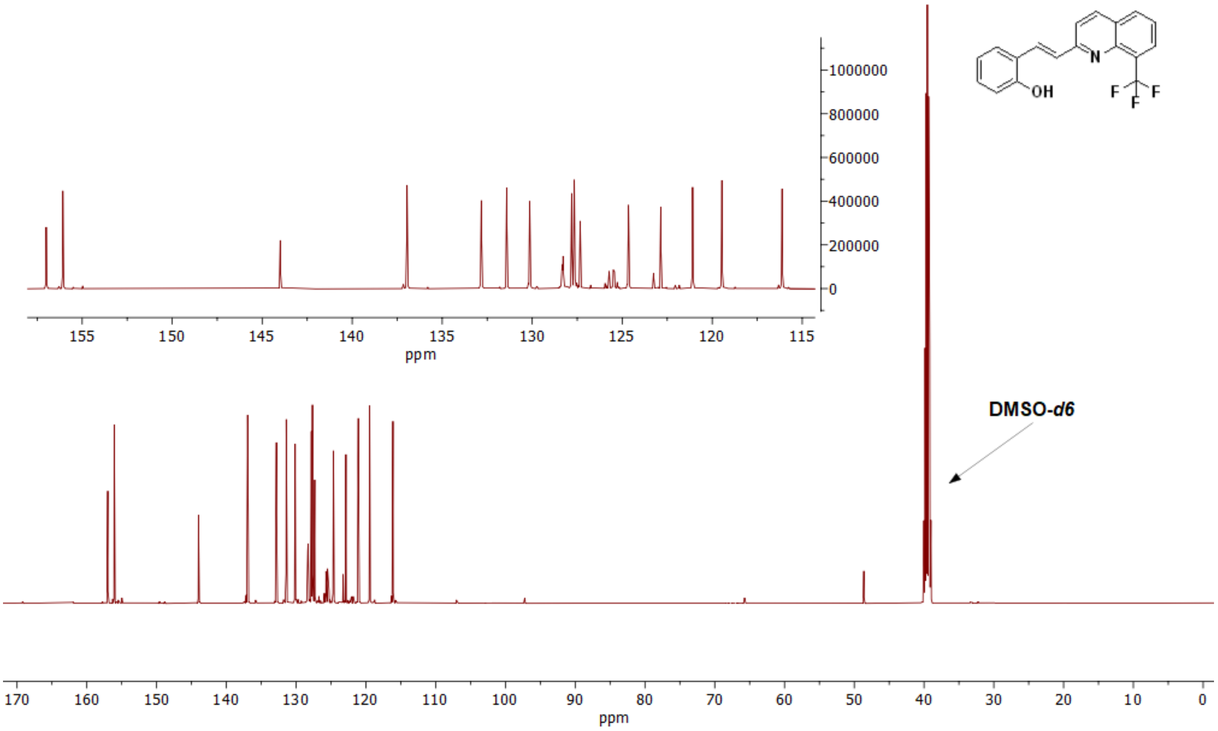

Fig. S13b. ^{13}C{^{1}H}-NMR (DMSO-*d6*; 100.6 MHz) spectrum of **3b**.

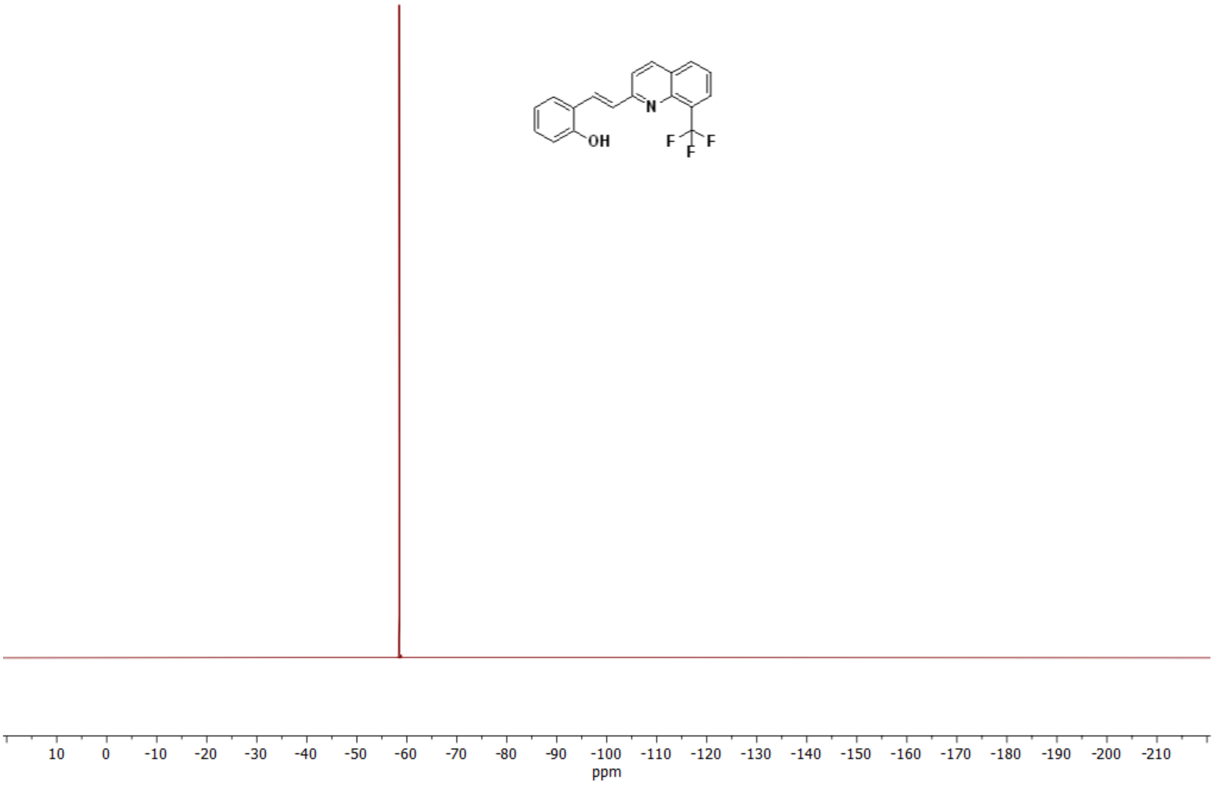

Fig. S13c. ^{19}F-NMR (DMSO-*d6*; 470.6 MHz) spectrum of **3b**.

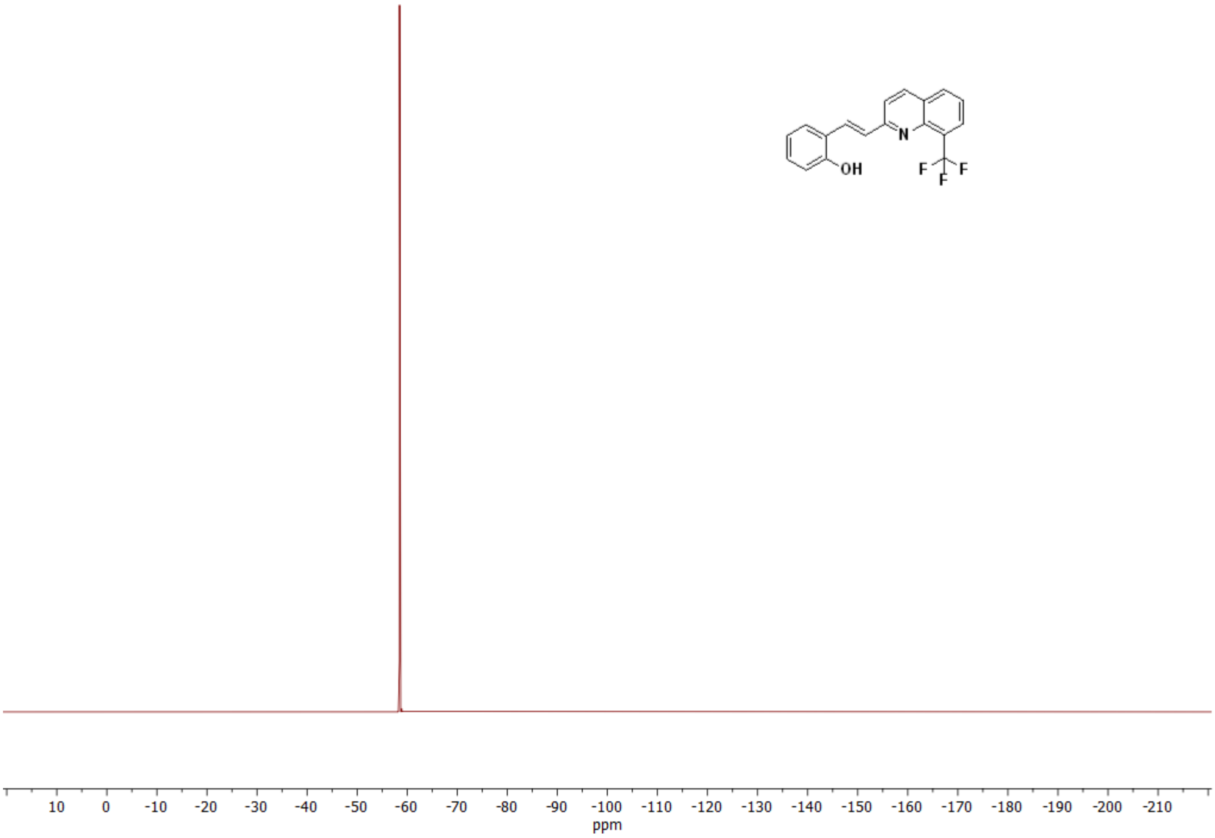

Fig. S13d. ^{19}F{^{1}H}-NMR (DMSO-*d6*; 470.6 MHz) spectrum of **3b**.

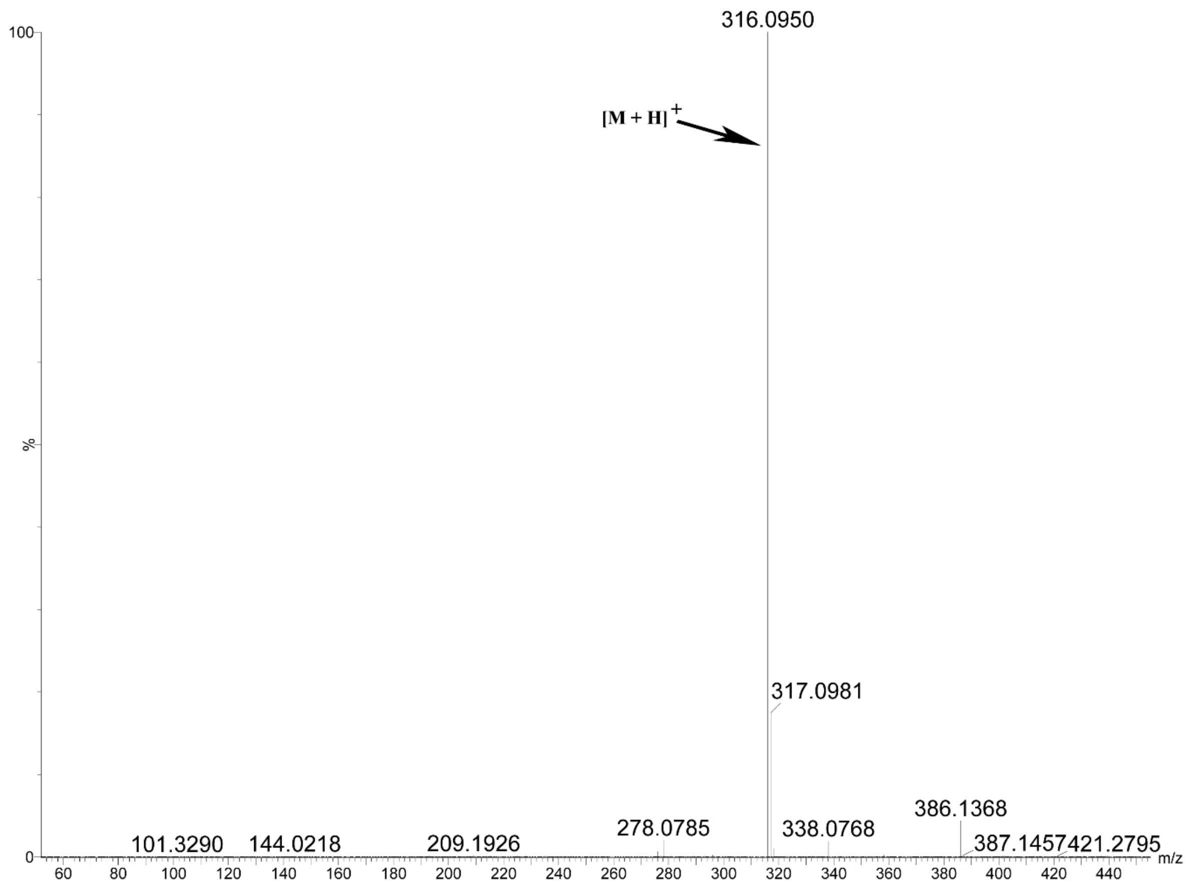

Fig. S13e. MS spectrum of the molecule **3b**.

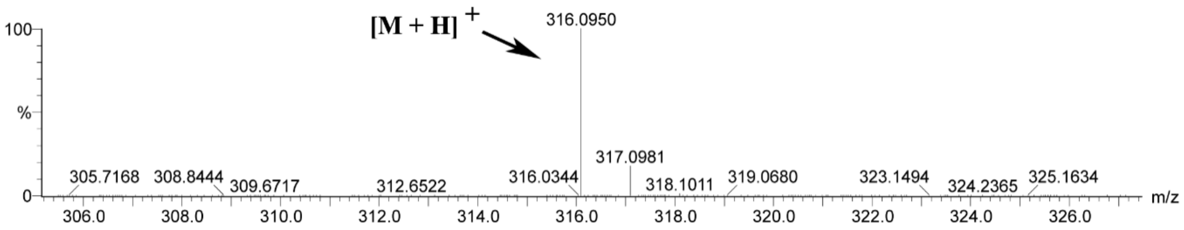

Fig. S13f. HRMS spectrum of the molecule **3b**.

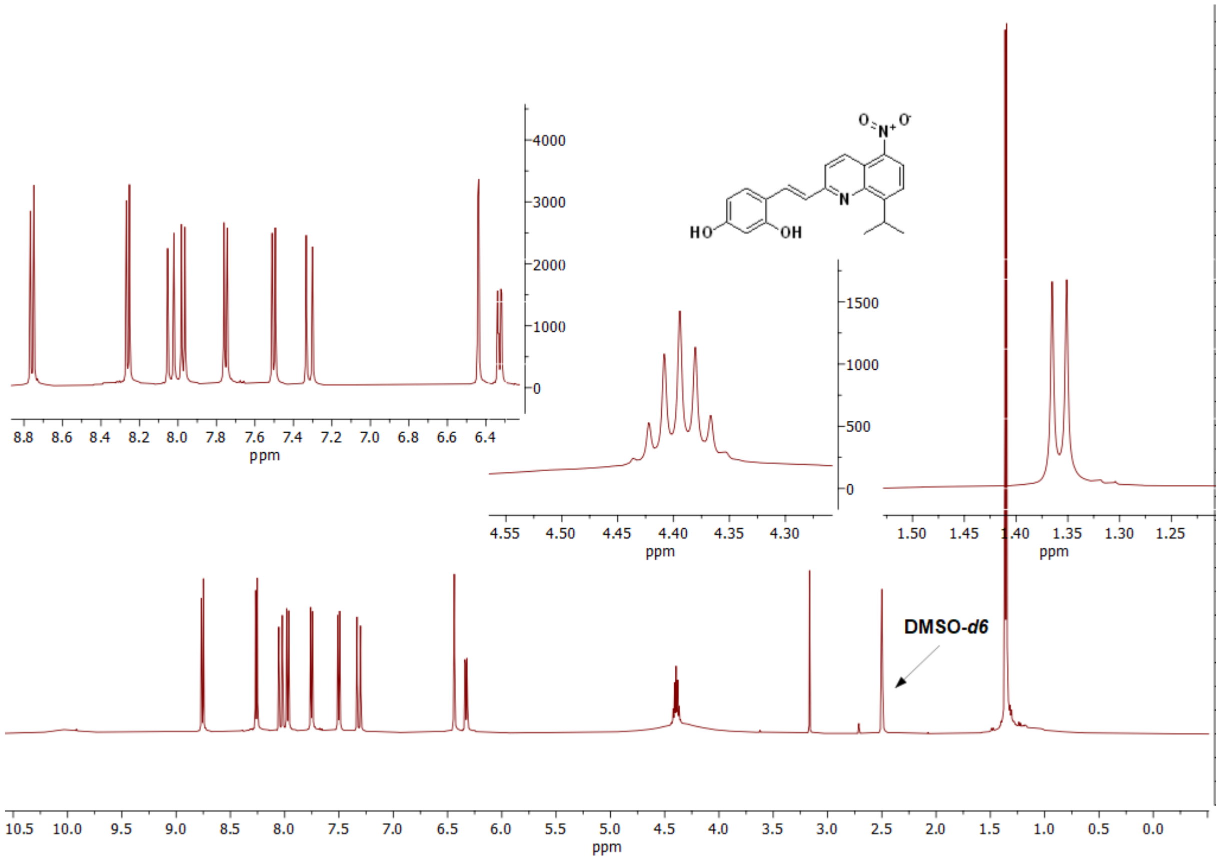

Fig. S14a. ^{1}H-NMR (DMSO-*d6*; 500.2 MHz) spectrum of **3c**.

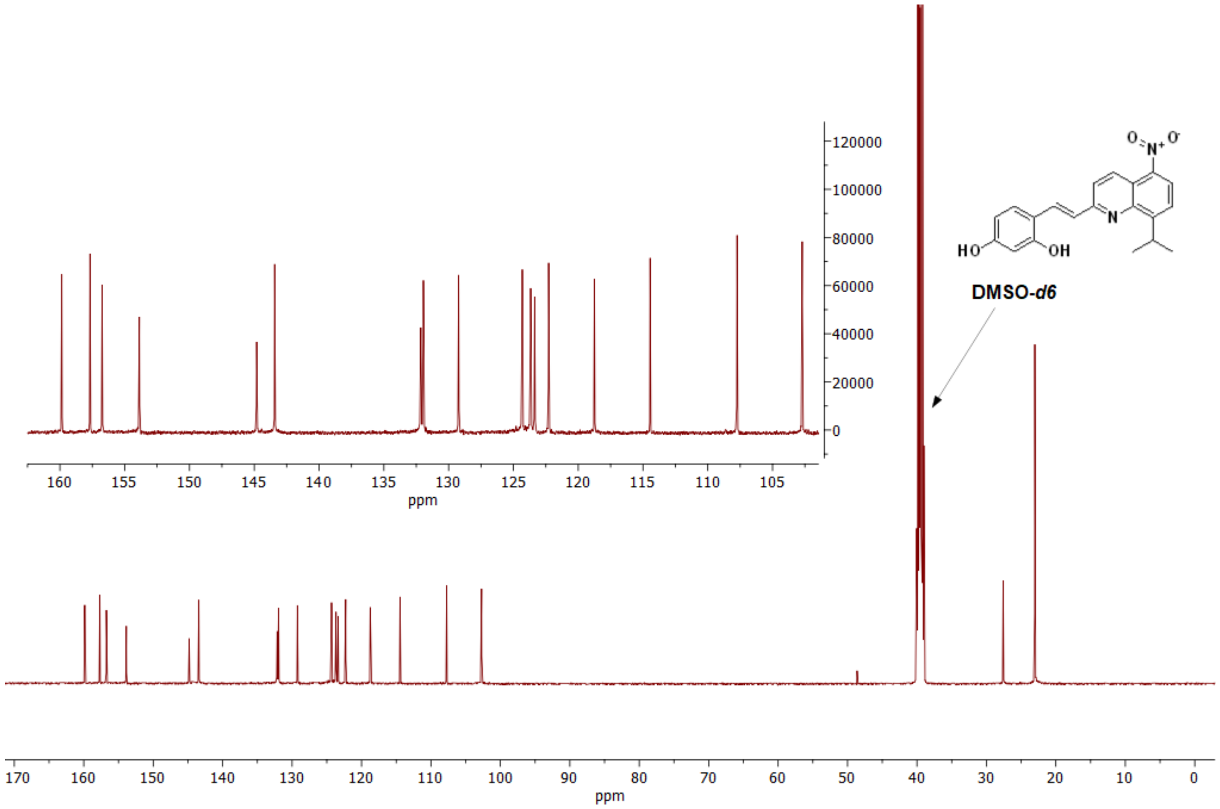

Fig. S14b. ^{13}C{^{1}H}-NMR (DMSO-*d6*; 125.8 MHz) spectrum of **3c**.

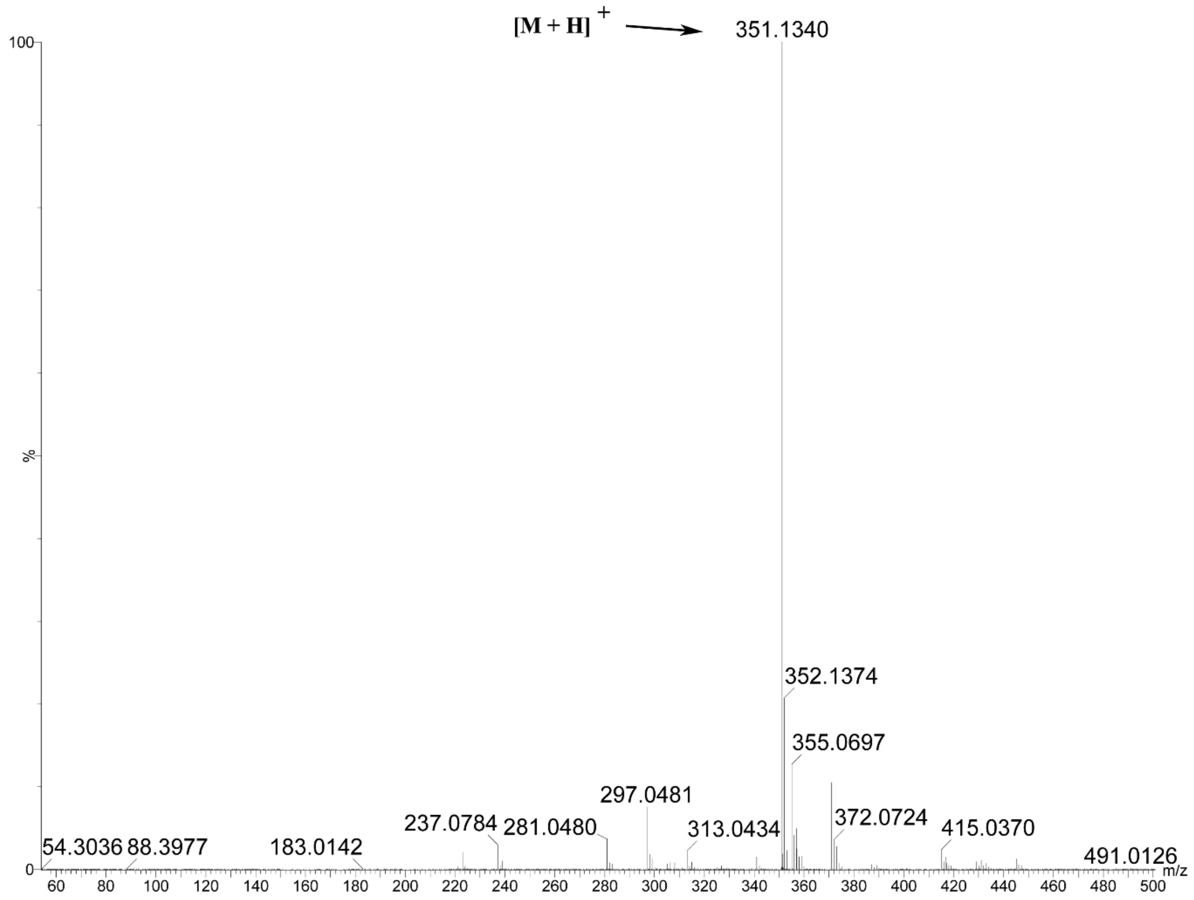

Fig. S14c. MS spectrum of the molecule **3c**.

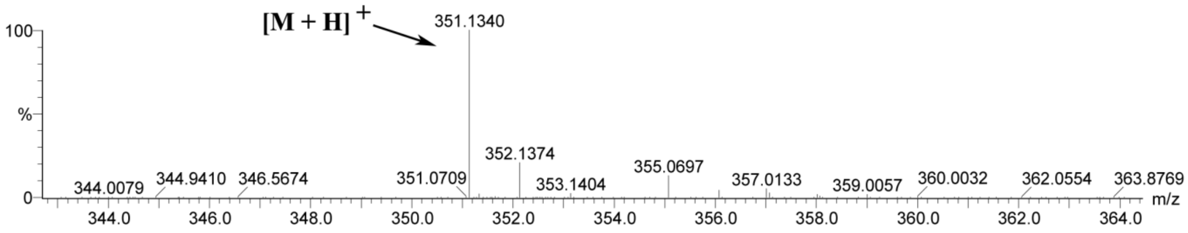

Fig. S14d. HRMS spectrum of the molecule **3c**.

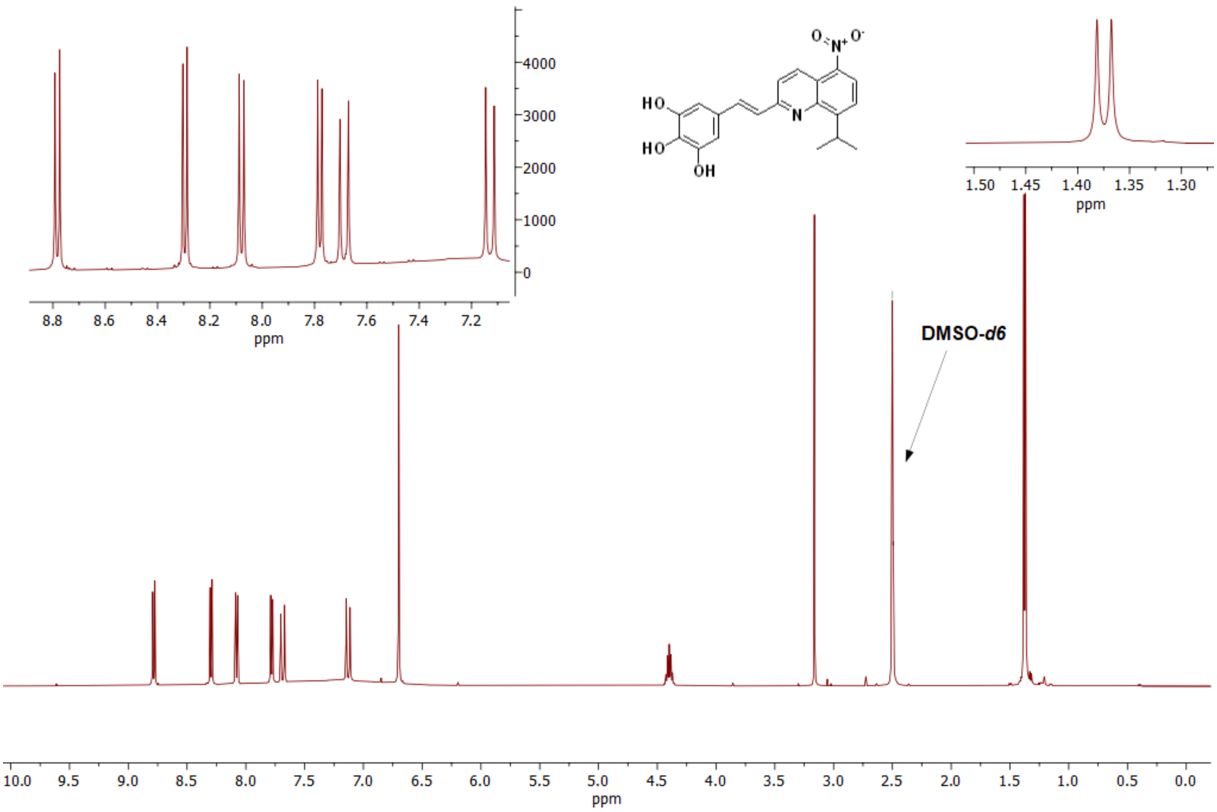

Fig. S15a. ^{1}H-NMR (DMSO-*d6*; 500.2 MHz) spectrum of **3d**.

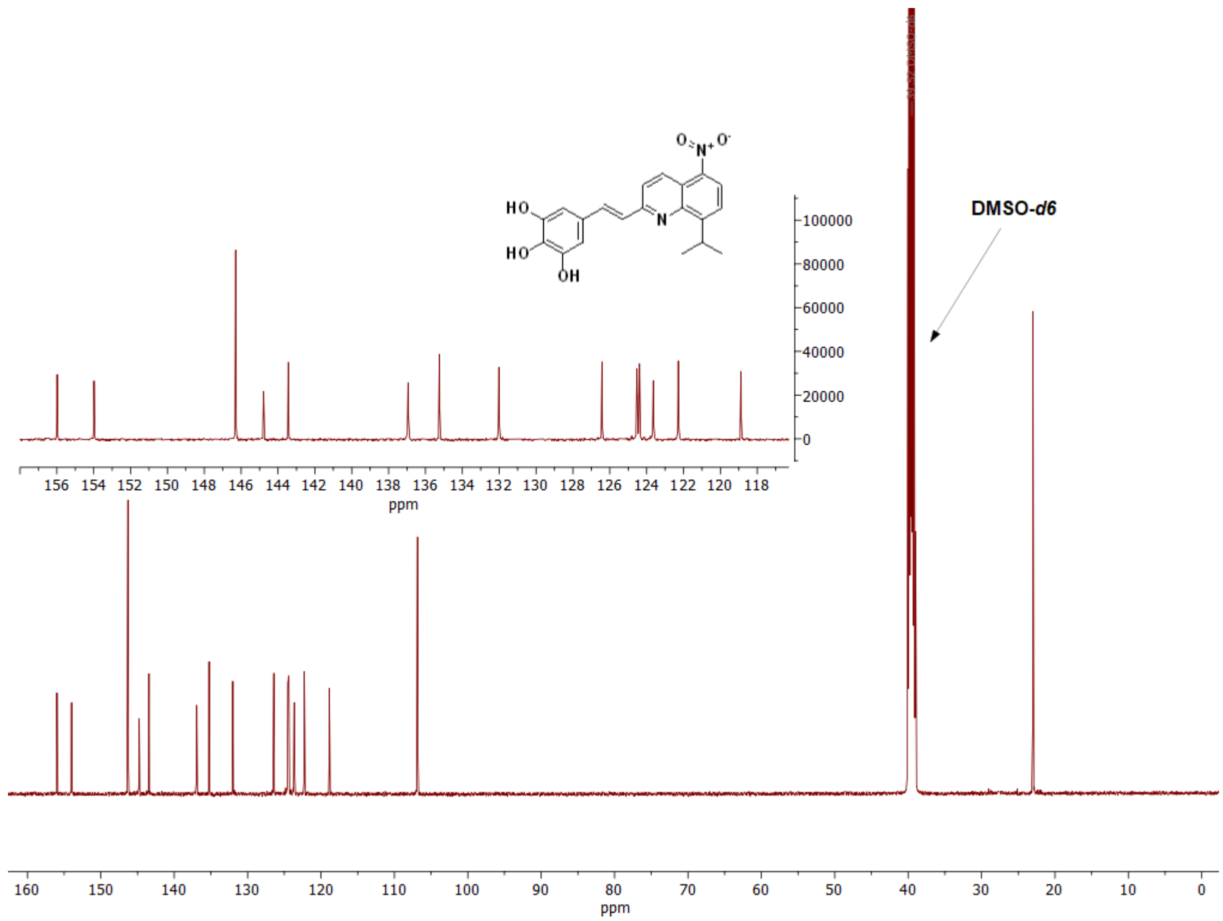

Fig. S15b. $^{13}C\{^1H\}$-NMR (DMSO-*d6*; 125.8 MHz) spectrum of **3d**.

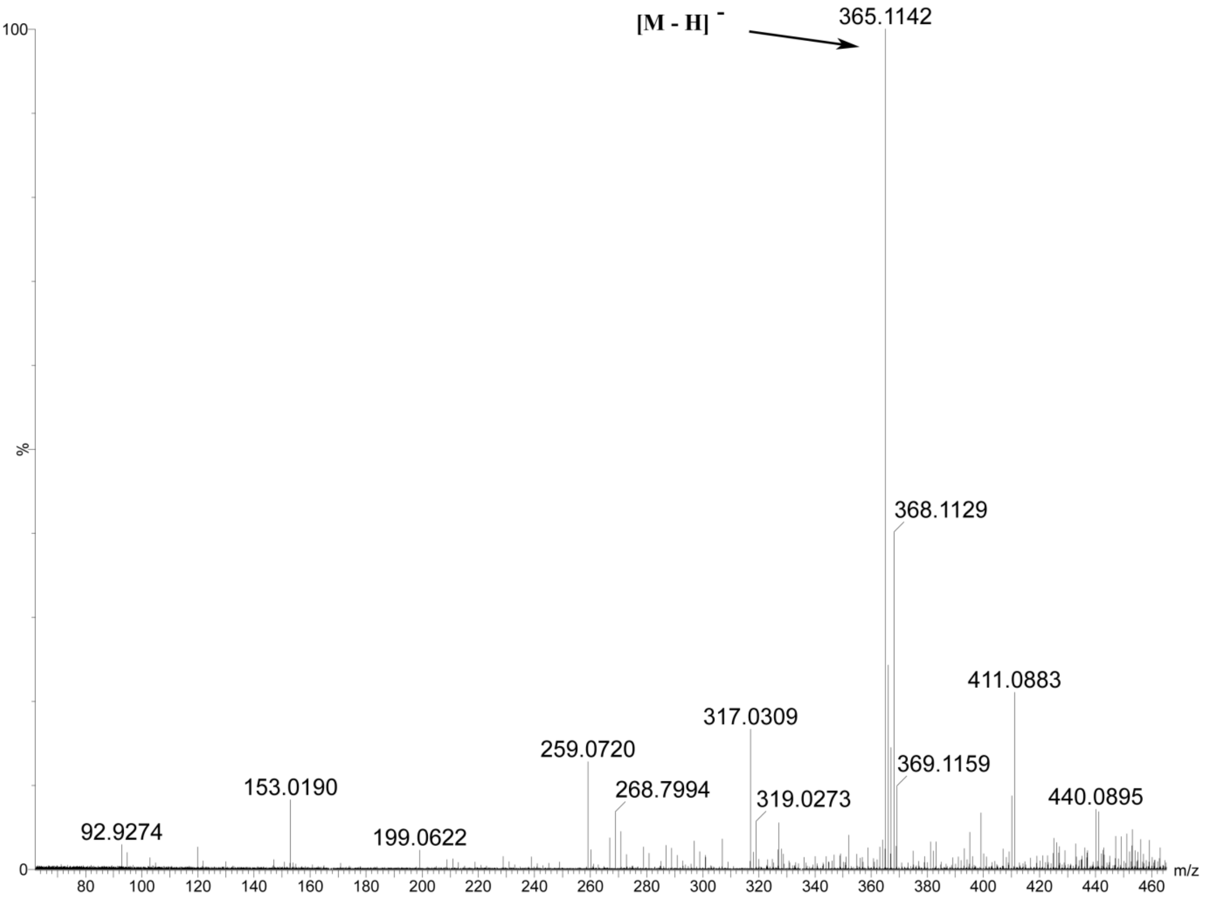

Fig. S15c. MS spectrum of the molecule **3d**.

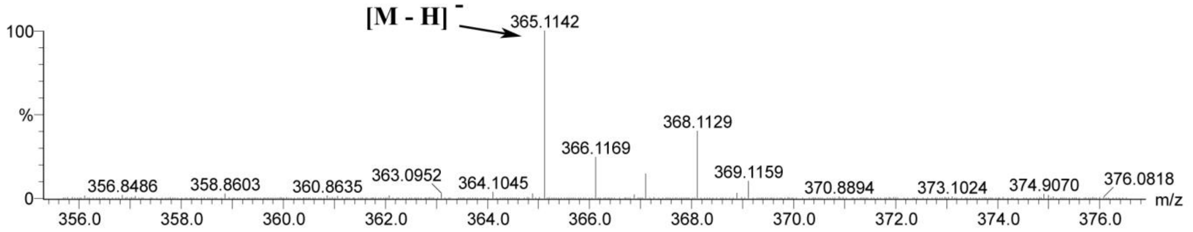

Fig. S15d. HRMS spectrum of the molecule **3d**.

Table S4. Crystal data and structure refinement details for **2a**.

CCDC number	2355007
Empirical formula	$C_{36}H_{28}N_2O_8$
Formula weight	616.60
Temperature [K]	150(1)
Wavelength [Å]	1.54184
Crystal system	Monoclinic
Space group	P 2_1/c
Unit cell dimensions<br>a [Å]<br>b [Å]<br>c [Å]<br>β [°]	 9.5864(6)<br>36.0174(19)<br>8.6135(5)<br>91.514(6)
V [Å^3]	2973.0(3)
Z	4
Density (calculated) [Mg/m^3]	1.378
Absorption coefficient [mm^{-1}]	0.812
F(000)	1288
Theta range for data collection [°]	4.614 to 73.595
Index ranges	$-11 \leq h \leq 10$<br>$-39 \leq k \leq 44$<br>$-10 \leq l \leq 10$
Reflections collected	19231
Independent reflections	5813 [R(int) = 0.0983]
Completeness to theta = 67.684° [%]	98.2
Data / restraints / parameters	5813 / 0 / 423
Goodness-of-fit on F^2	1.052
Final R indices [I>2σ(I)]	R1 = 0.0869, wR2 = 0.2055
R indices (all data)	R1 = 0.1428, wR2 = 0.2428
Largest diff. peak and hole [eÅ^{-3}]	0.403 and -0.343

Table S5. The optimized Cartesian coordinates of the *trans* rotamer **1** of **3a** calculated at the DFT/B3LYP-GD3/6-311++G(d) level of theory

Center Number	*Atomic Number*	*Coordinates (Angstroms)* X	Y	Z
1	6	-4.579891	-2.826170	-0.119794
2	6	-3.367172	-3.472395	-0.094397
3	6	-2.174821	-2.703889	-0.056241
4	7	-2.199227	-1.373865	-0.044906
5	6	-3.374719	-0.730953	-0.075770
6	6	-4.623041	-1.415646	-0.111762
7	6	-5.851172	-0.681324	-0.140552
8	6	-5.851367	0.679655	-0.140606
9	6	-4.623452	1.414336	-0.111869
10	6	-3.374929	0.730009	-0.075816
11	6	-4.580710	2.824872	-0.120008
12	6	-3.368178	3.471452	-0.094633
13	6	-2.175606	2.703295	-0.056375
14	7	-2.199627	1.373265	-0.044963
15	1	-5.507553	-3.388689	-0.148187
16	1	-3.312961	-4.555239	-0.104012
17	1	-6.785099	-1.233134	-0.165026
18	1	-6.785456	1.231193	-0.165126
19	1	-5.508532	3.387123	-0.148463
20	1	-3.314283	4.554311	-0.104330
21	6	-0.870498	-3.375474	-0.032606
22	6	0.301881	-2.703402	-0.056742
23	6	1.657238	-3.237899	-0.047080
24	6	2.023213	-4.467609	0.545651
25	6	3.335323	-4.931946	0.486378
26	6	4.314795	-4.176165	-0.152513
27	6	3.996947	-2.933723	-0.710499
28	6	2.691329	-2.483280	-0.636284
29	1	2.438912	-1.520222	-1.067081
30	1	0.221487	-1.620878	-0.122514
31	1	-0.899657	-4.460761	-0.070957
32	8	1.140178	-5.251711	1.227673
33	1	3.571642	-5.877249	0.962857
34	8	5.611843	-4.591162	-0.247138
35	1	4.775612	-2.349435	-1.183086
36	6	-0.871479	3.375260	-0.032729
37	6	0.301093	2.703515	-0.056654
38	6	1.656307	3.238364	-0.046941
39	6	2.021897	4.468326	0.545508
40	6	3.333895	4.932981	0.486274
41	1	-0.900962	4.460533	-0.071245
42	1	0.220994	1.620960	-0.122272
43	6	2.690672	2.483846	-0.635798
44	6	3.996185	2.934595	-0.709964
45	6	4.313644	4.177278	-0.152282
46	1	3.569910	5.878475	0.962524
47	8	5.610595	4.592579	-0.246853
48	1	4.775062	2.350373	-1.182279
49	1	2.438560	1.520598	-1.066353
50	8	1.138578	5.252386	1.227210
51	1	5.714349	5.451035	0.176998
52	1	0.334390	4.746964	1.403925
53	1	5.715867	-5.449487	0.176911
54	1	0.335854	-4.746471	1.404290

Table S6. The optimized Cartesian coordinates of the *trans* rotamer **2** of **3a** calculated at the DFT/B3LYP-GD3/6-311++G(d) level of theory

Center Number	Atomic Number	Coordinates (Angstroms) X	Y	Z
1	6	-2.061974	4.530714	-0.081254
2	6	-2.798283	3.373916	-0.063376
3	6	-2.121375	2.125326	-0.026761
4	7	-0.791442	2.048634	-0.023679
5	6	-0.059266	3.176589	-0.044311
6	6	-0.650169	4.469869	-0.070969
7	6	0.167285	5.643076	-0.092586
8	6	1.524985	5.545815	-0.089547
9	6	2.168306	4.269018	-0.065389
10	6	1.396992	3.070760	-0.041095
11	6	3.572753	4.129062	-0.064447
12	6	4.133033	2.875274	-0.041289
13	6	3.283168	1.738249	-0.016628
14	7	1.957877	1.853048	-0.014303
15	1	-2.552935	5.498207	-0.108567
16	1	-3.879903	3.415216	-0.082361
17	1	-0.316954	6.613777	-0.112286
18	1	2.142445	6.437926	-0.106606
19	1	4.197462	5.016292	-0.083127
20	1	5.209518	2.745923	-0.043132
21	6	3.868897	0.393677	0.002816
22	6	3.129505	-0.736103	-0.054961
23	6	3.592373	-2.118324	-0.052728
24	6	4.775814	-2.557797	0.582648
25	6	5.174679	-3.891000	0.516387
26	1	4.954641	0.355063	-0.010982
27	1	2.054941	-0.595628	-0.145897
28	6	2.811920	-3.100151	-0.695002
29	6	3.199271	-4.425574	-0.777895
30	6	4.398284	-4.817712	-0.174380
31	1	6.085122	-4.184738	1.027588
32	8	4.750784	-6.132874	-0.278002
33	1	2.597277	-5.163479	-1.291847
34	1	1.881857	-2.790869	-1.159557
35	8	5.574178	-1.729136	1.314813
36	1	5.584430	-6.287871	0.178417
37	1	5.105600	-0.900552	1.480601
38	6	-2.811753	0.833664	0.005491
39	6	-4.144326	0.632910	0.090303
40	6	-4.852880	-0.644294	0.115572
41	6	-4.396478	-1.815378	-0.532007
42	6	-5.113784	-3.007571	-0.444009
43	6	-6.302414	-3.058374	0.277782
44	6	-6.802545	-1.908118	0.896541
45	6	-6.084007	-0.730639	0.794654
46	1	-6.468221	0.162921	1.275499
47	1	-4.792159	1.501266	0.186640
48	1	-2.119649	-0.003241	0.023339
49	8	-3.270099	-1.857309	-1.295844
50	1	-4.729373	-3.879252	-0.962488
51	8	-7.034640	-4.203842	0.401069
52	1	-7.737527	-1.960126	1.438839
53	1	-6.598827	-4.925981	-0.063704
54	1	-2.932626	-0.962650	-1.432137

Table S7. The optimized Cartesian coordinates of the mixed *cis-trans* rotamer **3** of **3a** calculated at the DFT/B3LYP-GD3/6-311++G(d) level of theory

Center Number	Atomic Number	Coordinates (Angstroms) X	Y	Z
1	6	5.519605	0.600923	-0.534442
2	6	4.833858	1.739879	-0.872642
3	6	3.409452	1.729736	-0.860660
4	7	2.731238	0.624922	-0.571235
5	6	3.390390	-0.504205	-0.255296
6	6	4.808382	-0.574824	-0.204442
7	6	5.460160	-1.796126	0.155842
8	6	4.736584	-2.911991	0.447448
9	6	3.307618	-2.896392	0.389497
10	6	2.614199	-1.704668	0.033535
11	6	2.522143	-4.035289	0.671024
12	6	1.153833	-3.959113	0.581403
13	6	0.550336	-2.730420	0.203570
14	7	1.275611	-1.642575	-0.047821
15	1	6.604720	0.590053	-0.520170
16	1	5.363471	2.649289	-1.132002
17	1	6.544343	-1.815589	0.192388
18	1	5.234442	-3.836004	0.722498
19	1	3.009032	-4.963819	0.951271
20	1	0.533292	-4.824106	0.786494
21	6	-0.907396	-2.643309	0.079590
22	6	-1.535286	-1.577484	-0.467294
23	6	-2.960227	-1.333150	-0.624461
24	6	-3.961195	-1.888145	0.205159
25	6	-5.309490	-1.620103	-0.018995
26	1	-1.458825	-3.528058	0.384465
27	1	-0.888176	-0.796431	-0.856425
28	6	-3.388024	-0.446092	-1.633017
29	6	-4.722454	-0.176299	-1.872876
30	6	-5.689281	-0.780726	-1.062607
31	1	-6.042260	-2.059428	0.649202
32	8	-6.997352	-0.492533	-1.327661
33	1	-5.035136	0.494189	-2.662622
34	1	-2.632513	0.027340	-2.250375
35	8	-3.679114	-2.682051	1.277028
36	1	-7.568757	-0.953905	-0.704773
37	1	-2.741720	-2.601926	1.496290
38	6	2.710892	2.969346	-1.210530
39	1	3.310414	3.622970	-1.839778
40	6	1.503263	3.478793	-0.861750
41	6	0.407169	3.061918	0.008361
42	6	-0.441616	4.066529	0.541448
43	6	-1.548757	3.749199	1.322496
44	6	-1.830489	2.417218	1.612813
45	6	-1.001502	1.404444	1.127662
46	6	0.088255	1.726800	0.338373
47	1	0.717355	0.938067	-0.050680
48	8	-0.235952	5.406920	0.343628
49	1	-2.157766	4.557671	1.712119
50	8	-2.899486	2.045492	2.377597
51	1	-1.231714	0.374342	1.362477
52	1	-3.415471	2.821285	2.620880
53	1	0.650494	5.557372	0.000956
54	1	1.329554	4.448950	-1.328825

Table S8. The optimized Cartesian coordinates of the *cis* rotamer **4** of **3a** calculated at the DFT/B3LYP-GD3/6-311++G(d) level of theory

Center Number	Atomic Number	Coordinates (Angstroms) X	Y	Z
1	6	-3.217188	2.687479	0.126347
2	6	-2.506582	3.907995	0.356333
3	6	-1.157655	3.971536	0.182138
4	6	-0.413894	2.828888	-0.255447
5	6	-1.078817	1.603929	-0.530084
6	6	-2.517462	1.525076	-0.305921
7	6	-4.609270	2.561494	0.321108
8	6	-5.219978	1.341875	0.134904
9	6	-4.425657	0.229681	-0.242036
10	7	-3.126066	0.346682	-0.482449
11	1	-3.061979	4.780210	0.685144
12	1	-0.621628	4.895528	0.372689
13	6	0.984953	2.856405	-0.445538
14	6	1.638537	1.729176	-0.877237
15	6	0.884754	0.556963	-1.143926
16	7	-0.434862	0.515000	-0.984743
17	6	-5.021720	-1.113100	-0.360830
18	6	-4.364641	-2.291720	-0.305179
19	1	-4.947129	-3.186788	-0.515115
20	1	-6.094710	-1.139276	-0.531869
21	1	-6.285782	1.221369	0.293385
22	1	-5.185246	3.427420	0.631171
23	1	1.533472	3.772261	-0.250877
24	1	2.709201	1.732001	-1.028549
25	6	1.483141	-0.670421	-1.697344
26	6	2.737456	-1.140556	-1.564727
27	6	3.840304	-0.605876	-0.742947
28	6	3.700880	-0.279515	0.627246
29	6	4.761945	0.290215	1.330201
30	6	5.980883	0.514982	0.701381
31	6	6.163821	0.149331	-0.636342
32	6	5.099354	-0.412956	-1.328593
33	1	2.986839	-2.024089	-2.151769
34	1	0.777896	-1.251423	-2.283817
35	8	2.556520	-0.475621	1.327903
36	1	2.013996	-1.184306	0.939738
37	1	4.635274	0.533417	2.376955
38	1	5.231916	-0.683404	-2.371075
39	1	7.119576	0.305468	-1.125891
40	8	6.975284	1.079749	1.452659
41	1	7.774692	1.164708	0.923087
42	6	-2.938311	-2.516691	0.000301
43	6	-2.320331	-1.981838	1.151468
44	6	-0.959041	-2.144772	1.376402
45	6	-0.193534	-2.841379	0.453277
46	6	-0.778148	-3.431501	-0.665928
47	6	-2.145049	-3.272136	-0.867172
48	1	-2.603967	-3.705997	-1.748983
49	1	-0.176608	-3.991564	-1.373992
50	1	-0.501273	-1.703062	2.251884
51	8	-3.006468	-1.285883	2.099243
52	8	1.162576	-2.911098	0.698629
53	1	-3.936509	-1.233694	1.847157
54	1	1.602681	-3.333099	-0.047538

Table S9. The optimized Cartesian coordinates of the *cis* rotamer **5** of **3a** calculated at the DFT/B3LYP-GD3/6-311++G(d) level of theory

Center Number	Atomic Number	Coordinates (Angstroms) X	Y	Z
1	6	-1.049052	1.989916	-1.293198
2	6	-2.436782	1.789709	-1.130283
3	6	-3.062258	0.625800	-1.568841
4	6	-2.301583	-0.370985	-2.164460
5	6	-0.943817	-0.180349	-2.407481
6	6	-0.341683	0.990105	-1.974435
7	1	0.715050	1.123340	-2.141753
8	8	-3.145882	2.769004	-0.490866
9	1	-4.116863	0.456224	-1.386169
10	8	-2.938093	-1.563571	-2.447226
11	1	-0.352567	-0.959739	-2.874871
12	1	-4.045740	2.466730	-0.333599
13	6	-1.351954	-2.746528	0.149469
14	6	-1.873654	-1.540787	0.778826
15	6	-3.282043	-1.398680	0.870639
16	6	-3.863603	-0.406981	1.655035
17	1	-2.139843	-3.468691	-0.049142
18	6	-1.101742	-0.530579	1.390671
19	6	-1.670131	0.480337	2.142762
20	6	-3.056464	0.520678	2.309068
21	1	-4.946361	-0.353768	1.701750
22	8	-3.574374	1.521286	3.083408
23	1	-1.059049	1.250448	2.595414
24	1	-0.035100	-0.536718	1.228195
25	8	-4.147202	-2.211995	0.181448
26	1	-4.525764	1.404322	3.175445
27	1	-3.831393	-2.287792	-0.732736
28	6	-0.119923	-3.241673	-0.143908
29	6	4.320971	2.466191	0.344327
30	6	3.214820	3.257802	0.154090
31	6	1.946938	2.649121	-0.064802
32	7	1.802266	1.327699	-0.026509
33	6	2.875305	0.542113	0.153605
34	6	4.188780	1.060172	0.328601
35	6	5.302416	0.172986	0.463357
36	6	5.130689	-1.177340	0.405709
37	6	3.829021	-1.744449	0.232725
38	6	2.687340	-0.903797	0.133747
39	6	3.603800	-3.136873	0.144733
40	6	2.325772	-3.613243	-0.006282
41	6	1.233765	-2.697744	-0.039809
42	7	1.438166	-1.384871	0.011938
43	1	5.300486	2.908916	0.494425
44	1	3.300713	4.338370	0.151533
45	1	6.291571	0.597663	0.600004
46	1	5.981235	-1.845545	0.492035
47	1	4.446596	-3.818564	0.199221
48	1	2.137321	-4.678450	-0.075474
49	1	-0.126956	-4.275314	-0.482485
50	6	0.787025	3.509067	-0.320068
51	6	-0.451968	3.224716	-0.783626
52	1	-1.143119	4.062621	-0.769064
53	1	0.961190	4.552660	-0.070361
54	1	-2.289935	-2.186542	-2.795514

Table S10. The optimized Cartesian coordinates of the *cis* rotamer **6** of **3a** calculated at the DFT/B3LYP-GD3/6-311++G(d) level of theory

Center Number	Atomic Number	Coordinates (Angstroms) X	Y	Z
1	6	-1.654854	-0.086909	-2.287959
2	6	-2.772778	0.597611	-1.997901
3	6	-3.624944	0.429370	-0.805670
4	6	-3.110514	0.413824	0.508474
5	6	-5.016511	0.313284	-0.957685
6	6	-5.872465	0.122459	0.114734
7	6	-5.331780	0.069909	1.402858
8	6	-3.964458	0.225643	1.598619
9	1	-5.428171	0.353697	-1.960848
10	1	-6.941196	0.018147	-0.019843
11	1	-3.532340	0.222484	2.593653
12	1	-1.063971	0.206942	-3.150745
13	1	-3.086415	1.368578	-2.698532
14	6	-1.918160	-2.309930	-1.125483
15	6	0.752840	-2.148567	-0.507143
16	6	0.031080	-3.311792	-0.124940
17	6	-1.341375	-3.359357	-0.453294
18	7	0.189599	-1.150098	-1.216469
19	6	-1.102674	-1.217311	-1.508972
20	6	2.146122	-2.024053	-0.103736
21	6	0.694261	-4.379288	0.560674
22	6	2.014845	-4.291676	0.877365
23	6	2.765729	-3.114786	0.565458
24	7	2.786967	-0.868616	-0.353876
25	6	4.128091	-2.966863	0.907675
26	6	4.774058	-1.787183	0.633871
27	6	4.050946	-0.723067	0.025489
28	1	-2.970491	-2.308703	-1.377754
29	1	-1.928714	-4.226735	-0.169977
30	1	0.121743	-5.263533	0.820479
31	1	2.515242	-5.106877	1.389612
32	1	4.648141	-3.784702	1.395973
33	1	5.815457	-1.649503	0.901007
34	6	4.725337	0.567912	-0.138603
35	6	4.214764	1.818758	-0.186376
36	6	2.843050	2.343039	-0.170667
37	6	2.578703	3.485809	0.617137
38	6	1.338128	4.117147	0.600101
39	6	0.330955	3.620512	-0.222979
40	6	0.559048	2.491325	-1.011766
41	6	1.792385	1.860109	-0.970124
42	1	4.975028	2.601754	-0.217607
43	1	1.179279	4.976532	1.242196
44	1	1.949902	0.969345	-1.557162
45	1	-0.229158	2.130470	-1.658022
46	1	5.810506	0.502183	-0.128666
47	8	3.518407	4.029879	1.451401
48	8	-0.908706	4.193056	-0.293496
49	1	4.252221	3.414006	1.555142
50	1	-0.964334	4.935510	0.317062
51	8	-1.800892	0.590651	0.820974
52	8	-6.201935	-0.122103	2.440984
53	1	-1.273029	0.834295	0.048274
54	1	-5.719624	-0.130328	3.274318
